# Porphyrin-based metallo-organic cage for selective photocatalysis under heterogeneous and atmospheric conditions

**DOI:** 10.1039/d6sc03415b

**Published:** 2026-07-17

**Authors:** Qixia Bai, Gang Chen, Tun Wu, Wei Zhang, Huoqing Chen, Tian Li, Xiang Li, Yu-Ming Guan, Zhihong Chen, Ming Wang, Pingshan Wang, Zhe Zhang

**Affiliations:** a Key Laboratory for Water Quality and Conservation of the Pearl River Delta (Ministry of Education), Institute of Environmental Research at Greater Bay Area, Guangzhou University Guangzhou 510006 China chemwt@gzhu.edu.cn chemwps@gzhu.edu.cn zhezhang2018@gzhu.edu.cn; b College of Materials Science and Engineering, Shanxi Normal University Taiyuan 030000 China; c School of Chemistry and Chemical Engineering, Guangdong Provincial Key Laboratory of Optoelectronic Materials and Sensor Components, Guangzhou Key Laboratory of Sensing Materials & Devices, Centre for Advanced Analytical Science, Guangzhou University Guangzhou 510006 China; d School of Physics and Materials Science, Guangzhou University Guangzhou 510006 China; e State Key Laboratory of Supramolecular Structure and Materials, College of Chemistry, Jilin University Changchun 130012 China mingwang358@jlu.edu.cn

## Abstract

Developing highly efficient and stable photocatalytic systems for selective oxidation reactions and biomimetic catalysis represents both a frontier and a challenge in current research. This work successfully designed and synthesized a novel porphyrin-based metallo-organic cage (MOC), Por-Cage, which has been thoroughly characterized by proton nuclear magnetic resonance (^1^H NMR) spectroscopy, electrospray ionization mass spectrometry (ESI-MS), and single-crystal X-ray diffraction (SC-XRD). Photophysical and photoelectrochemical studies reveal that, compared to ligand L and tetraphenyltetrapyrrole (TPP), Por-Cage exhibits superior light absorption, significantly enhanced photogenerated charge separation efficiency, and reactive oxygen species (ROS) production due to its unique supramolecular architecture. Using the photocatalytic detoxification of 2-chloroethyl ethyl sulfide (CEES) as a model reaction, Por-Cage demonstrated outstanding heterogeneous catalytic activity, achieving highly efficient degradation with 100% selectivity under an air atmosphere after 20 minutes of illumination. Furthermore, it exhibited universal applicability toward the oxidation of sulfides in aromatic compounds. This study provides a novel design strategy for constructing high-performance MOC photocatalysts that eliminate the dependence on pure oxygen and demonstrates their potential applications in environmental detoxification and selective photocatalytic reactions.

## Abstract 2

In this study, we report a porphyrin-based metallo-organic cage (Por-Cage) for efficient, selective photocatalytic detoxification and sulfide oxidation under air, offering a new strategy for high-performance porphyrin photocatalysts.

## Graphical abstract

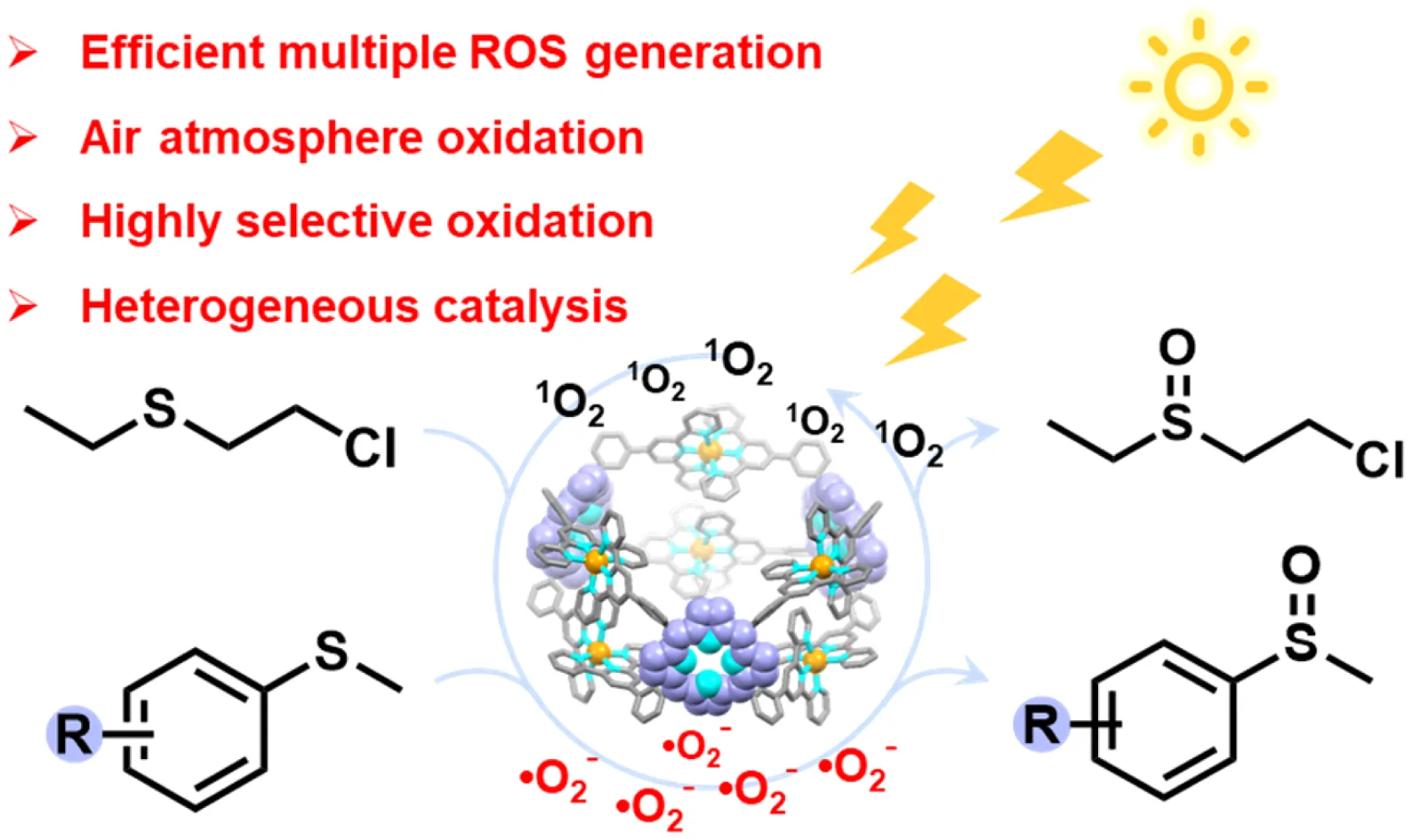

## Introduction

Photosensitizers are a group of functional molecules capable of absorbing light energy at specific wavelengths and converting it into chemical energy or thermal energy, thereby triggering or promoting chemical reactions. They act as “energy transporters”, transferring their energy to other reactants or generating active intermediates (such as free radicals and singlet oxygen) to drive the reaction forward.^1–3^ Significant applications are included in fields such as photocatalytic synthesis,^4–7^ environmental pollutant degradation,^8–11^ and photodynamic therapy (PDT).^12–15^ Among these, visible-light-driven selective oxidation reactions, such as the conversion of sulfides to sulfoxides and the hydroxylation of aromatic hydrocarbons, have garnered significant attention,^16–18^ enabling efficient, highly selective conversion of specific functional groups under mild conditions, avoiding the high energy consumption, significant pollution, and numerous byproducts associated with traditional high-temperature/high-pressure methods or stoichiometric oxidants and aligning with the principles of green chemistry and atom economy.^19,20^ Currently, commonly used photosensitizers primarily include organic dyes (*e.g.*, rose bengal and methylene blue),^21,22^ metal complexes (*e.g.*, ruthenium bipyridine complexes),^23–25^ and certain inorganic semiconductor materials (*e.g.*, TiO_2_ and ZnO).^26–28^ These materials generate electron–hole pairs upon photoexcitation, subsequently producing reactive species such as superoxide anions (·O_2_^−^)^29,30^ and hydroxyl radicals (·OH) through oxygen reduction. Alternatively, their photosensitization process enables energy transfer and subsequent singlet oxygen (^1^O_2_) production.^31,32^ These reactive oxygen species (ROS) can effectively facilitate the oxidation reactions. However, photosensitizers generally exhibit high oxygen dependency, often requiring pure oxygen atmospheres for efficient catalysis. This not only substantially elevates the complexity and operational costs of reaction systems but also introduces potential safety risks, severely restricting their practical applications.^33–35^ Consequently, the development of novel photosensitizers that can perform efficient selective oxidation under atmospheric conditions, while simultaneously exhibiting broad-spectrum absorption, high photostability, and excellent catalytic selectivity, has become a critical challenge demanding breakthroughs in this field.

Metallo-organic cages (MOCs) are a class of discrete supramolecular entities formed through coordination-driven self-assembly of organic ligands and metal ions.^36–43^ Their structures can be precisely tuned at the nanoscale through careful design of ligand symmetry, coordination geometry, and metal nodes, enabling customizable shape, size and catalytic microenvironments.^44–49^ As photosensitizers, MOCs not only inherit the precisely defined energy level structure and tunable photophysical properties of molecular materials but also exhibit enhanced light absorption, facilitated exciton separation, and prolonged charge lifetime due to their cavity confinement effect and periodic metal–ligand charge transfer (MLCT) characteristics.^50–53^ In 2024, Zhang *et al.* designed a porphyrin-based platinum metallo-organic cage, which can efficiently generate ^1^O_2_*via* excited-state energy transfer, while its C_70_ clathrate primarily produces superoxide radicals (·O_2_^−^) through electron transfer under illumination. This enables tunable ROS generation for selective photocatalytic oxidation of benzyl alcohol.^54^ P. S. Mukherjee *et al.* developed a water-soluble Pd_16_L_8_ metallo-organic cage for the selective aerobic oxidation of aryl sulfides. Under visible light in aqueous media, this cage demonstrated outstanding catalytic efficiency for the highly selective conversion of alkyl and aryl sulfides into corresponding sulfur oxides.^55^ However, most MOC photocatalysts currently primarily operate under homogeneous conditions, which have obvious shortcomings in aspects such as catalyst separation, selectivity, and stability. When applied to heterogeneous catalytic systems, they often suffer from aggregation-caused fluorescence quenching (ACQ), leading to a significant decline or even loss of photosensitivity.

**Scheme 1:**
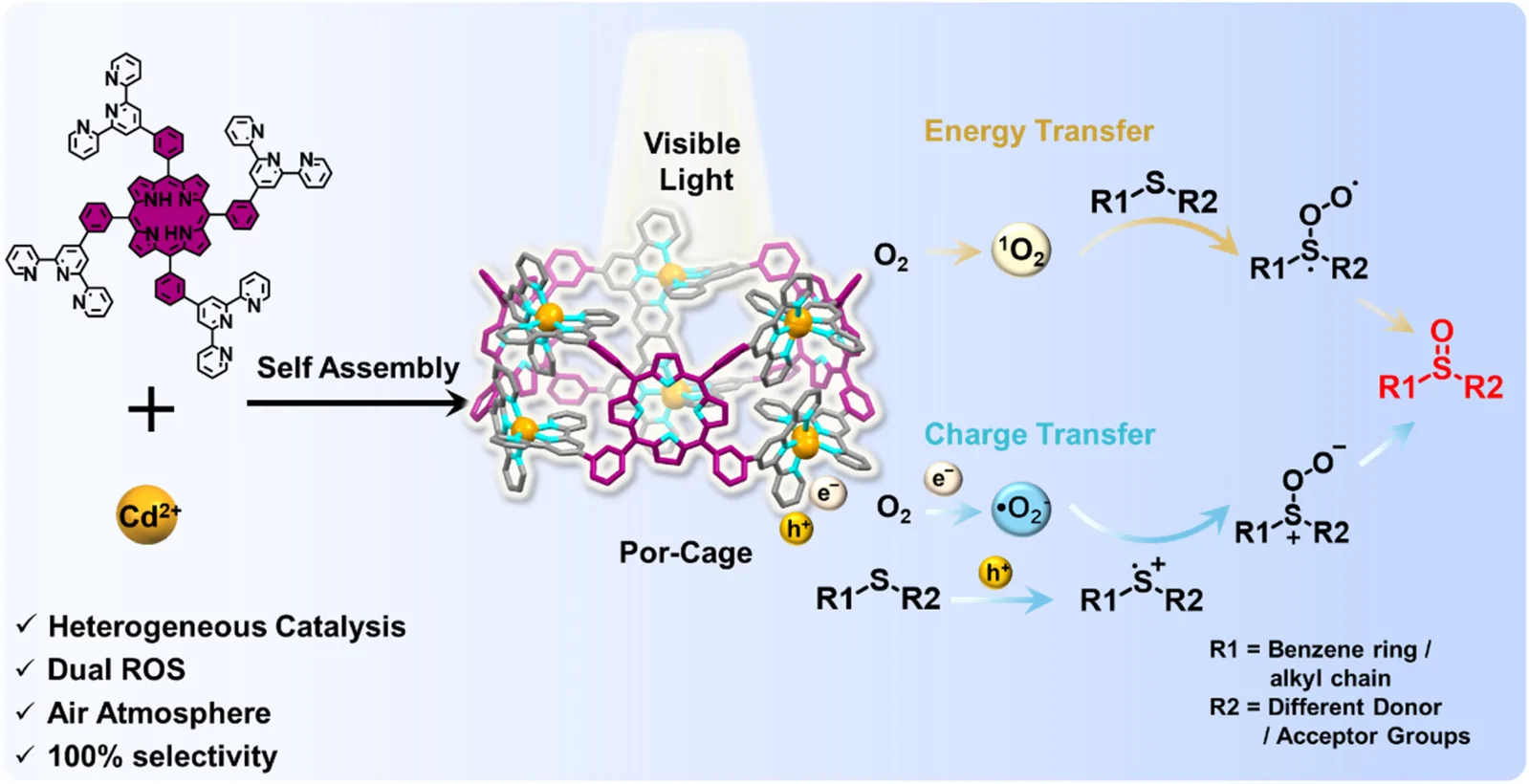
Scheme of air atmosphere catalysis in metallo-organic cages.

Porphyrin compounds constitute a prototypical class of photosensitizers, offering advantages such as broad-spectrum visible-light absorption, high singlet oxygen quantum yield, and prolonged excited-state lifetimes. These advantageous photophysical properties have enabled their widespread application in diverse fields, including photodynamic therapy, photocatalytic organic synthesis, and environmental remediation. The large conjugated π systems and tunable metal centers also make them ideal photoactive building blocks.^56–60^ However, their inherent tendency toward ACQ and poor photostability limit their application in heterogeneous systems. To address these issues, we envisioned the incorporation of porphyrin units into a rigid MOC framework to achieve photosensitizer site isolation, which can effectively suppress aggregation, enhance photostability and electron separation efficiency. Based on this concept, a novel porphyrin-based MOC, Por-Cage, has been designed and synthesized in which three porphyrin units were connected by 〈Tpy–Cd^2+^–Tpy〉 coordination bonds (Tpy = 2,2′:6,2″-terpyridine) and formed a highly symmetric truncated trigonal prism configuration. Systematical characterization was performed using ^1^H NMR, ESI-MS, and SC-XRD. Electron paramagnetic resonance (EPR) test results indicate that Por-Cage not only generates ^1^O_2_ but also produces ·O_2_^−^. The synergistic action of these two reactive oxygen species endows it with excellent photocatalytic oxidation performance. Furthermore, using the oxidation of 2-chloroethyl ethyl sulfide (CEES) as a model reaction, its heterogeneous photocatalytic performance was evaluated, demonstrating that it can selectively oxidize CEES into 2-chloroethyl ethyl sulfoxide (CEESO) within 20 min under an air atmosphere. The catalytic mechanism study revealed that coordination-driven self-assembly effectively promoted the intersystem crossing ability, extended the triplet lifetime and improved the charge separation ability of Por-Cage, facilitating its ^1^O_2_ and ·O_2_^−^ generation efficiency upon photoirradiation, in contrast to the control compounds tetraphenylporphyrin (TPP) and corresponding ligand L (Scheme 1). This study not only provides a novel strategy for constructing high-performance porphyrin-based photocatalytic systems but also offers important insights into the structure–performance relationship in supramolecular photocatalysis.

## Experimental section

### Materials

Chemicals were purchased from Sigma-Aldrich, Energy Chemical, and Bidepharm and used without further purification. Thin-layer chromatography (TLC) was conducted on flexible sheets (Baker-flex) precoated with Al_2_O_3_ (IB-F) or SiO_2_ (IB2-F). Column chromatography was conducted using basic Al_2_O_3_ Brockman Activity I (60–325 mesh) or SiO_2_ (60–200 mesh) from Fisher Scientific.

### Synthesis of Por-Cage

**Fig. 1:**
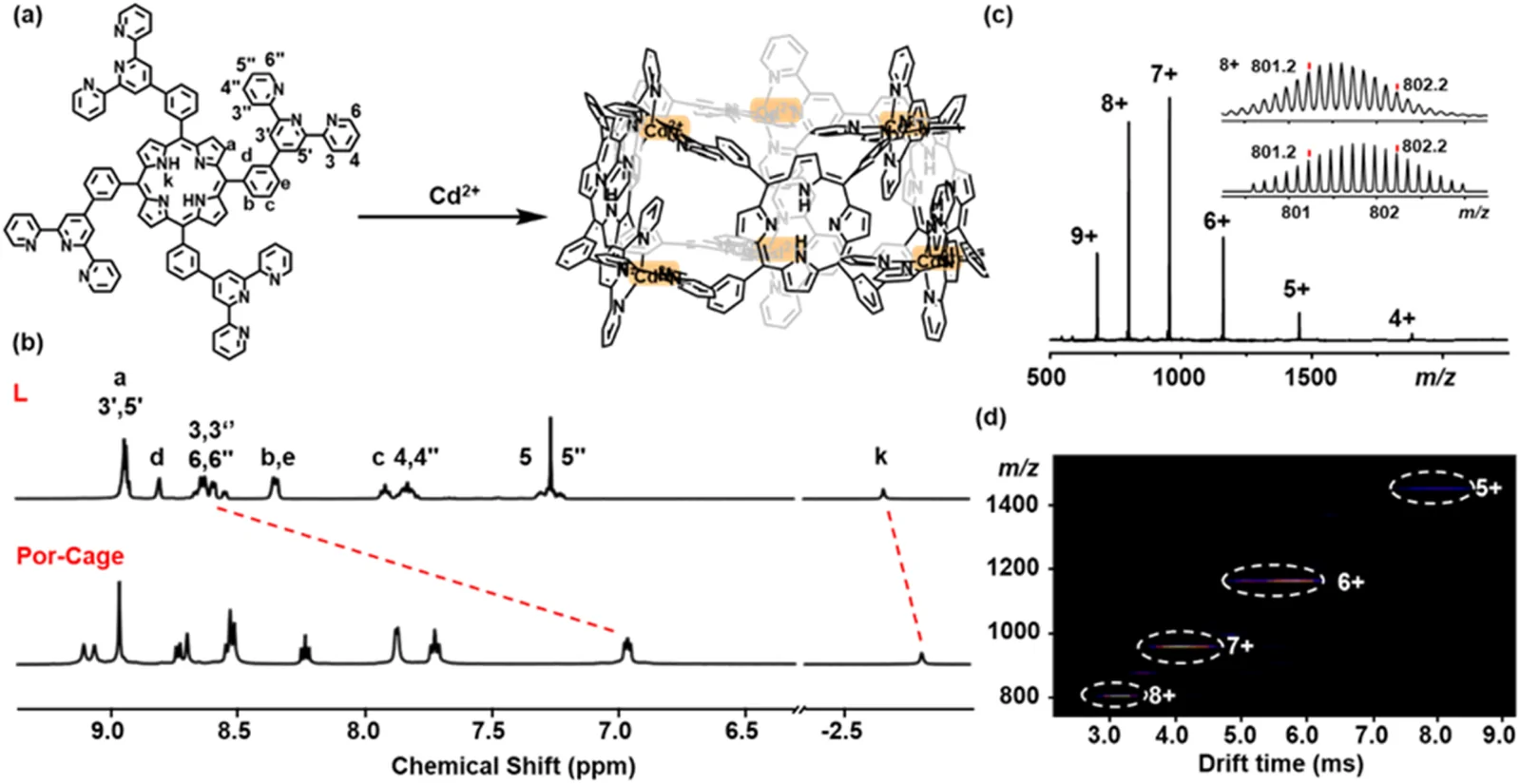
Synthesis and characterization of Por-Cage. (a) Self-assembly of Por-Cage; (b) ^1^H NMR spectrum of ligand L in CDCl_3_ (400 MHz, 300 K) and Por-Cage in CD_3_CN (500 MHz, 300 K); (c) ESI-MS spectra of Por-Cage, with the inset showing observed and modelled isotopic patterns of the 8+ charge state; (d) TWIM-MS plot of Por-Cage.

First, ligand L (10.00 mg, 6.49 µmol) and Cd(NO_3_)_2_·4H_2_O (4.00 mg, 12.98 µmol) were reacted in a mixed solvent (CHCl_3_ : CH_3_OH = 10 mL : 10 mL) at 60 °C for 8 hours. Upon cooling to room temperature, an excess of LiNTf_2_ was added until a distinct precipitate formed. The mixture was transferred to a centrifuge tube and centrifuged. The supernatant was removed, and the residue was washed three times with a mixture of distilled water and methanol. The washed residue was dried in an oven, yielding 13.72 mg of purple solid (98% yield) (Scheme S1 and Fig. 1a). ^1^H NMR (500 MHz, CD_3_CN, 300 K) *δ* 9.10–9.05 (d, *J* = 20.0 Hz, 8H, Por-H^a^), 8.97 (s, 8H, Tpy-H^3′,5′^), 8.75–8.72 (d, *J* = 15.0 Hz, 4H, Ph-H^b^), 8.70 (s, 4H, Ph-H^e^), 8.56–8.50 (t, *J* = 15.0 Hz, 12H, Tpy-H^3,3″^, Ph-H^d^), 8.26–8.21 (t, *J* = 12.5 Hz, 4H, Ph-H^c^), 7.90–7.85 (d, *J* = 25.0 Hz, 8H, Tpy-H^6,6″^), 7.74–7.69 (t, *J* = 12.5 Hz, 8H, Tpy-H^4,4″^), 6.99–6.94 (m, 8H, Tpy-H^5,5″^), −2.80 (s, 2H, Por-NH^k^). ESI-TOF (*m*/*z*): 2605.01[M-3NTf_2_^−^]^3+^ (calcd *m*/*z*: 2605.01), 1883.73[M-4NTf_2_^−^]^4+^ (calcd *m*/*z*: 1883.73), 1450.95[M-5NTf_2_^−^]^5+^ (calcd *m*/*z*: 1450.95), 1162.43 [M-6NTf_2_^−^]^6+^ (calcd *m*/*z*: 1162.44), 956.35 [M-7NTf_2_^−^]^7+^ (calcd *m*/*z*: 956.35), 801.79 [M-8NTf_2_^−^]^8+^ (calcd *m*/*z*: 801.79), 681.58 [M-9NTf_2_^−^]^9+^ (calcd *m*/*z*: 681.58).

### Characterization

NMR spectra were recorded on Bruker NMR 400 or 500 MHz spectrometers, using CDCl_3_ for the ligand and CD_3_CN for the MOC. ESI mass spectrometry (MS) experiments were performed on a Waters Synapt HDMS G2-Si quadrupole/time-of-flight (Q/TOF) tandem mass spectrometer. UV-vis absorption spectra were recorded on a Thermo Fisher Scientific Evolution 201 spectrophotometer at room temperature (10^−6^ M in CHCl_3_ or CH_3_CN). Mott–Schottky photoelectrochemical measurements were performed using a CHI 760D electrochemical workstation. EPR spectra were measured using a Bruker A300 electron paramagnetic resonance spectrometer with 4-oxo-TEMPO or 5,5-dimethyl-1-pyrroline-*N*-oxide as a free radical trapping agent added to the material dispersion. A UV-3600 UV-vis diffuse reflectance spectrometer was used to evaluate the wavelength range of light absorption of the materials. X-ray photoelectron spectroscopy (XPS) measurements were performed using a Thermo Scientific K-Alpha X-ray photoelectron spectrometer to analyze the composition and bonding information of the samples (Thermo Nexsa, USA). Fs-transient absorption spectroscopy measurements were performed using a custom-built measurement system. This system was driven by a commercial femtosecond (fs) laser operating at a repetition rate of 1 kHz, with a pulse duration of ∼170 fs and a wavelength of 800 nm.

## Results and discussion

### Characterization of the metallo-organic cage

As shown in Fig. 1b, ligand L exhibits a sharp peak splitting in the aromatic region, where the N–H^k^ proton within the porphyrin ring exhibits a characteristic singlet at −2.66 ppm. Upon coordination with Cd^2+^ ions, the ring proton Por-NH^k^ shifts upfield at −2.80 ppm (Δ*δ* = 0.14 ppm). Similarly, the Tpy-H^6,6″^ proton in the terpyridine moiety exhibits a significant upfield shift from 8.64 ppm to 6.96 ppm (Δ*δ* = 1.68 ppm) due to a slight shielding effect caused by coordination^61^ (Fig. 1b and S2–S11). This characteristic change and clear peaks provide direct evidence for the generation of a highly symmetric structure. Subsequently, the composition of the product was confirmed by ESI-MS (Fig. 1c), displaying a set of intense peaks in a normal distribution at *m*/*z* = 681.58, 801.76, 956.35, 1162.43, 1450.95, and 1883.73, corresponding to 9+ to 4+ ions. The experimental molecular weight precisely matches the [Cd_6_L_3_] composition consisting of three ligands L, six Cd^2+^ ions, and twelve NTf_2_^−^. The TWIM-MS spectrum exhibits only one series of step-like narrow drift time distributions from 8+ to 5+ ions, indicating the absence of other isomers or conformers in the system (Fig. 1d and S1).^62^

**Fig. 2:**
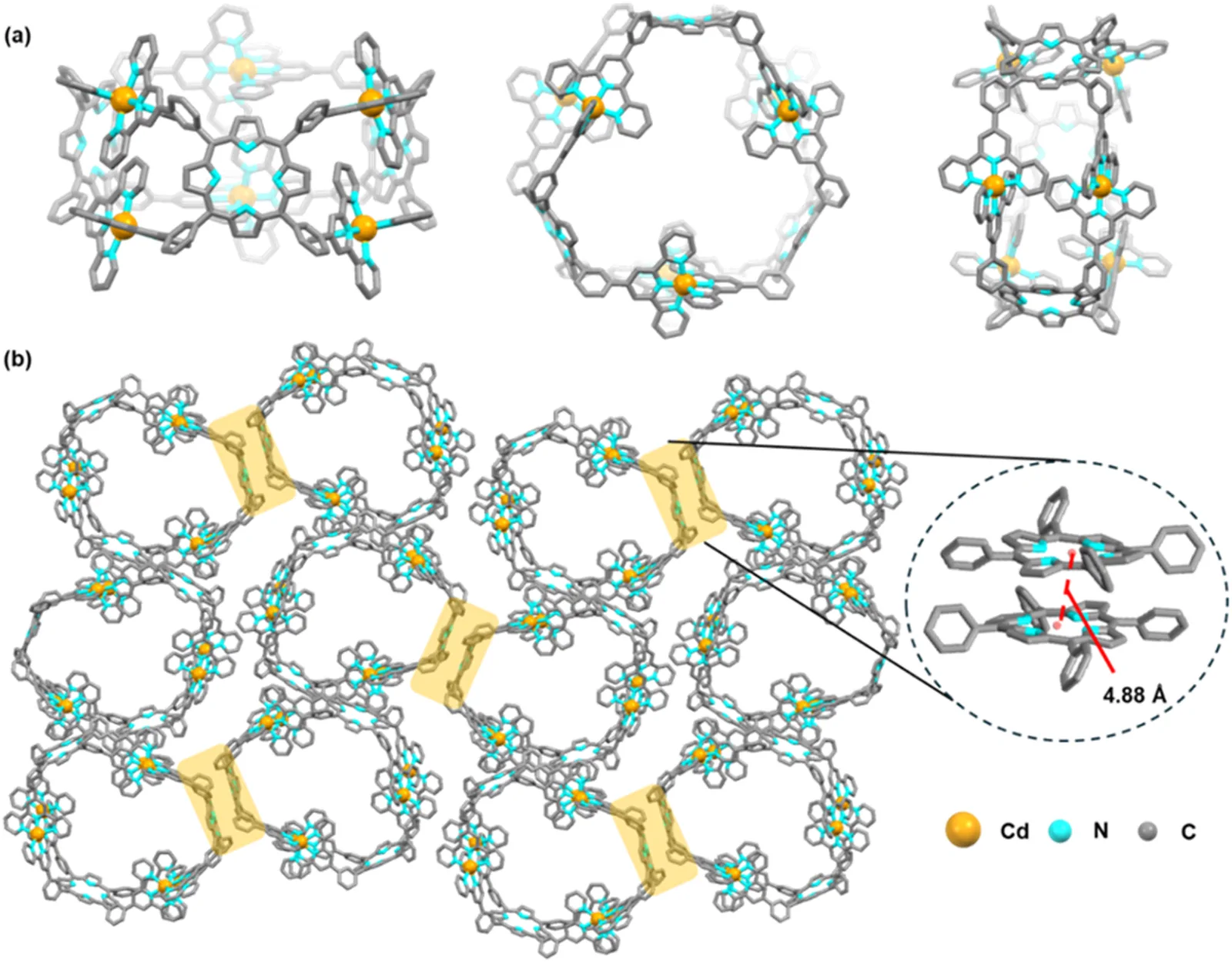
Single-crystal structure of Por-Cage. (a) Crystal structures of Por-Cage at different angles; (b) crystal structure stacking diagram of Por-Cage.

Furthermore, dark red transparent prismatic crystals suitable for X-ray crystallography were obtained after slowly diffusing isopropyl ether into the acetonitrile solution of Por-Cage at a constant temperature of 15 °C for two weeks. The Por-Cage crystal crystallized in the monoclinic space group *P*2_1_/*c*. As depicted in Fig. 2a, this MOC exhibits a truncated trigonal prism configuration with *D*_3h_ symmetry, in which three porphyrin planes form the three vertices of the prism and the upper and lower 〈Tpy–Cd^2+^–Tpy〉 arms constituting the three rectangular faces of the prism. This highly symmetric structure is fully consistent with the results from NMR and ESI-MS analyses, providing crystallographic evidence for the precise construction of the MOC (Fig. S46 and Table S4). Further analysis of Por-Cage's changes during the stacking process reveals that adjacent porphyrin groups exist in a mutually parallel manner, separated by a distance of 4.88 Å.

To further characterize the solid-state morphology structure of Por-Cage, SEM, TEM, and PXRD measurements were conducted. The SEM and TEM images reveal the morphology of the isolated Por-Cage solid (Fig. S47–S49). PXRD was further conducted to examine the long-range ordering of the isolated Por-Cage powder. No sharp diffraction peaks were observed, suggesting that the precipitated and dried bulk powder possesses poor crystallinity or largely amorphous packing (Fig. S50).

### Investigation of the optical and electrochemical properties of the metallo-organic cage

**Fig. 3:**
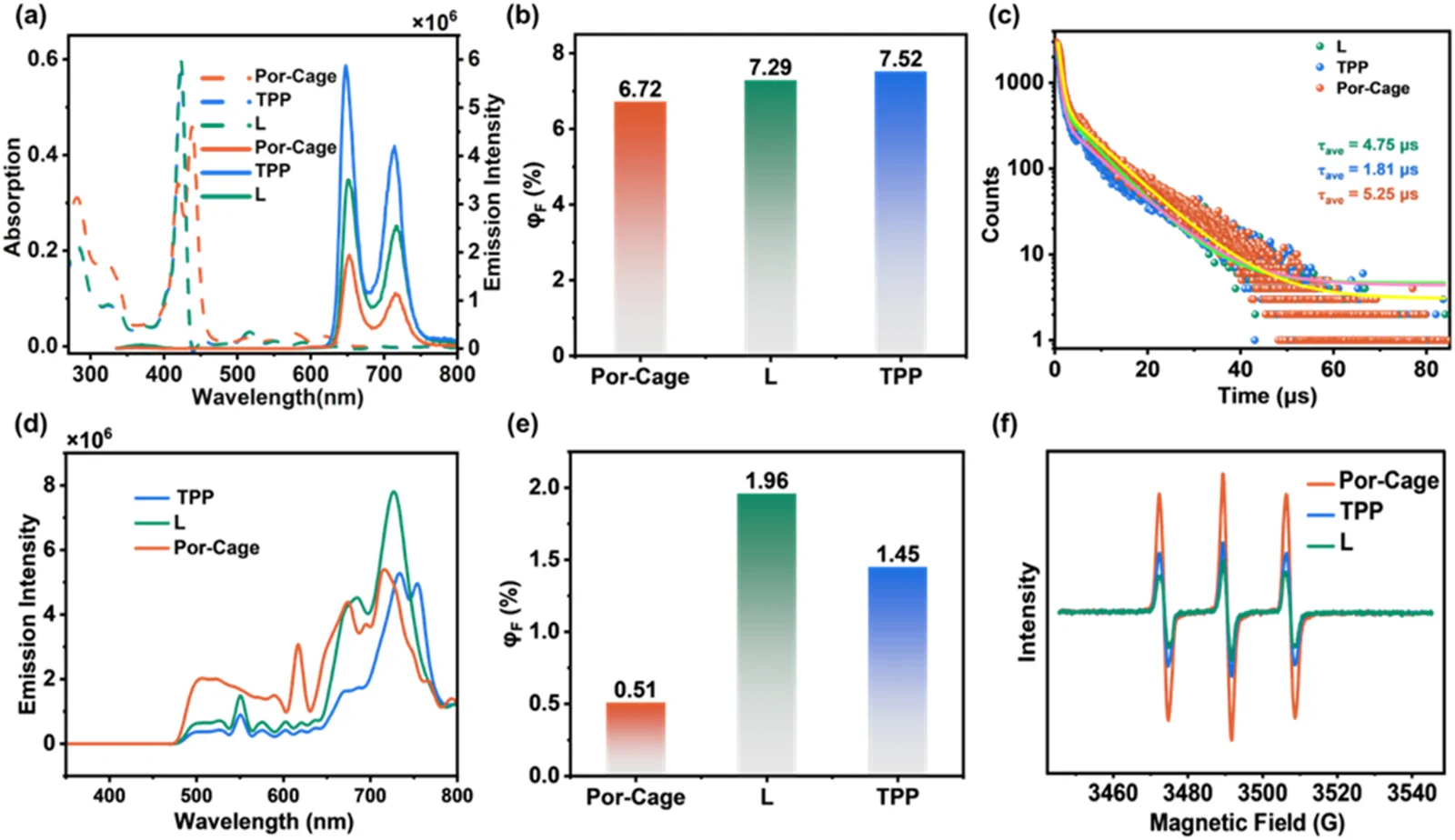
Photophysical properties of Por-Cage. (a) UV-vis and fluorescence spectra of Por-Cage in CH_3_CN and ligand L and TPP in CHCl_3_ at the same concentration (porphyrin unit = 1 × 10^−6^ M); (b) fluorescence quantum yields of Por-Cage, ligand L, and TPP; (c) fluorescence lifetime fitting curves for Por-Cage, ligand L and TPP in the solid state; (d) fluorescence spectra of Por-Cage and ligand L and TPP in the solid state; (e) fluorescence quantum yields of Por-Cage, ligand L, and TPP in the solid state; (f) EPR spectra of Por-Cage, ligand L and TPP mixed with 4-oxo-TEMPO under visible light irradiation.

Following comprehensive structural characterization of Por-Cage, considering the enrichment of photosensitive units within the cage structure, we conducted detailed optical investigations using ligand L and tetraphenylporphyrin (TPP) as controls. Fig. 3a displays the UV absorption spectra of the three materials (dashed lines) in CH_3_CN or CHCl_3_, all exhibiting characteristic absorption peaks at 423 nm attributable to the porphyrin moiety. In contrast, Por-Cage shows a new absorption peak at 438 nm, indicating charge transfer within the cage structure after coordination. Such charge transfer not only enhances light absorption efficiency but may also influence electron excitation and transfer during photocatalysis. Similarly, the fluorescence spectra (solid lines) of all three materials exhibit maximum emissions at 652 nm and 717 nm, attributable to porphyrin groups. With increasing porphyrin units, fluorescence intensity decreases, accompanied by slight red shifts and broadening of the emission. This is probably due to the increased degree of conjugation and enhanced electron interaction. The corresponding fluorescence quantum yield decreased from 7.52% for TPP and 7.29% for ligand L to 6.72% for Por-Cage (Fig. 3b). Furthermore, fluorescence lifetime curve fitting for the three materials revealed a gradual increase in lifetime from 1.81 µs to 4.75 µs, and subsequently to 5.25 µs (Fig. 3c and S12). Additionally, solid-state fluorescence emission and quantum yields were measured for Por-Cage, ligand L, and TPP, showing extremely weak fluorescence intensity in the solid state, with corresponding quantum yields ranging from 1.96% for TPP to 1.49% for ligand L and 0.59% for Por-Cage (Fig. 3d and e). All these changes in optical properties indicate a reduction in the energy allocated to fluorescence emission, which favors competitive photosensitization processes and promotes the generation of ^1^O_2_. Moreover, employing the EPR technique, the production of ^1^O_2_ was examined for the three materials dispersed as powders in water under illumination. Using the standard probe 4-oxo-TEMPO, a characteristic 1 : 1 : 1 triplet belonging to ^1^O_2_ was observed (Fig. 3f). And the signal intensity for Por-Cage was significantly higher than those for ligand L and TPP, indicating the higher ^1^O_2_ production of Por-Cage under illumination in the solid state (Fig. S25).

**Fig. 4:**
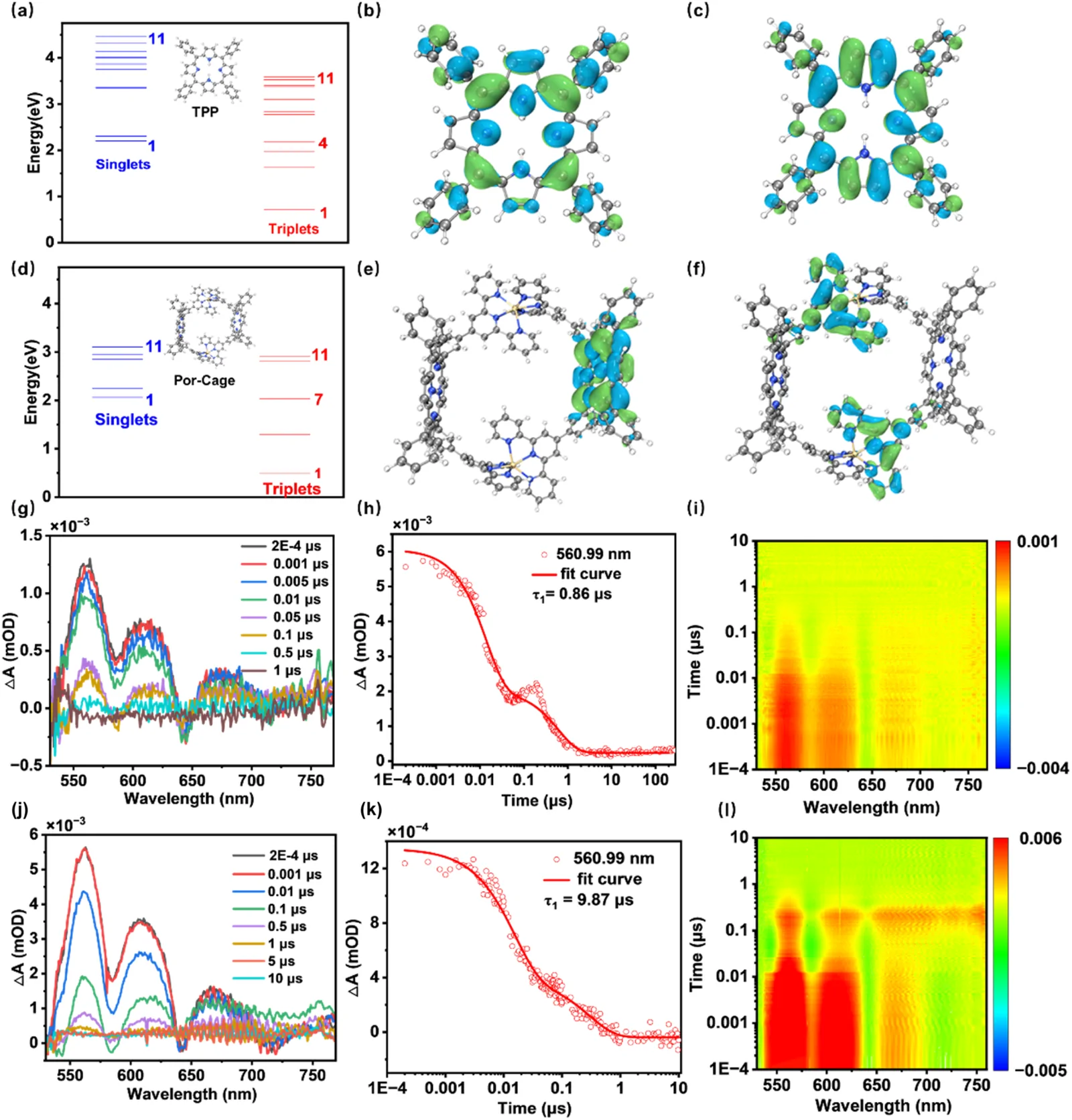
DFT calculations and transient absorption spectroscopy. (a) The energy-minimized structure of TPP and its singlet and triplet energy levels; (b) and (c) the HOMO and LUMO wavefunctions of the geometrically-optimized structure of TPP computed by TD-DFT; (d) the energy-minimized structure of Por-Cage and its singlet and triplet energy levels; (e) and (f) the HOMO and LUMO wavefunctions of the geometrically-optimized structure of Por-Cage computed by TD-DFT; (g) Ns-transient absorption (TA) spectra of TPP in CH_3_CN at various time delays after excitation at 515 nm; (h) decay kinetic curves of TPP at 560 nm; (i) 2D contour plot of ns-TA for TPP; (j) Ns-transient absorption (TA) spectra of Por-Cage in CH_3_CN at various time delays after excitation at 515 nm; (k) decay kinetic curves of Por-Cage at 560 nm; (l) 2D contour plot of ns-TA for Por-Cage.

Transient absorption (TA) spectroscopy was used to investigate the triplet lifetime of TPP and Por-Cage. As shown in Fig. 4, the triplet state of TPP decays with a lifetime of ∼1 µs on account of collisional quenching with other porphyrin triplets in acetonitrile. For Por-Cage, its intrinsic triplet lifetime is determined to be ∼10 µs, presumably owing to the inhibition of collisional quenching by photosensitizer site isolation. The extended triplet lifetime of Por-Cage benefits the energy transfer process from the triplet state to oxygen, thus facilitating ^1^O_2_ production (Fig. 4g–n and S27). Subsequently, combination of TD-DFT calculations with singlet–triplet energy level calculations further confirmed the mechanism behind the efficient ^1^O_2_ generation in Por-Cage. Triplet–singlet energy level calculations were performed for both Por-Cage and TPP, exhibiting significant difference in intersystem crossing (ISC) efficiency. It is observed in Fig. 4a that TPP possesses only four ISC pathways from S1 to T*n* (*n* = 4). In contrast, with the enrichment of porphyrin groups, Por-Cage exhibits a substantially greater number of ISC pathways than TPP (S1 to T*n*, *n* = 7), thereby significantly enhancing the generation efficiency of ^1^O_2_ (Fig. 4d). The molecular orbital analysis reveals that TPP exhibits localized overlap between its HOMO and LUMO, indicating poor HOMO–LUMO separation efficiency and inferior catalytic activity (Fig. 4b and c). Por-Cage demonstrates excellent HOMO–LUMO separation: the HOMO is predominantly localized at the porphyrin center, while the LUMO extends to the terpyridine groups. This favorable HOMO–LUMO separation significantly promotes intramolecular energy transition, synergistically enhancing photocatalytic oxidation performance (Fig. 4e, f, Tables S5 and S6). Furthermore, using DMA as a fluorescent probe and [Ru(bpy_3_)^2+^] (73%) as a reference, the singlet oxygen yield of the three materials was measured to be 79.12% (Por-Cage), 49.05% (TPP), and 16.61% (L) (Table S2, Fig. S23 and S24).

**Fig. 5:**
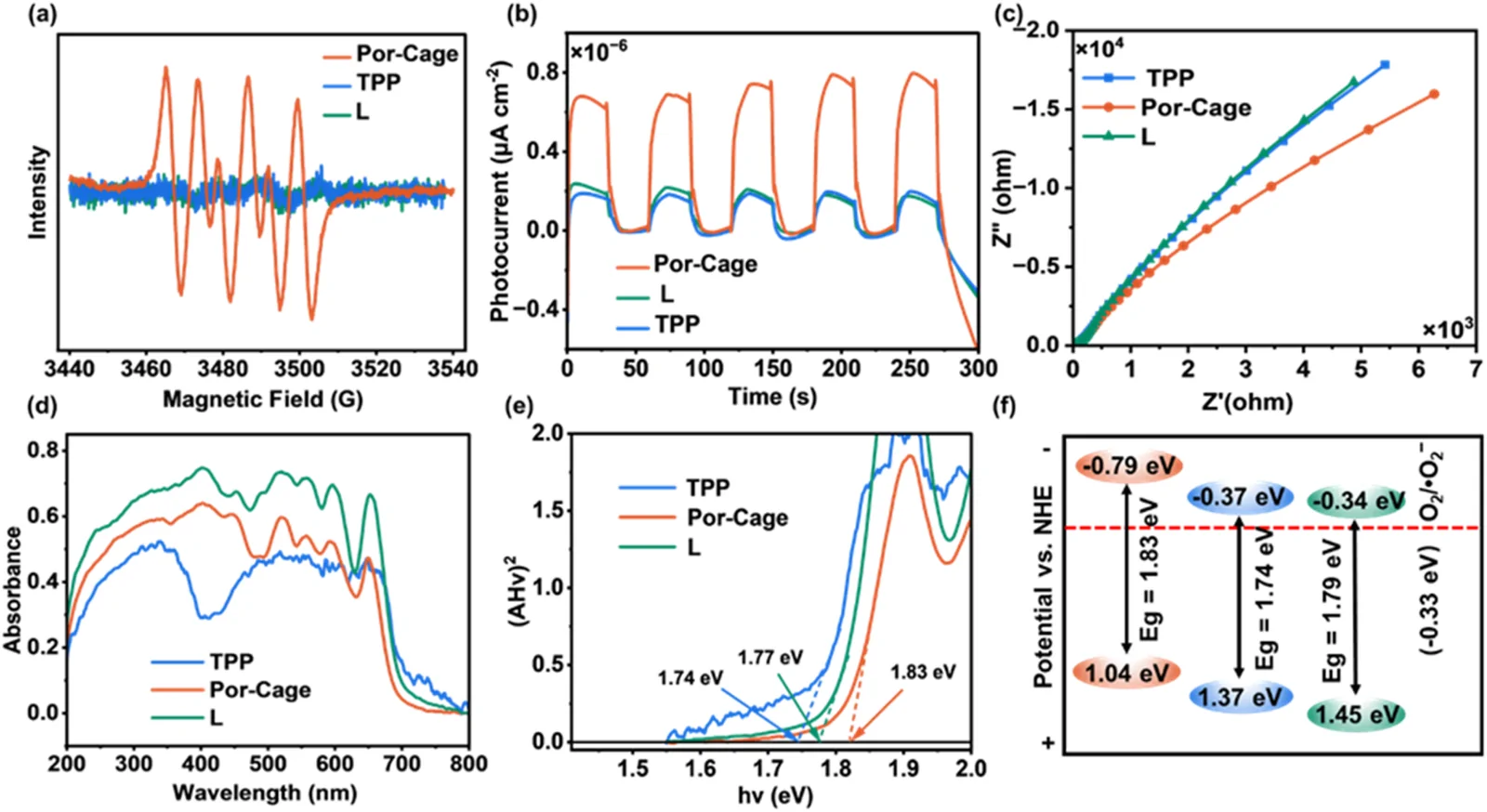
Electrochemical properties of Por-Cage. (a) EPR spectra of Por-Cage, ligand L and TPP mixed with DMPO under visible light irradiation; (b) photocurrent maps of Por-Cage, ligand L and TPP; (c) electrochemical impedance spectra of Por-Cage, ligand L and TPP; (d) DRS of Por-Cage and ligand L and TPP in the solid state; (e) plots of the converted Kubelka–Munk functions of Por-Cage, ligand L, and TPP; (f) schematic diagrams of the energy band structures of Por-Cage, ligand L, and TPP.

When 5,5-dimethyl-1-pyrroline N-oxide (DMPO) was employed as the probe, superoxide radical (·O_2_^−^) formation was detected for Por-Cage, and ·O_2_^−^ signals steadily increased and accumulated over time (Fig. S26). Control experiments using TPP or L revealed no detectable signals (Fig. 5a). Photocurrents and electrochemical impedance spectra were further measured for the three materials. As shown in Fig. 5b, compared to ligand L and TPP, Por-Cage exhibited a stronger photocurrent response under illumination, indicating higher separation efficiency of photo-generated electrons and holes. Corresponding electrochemical impedance spectra also revealed that Por-Cage exhibits a smaller arc radius and lower resistance for charge transfer (Fig. 5c). These results indicate that Por-Cage possesses higher charge separation efficiency, promoting ·O_2_^−^ generation and enhancing photocatalytic efficiency. Solid-state UV diffuse reflectance spectroscopy revealed that Por-Cage, ligand L and TPP all exhibited broad absorption spectra, indicating strong light-harvesting capabilities (Fig. 5d). To elucidate the differences in electronic structures among the three materials, Tauc plots were obtained using the Kubelka–Munk transformation equation. According to the calculations, the bandgaps of the three materials are 1.79 eV (L), 1.74 eV (TPP), and 1.83 eV (Por-Cage). The slightly larger apparent band gap of Por-Cage may arise from coordination-induced electronic modulation and the suppression of aggregation-related low-energy absorption states, rather than from a simple extension of effective π-conjugation. This relatively larger photochemical bandgap endows Por-Cage with higher-energy photogenerated carriers, significantly enhancing their mobility and thereby favoring the driving of redox reactions (Fig. 5e). Analysis of the Mott–Schottky curves demonstrated a positive slope characteristic across all three materials, confirming their typical n-type semiconductor behavior (Fig. S13–S18). The valence-bands were determined from XPS valence band (VB) spectra (Fig. S19–S21), and the conduction bands (CBs) were then calculated using *E*_CB_ = *E*_vB_ − *E*_g_. Accordingly, the CB positions (*vs.* NHE) are −0.34 eV (L), −0.37 eV (TPP), and −0.79 eV (Por-Cage), and the corresponding band alignment is summarized in Fig. 5f. Combined with the bandgap difference, schematic band structures for the three materials were derived. Por-Cage exhibited the most negative conduction band position, which is thermodynamically favorable for electron transfer to O_2_ to form ·O_2_^−^, compared to TPP and L. All these results collectively demonstrate that the unique band structure of Por-Cage provides a theoretical basis for its efficient ·O_2_^−^ generation, highlighting its remarkable potential and high efficiency photocatalytic properties.

### Investigation of the photocatalytic properties of the metallo-organic cage

**Fig. 6:**
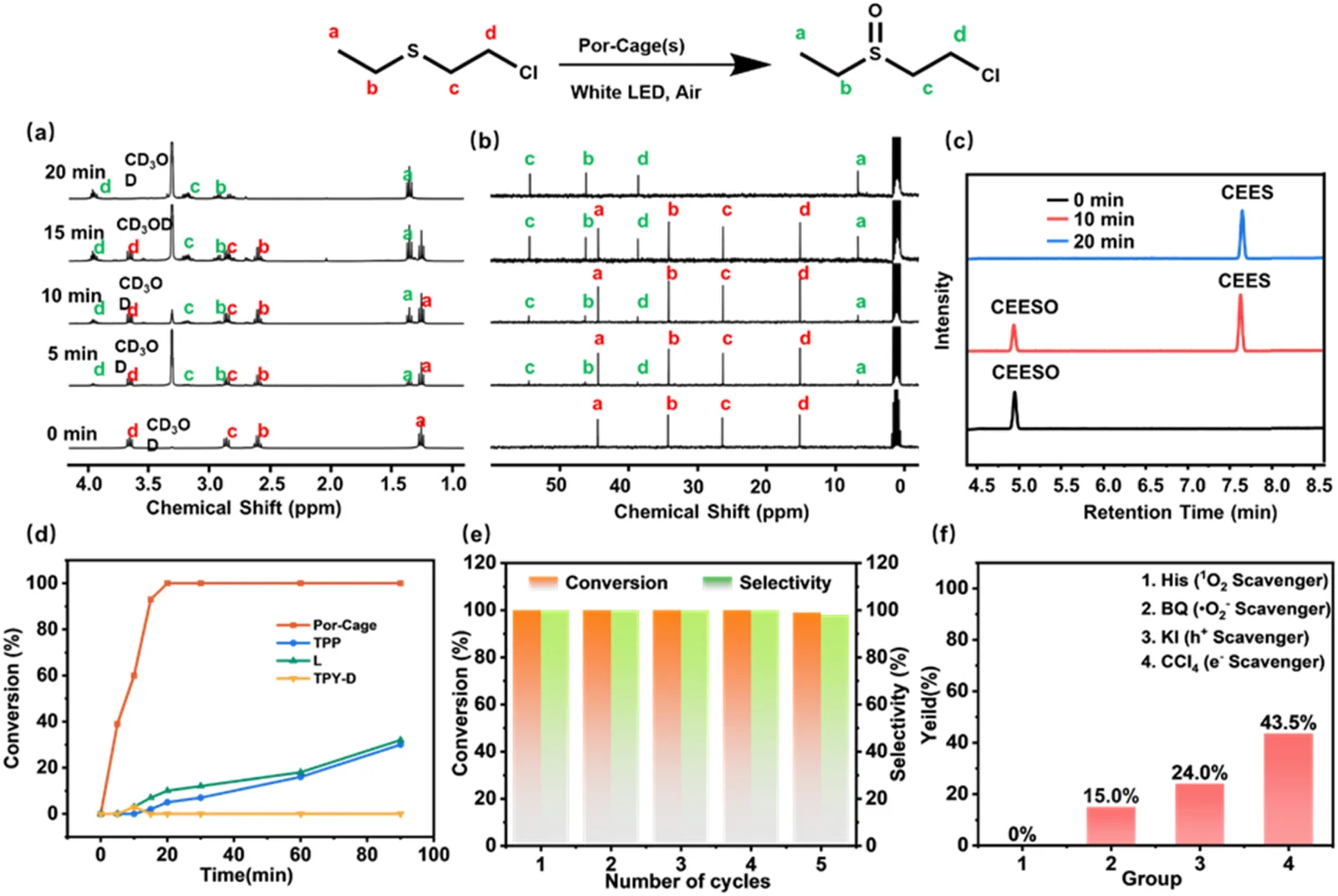
Photocatalytic properties of Por-Cage. (a) ^1^H NMR analysis of CEES photo-oxidation by Por-Cage powder (400 MHz, CD_3_OD, 300 K); (b) ^13^C NMR analysis of CEES photo-oxidation by Por-Cage powder (500 MHz, CD_3_OD, 300 K); (c) GC signals monitoring the progress of the oxidation of CEES to CEESO in the presence of Por-Cage at different times; (d) conversion of CEES in the presence of Por-Cage, ligand L, TPP and TPY-D; (e) reusability of Por-Cage in five consecutive CEES photo-oxidation cycles; (f) yields of the photocatalytic oxidation of thioether to sulfoxide under different scavengers using Por-Cage.

In view of Por-Cage's excellent optoelectronic properties, we selected the photocatalytic degradation of the sulfur-containing mustard gas analogue 2-chloroethyl ethyl sulfoxide (CEES) as a classic process to investigate its performance as a photocatalyst. The heterogeneous photocatalysis of CEES was conducted in CD_3_OD under white LED illumination (100 mW cm^−2^) using three materials (1 mol%, catalyst loading calculated based on the molar amount of porphyrin units in Por-Cage), monitored by NMR spectroscopy (^1^H and ^13^C NMR). To our gratification, the solid powder of Por-Cage achieved selective oxidation of CEES under ambient air conditions (Fig. 6a). It is observed that the detoxification process commences after 5 min of illumination in air, with characteristic proton peaks attributable to CEESO gradually appearing, yielding an initial conversion rate of 39%. As illumination time extended, the oxidation reaction advanced steadily. Complete detoxification was attained at 20 min with a half-life (*t*_1/2_) of 7 min (Fig. 6c and S28). Corresponding ^13^C NMR monitoring results showed four peaks for CEES before irradiation at 44.03 (C^d^), 33.92 (C^c^), 26.06 (C^b^), and 14.88 (C^a^) ppm (blue labels). After 20 min, no detectable CEES peaks remained, while new CEESO peaks appeared at 54.13 (C^c^), 45.99 (C^b^), 38.35 (C^d^), and 6.52 (C^a^) ppm (Fig. 6b). Remarkably, no highly toxic byproduct 2-chloroethyl ethyl sulphone (CEESO_2_) was detected throughout the entire degradation process as illumination time increased. The corresponding gas chromatography-mass spectrometry (GC-MS) results further confirmed that CEES was completely oxidized to CEESO after 20 minutes, with no CEESO_2_ formed (Fig. 6c and S32). Thus, the exceptional photocatalytic performance of Por-Cage facilitates the highly efficient and rapid degradation of the mustard gas simulant CEES under atmospheric conditions with 100% selectivity (Fig. S33–S36).

Furthermore, comparative experiments using ligand L or TPP demonstrated that both materials exhibited weaker catalytic activity in solid powder form (Fig. S31). Even when the reaction time was prolonged to 90 min, the degradation rate remained below 30%. This indicates that the concentration of reactive oxygen species generated by conventional porphyrin molecules is insufficient to initiate effective oxidation reactions, fully corroborating the enhancing effect of MOC structures on catalytic performance. The apparent quantum yield (AQY) of Por-Cage was measured to be 1.27% (385 nm), 1.86% (420 nm), 1.98% (450 nm), 0.91% (485 nm), and 0.63% (520 nm), respectively, suggesting a visible-light-promoted oxidation process (Table S1 and Fig. S22). In addition, to evaluate the intrinsic activity on a per-chromophore basis, the turnover number (TON) and average turnover frequency (TOF) were calculated by normalizing to the molar amount of porphyrin units (three porphyrin units per Por-Cage), giving a TON of 100 and a TOF_avg_ of 303.03 h^−1^ for Por-Cage (20 min, 250 µmol CEES, 100% conversion) (Table S3).

Additional quenching experiments confirmed the mechanism of photo-oxidation reactions, as shown in Fig. 6e. Upon incorporation of histidine (His) as a ^1^O_2_ scavenger, Group 1 was completely inhibited. Similarly, introducing the ·O_2_^−^ scavenger (1,4-benzoquinone, Group 2) significantly impacted yield and achieved only 15% conversion, indicating that ^1^O_2_ and ·O_2_^−^ acted concurrently during photocatalytic oxidation. Under illumination, Por-Cage efficiently initiates the generation and separation of photoexcited electron–hole pairs, with photoexcited electrons rapidly transferring to the Por-Cage surface. The catalytic activity decreased to only 24% upon the addition of KI (hole scavenger) (Fig. 6e, Group 3), demonstrating that photogenerated holes are essential for producing sulfur radical cations *via* single-electron oxidation of thioethers. When CCl_4_ (electron scavenger, Fig. 6e, Group 4) was introduced to trap photogenerated electrons, the efficiency dropped to 43.5%, which confirmed that the electron-derived ·O_2_^−^ participated in the subsequent coupling with sulfur radical cations to form persulfoxide intermediates. The obvious activity suppression by both scavengers collectively proves that the transformation of thioether to sulfoxide relies on the synergistic effect of photogenerated holes and ·O_2_^−^ along the Type I pathway (Fig. S45).

After recovery *via* simple centrifugation and drying, multiple cycling tests confirmed that the recovered Por-Cage retained excellent catalytic performance. After five cycles, the conversion of CEES remained at 99%, with high selectivity maintained (Fig. 6d). The ^1^H NMR and ESI-MS spectra of the recovered catalyst were essentially unchanged compared with those of the fresh Por-Cage, confirming the structural integrity of the cage framework (Fig. S29 and S30). Moreover, inductively coupled plasma-mass spectrometry (ICP-MS) analysis of the reaction solution after photocatalysis revealed negligible Cd^2+^ release (0.631 ppb, about 0.0316% leaching ratio), further demonstrating the stability of the Cd-linked Por-Cage during the photocatalytic process. To further investigate the multifunctionality of Por-Cage in catalytic reactions, we explored the oxidation of various sulfide derivatives (Table 1). The results demonstrate that both sulfides containing electron-withdrawing groups and electron-donating groups, as well as alkyl sulfides, achieved conversion rates exceeding 99% and selectivity approaching 100% under identical photocatalytic oxidation conditions. These remarkable yields and selectivity confirm the broad applicability of Por-Cage as a photocatalyst and highlight its potential for the photocatalytic oxidation of sulfides to sulfoxides (Fig. S37–S44).

**Table 1:** Visible-light-driven aerobic oxidation of sulfide derivatives^a^^,^^b^

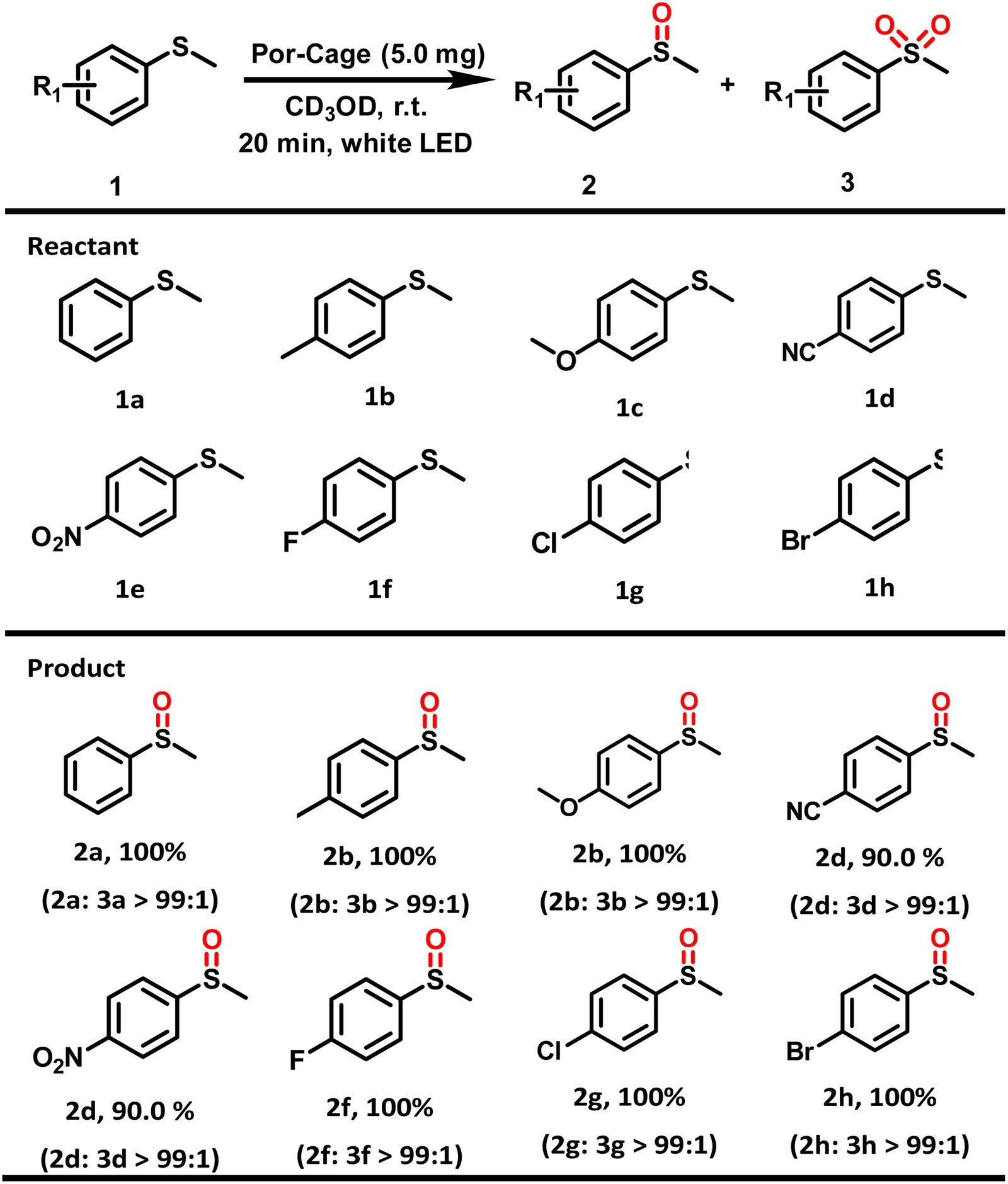

a Reaction conditions: substrate 1 (0.173 mmol), Por-Cage (5.0 mg), CD_3_OD (1.5 mL), r.t., 20 min, white LED.

b Conversion and selectivity were determined by ^1^H NMR analysis after adding the internal standard in the reaction mixtures.

## Conclusions

In summary, we designed and synthesized a porphyrin-centered tetratopic terpyridine ligand. Upon assembly with Cd^2+^ ions, it successfully formed the metallo-organic cage Por-Cage with a truncated trigonal prism structure and *D*_3h_ symmetry. Following structural characterization and validation, we comprehensively investigated the photophysical and optoelectronic properties of Por-Cage and two controls: TPP and ligand L. Our research reveals that Por-Cage exhibits exceptional reactive oxygen species generation capabilities, attributable to two synergistic mechanisms: the spatial confinement effect of the porphyrin unit and enhanced inter-system crossing ability significantly boost ^1^O_2_ yield; while its n-type semiconductor properties in the solid state and enhanced conjugated structure improve light harvesting and charge separation efficiency, facilitating ·O_2_^−^ production. Upon illumination, Por-Cage demonstrated highly selective and efficient oxidation of sulfide pollutants under ambient air conditions. Heterogeneous CEES detoxification under ambient air conditions can achieve up to 100% selectivity, fast reaction rate (*t*_1/2_ = 7 min), excellent catalyst reusability and substrate compatibility. Compared to conventional methods, this photosensitizer operates under ambient air conditions, offering distinct advantages in terms of being green, eco-friendly, and cost-effective. The findings also have significant implications for advancing the application of photocatalytic materials in environmental remediation.

## Author contributions

Z. Z., P. W. and T. W. conceived the project. G. C. and Q. B. performed the synthesis and characterization experiments. H. C. performed the MS measurements. T. L. performed the PL measurements. X. L. and C. Z. performed the photoelectrochemical performance studies. Z. Z. analyzed and organized the data. W. Z. performed the transient absorption spectroscopy measurements. Y. G. performed structural simulation calculations for the MOC. M. W. performed the DRS tests. Q. B. and G. C. drafted the manuscript. T. W., Z. Z. and P. W. revised and oversaw the project. All authors discussed the results and commented on the paper.

## Conflicts of interest

The authors declare that they have no known competing financial interests or personal relationships that could have appeared to influence the work reported in this paper.

## Supplementary Material

SC-017-D6SC03415B-s001

SC-017-D6SC03415B-s002

## Data Availability

CCDC 2494623 contains the supplementary crystallographic data for this paper.^63^ Further analytical data are reported in the supplementary information (SI) to this article. Data are available upon request from the authors. Supplementary information is available. See DOI: https://doi.org/10.1039/d6sc03415b.
