# Supplementary material for: Porphyrin-based metallo-organic cage for selective photocatalysis under heterogeneous and atmospheric conditions
Source: Chem Sci. 2026 Jul 17;17(36):17359–69. doi: 10.1039/d6sc03415b (PMC13418410; doi:10.1039/d6sc03415b)
Supplement: Supplementary file 1 [file SC-017-D6SC03415B-s001.pdf]

Supplementary Information (SI) for Chemical Science.
This journal is © The Royal Society of Chemistry 2026

Porphyrin-Based Metallo-Organic Cage for Selective Photocatalysis Under Heterogeneous and Atmosphere Condition

Qixia Bai[1,2,§], Gang Chen[3,§], Tun Wu[1], Wei Zhang[4], Huoqing Chen[1], Tian Li[1], Xiang Li[1], Yu-Ming Guan[1], Zhihong Chen[1], Ming Wang[5]*, Pingshan Wang[1]*, Zhe Zhang[1]**

[1] Key Laboratory for Water Quality and Conservation of the Pearl River Delta (Ministry of Education), Institute of Environmental Research at Greater Bay Area, Guangzhou University, Guangzhou 510006, China

[2] College of Materials Science and Engineering, Shanxi Normal University, Taiyuan, 030000, China

[3] School of Chemistry and Chemical Engineering, Guangdong Provincial Key Laboratory of Optoelectronic Materials and Sensor Components, Guangzhou Key Laboratory of Sensing Materials & Devices, Centre for Advanced Analytical Science, Guangzhou University, Guangzhou, 510006, China

[4] School of Physics and Materials Science, Guangzhou University, Guangzhou 510006, China

[5] State Key Laboratory of Supramolecular Structure and Materials, College of Chemistry, Jilin University, Changchun, 130012, China

Corresponding author e-mail: chemwt@gzhu.edu.cn; chemwps@gzhu.edu.cn; mingwang358@jlu.edu.cn; zhezhang2018@gzhu.edu.cn

Table of Content

1. Synthetic route of ligand L 3

2. Experimental section 4

3. Synthesis of the compounds and supramolecules 8

4. ESI-MS spectra data of MOCs (NTf_2^- as counterion) 11

5. ^{1}H NMR, ^{13}C NMR, 2D COSY NMR, 2D NOESY NMR, 2D DOSY NMR ..12

6. Investigation of photoelectric properties of Por-Cage.....................................17

7. Investigation of the Photocatalytic Properties of the Metallo-Organic Cage Por-Cage..27

8. X-ray crystallographic data and structures 37

9. TD-DFT Calculations 40

10. References 42

1. Synthetic route of ligand L

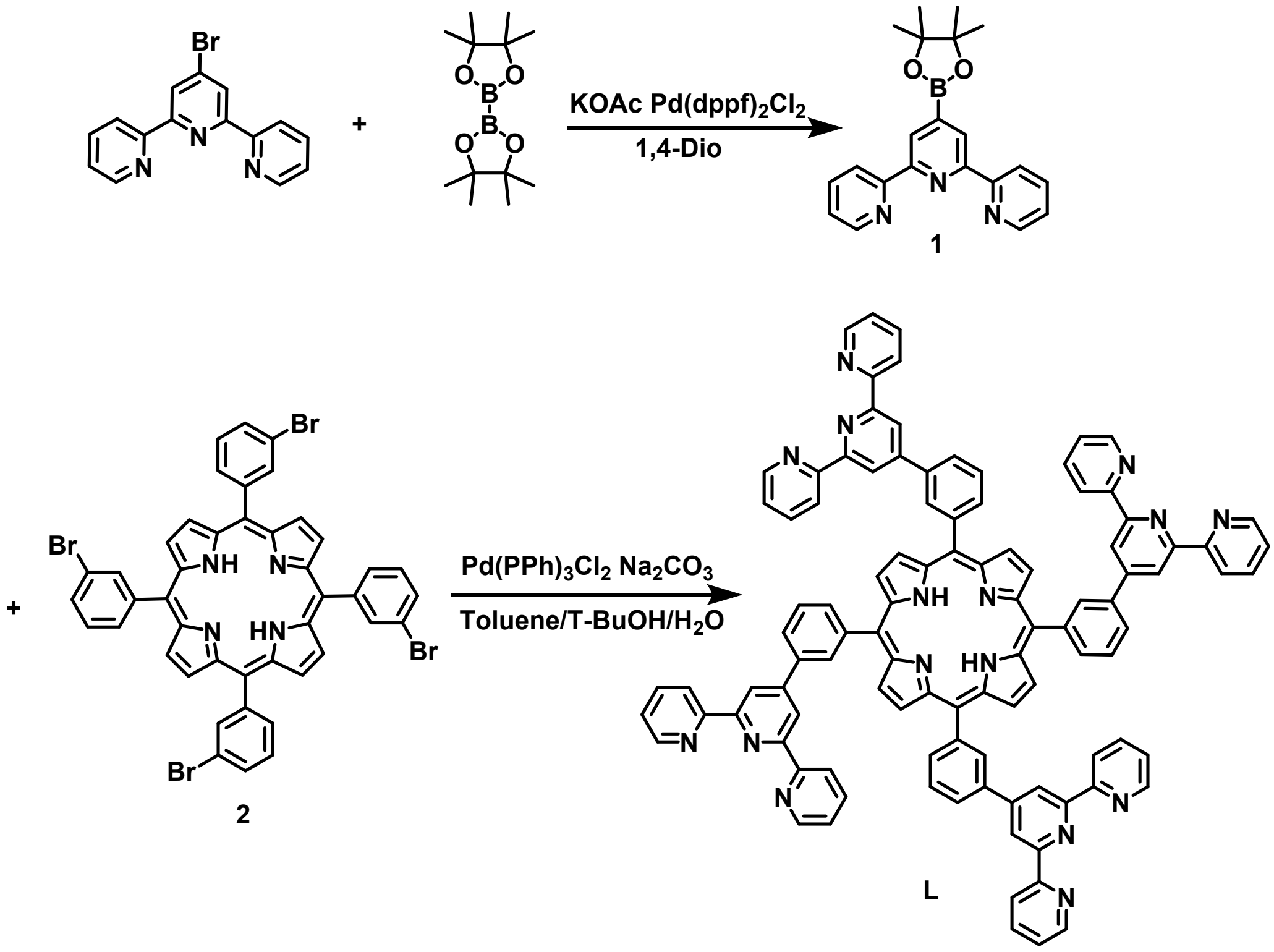

Scheme S1. Synthesis of ligand **L.**

2. Experimental section

General procedures. Chemicals were purchased from Sigma/Aldrich, Energy Chemical, Bidepharm and used without further purification. Thin-layer chromatography (TLC) was conducted on flexible sheets (Baker-flex) precoated with Al_2O_3 (IB-F) or SiO_2 (IB2-F). Column chromatography was conducted using basic Al_2O_3 Brockman Activity I (60-325 mesh) or SiO_2 (60-200 mesh) from Fisher Scientific. NMR spectra were recorded on Bruker NMR 400 or 500 MHz spectrometers, using $CDCl_3$ for ligand, CD_3CN for MOC. ESI mass spectrometry (MS) experiments were performed on a Waters Synapt HDMS G2-Si quadrupole/time-of-flight (Q/TOF) tandem mass spectrometer. This instrument contains a triwave device between the Q and TOF analyzers, consisting of three collision cells in the order trap cell, ion mobility cell, and transfer cell. Trap and transfer cells are pressurized with Ar, and the ion mobility cell is pressurized with N_2 flowing in a direction opposite to that of the entering ions.

Mass Spectrometry and Ion Mobility. ESI-MS and TWIM-MS were recorded with a Waters Synapt G2-Si tandem mass spectrometer, using solutions of 0.01 mg sample in 1 mL of $CHCl_3/CH_3OH$ (1:3, v/v) for ligand or 0.5 mg sample in 1 mL of CH_3CN/CH_3OH (3:1, v/v) for complex. All samples were infused into the ESI source at a flow rate of 6 μL/min by a syringe pump (KDS-100, KD Scientific). The TWIM-MS experiments were performed under the following conditions: ESI capillary voltage, 2 kV; sample cone voltage, 35 V; source offset, 42 V; source temperature 150 ºC; desolvation temperature, 250 ºC; cone gas flow, 10 L/h; desolvation gas flow, 700 L/h (N_2); source gas control, 0 mL/min; trap gas control, 3 mL/min; helium cell gas control, 120 mL/min; ion mobility (IM) cell gas control, 30 mL/min; sample flow rate, 8 μL/min; IM traveling wave height, 25 V; and IM traveling wave velocity, 1200 m/s. Data were collected and analyzed by using Mass Lynx 4.2 and Drift Scope 2.9.

Molecular Modeling. Energy minimization of the macrocycles was conducted with the Materials Studio version 6.0 program, using the Anneal and Geometry Optimization tasks in the Forcite module (Accelrys Software, Inc.). The counterions

were omitted.

UV-vis absorption, fluorescence properties. UV-vis absorption spectra were recorded on Thermo Fisher Scientific Evolution 201 spectrophotometer at room temperature (10^{-6} M in $CHCl_3$ or CH_3CN) and were corrected with the background spectrum of the solvent. Fluorescence properties were performed on Edinburgh-FS5 Fluorescence spectrometer at 298 K (10^{-6} M in $CHCl_3$ or CH_3CN).

UV-Vis DRS. UV-3600 UV-Vis Diffuse Reflectance Spectrometer was used to evaluate the wavelength range of light absorption of the materials, with $BaSO_4$ as the reference material, and the scanning wavelength was 200-800 nm at room temperature.

EPR. EPR spectra were measured using a Bruker A300 electron paramagnetic resonance spectrometer with 4-oxo-TEMPO or 5,5-dimethyl-1-pyrroline-N-oxide as a free radical trapping agent added to the material dispersion, and EPR spectra were obtained after irradiation in darkness and visible light for 5 min. The test instrument settings were modulation frequency: 100.00 KHz; modulation amplitude: 2.00 G; sweep width: 100.00 G; time constant: 40.960 ms; transition: 40.000 ms; scan time: 60.7 s; microwave power 20.00 mW; and frequency 9.84 GHz.

Photoelectrochemical Performance. Mott-Schottky photoelectrochemical measurements were performed using a CHI 760D electrochemical workstation. A standard three-electrode system was constructed using 0.1 M sodium sulfate (Na_2SO_4) solution as the electrolyte, with platinum (Pt) as the counter electrode, silver/silver chloride (Ag/AgCl) as the reference electrode, and fluorine-doped tin oxide (FTO) coated with the sample as the working electrode. The electrolyte was bubbled with high-purity nitrogen for 15 minutes prior to testing to eliminate dissolved oxygen interference and ensure testing accuracy. The working electrode preparation procedure is as follows: 5 mg of ground supramolecular material is uniformly dispersed in a mixture of deionized water and Nafion membrane solution. After 30 minutes of ultrasonic treatment, the dispersion is precisely pipetted onto the FTO glass electrode surface and left to dry naturally overnight in a fume hood. Mott-Schottky curve measurements were conducted within the potential range of 0.2–1.2 V (vs. Ag/AgCl), with data acquired at three frequencies: 500 Hz, 800 Hz, and 1000 Hz. Photocurrent

measurements were performed under 0 V illumination with invisible light ($\lambda > 420$ nm). The electrochemical impedance curve was measured at a voltage of 0.2 V (vs. Ag/AgCl reference electrode) using a three-electrode configuration, with a frequency range from 10 kHz to 0.1 Hz.

XPS. The XPS spectra were measured using a Thermo Scientific K-Alpha X-ray photoelectron spectrometer. Setting parameters: 400 μm spot size, 12 kV operating voltage, 6 mA filament current; 150 eV full-spectrum scanning fluence in 1 eV steps; 50 eV narrow-spectrum scanning fluence in 0.1 eV steps.

TA measurement. Transient absorption spectroscopy measurements were performed using a custom-built measurement system. This system was driven by a commercial femtosecond (fs) laser operating at a repetition rate of 1 kHz, with a pulse duration of ~170 fs and a wavelength of 800 nm. The amplifier (Legend EliteF 1K HE + II, Coherent, California, USA) was seeded by an oscillator (Mira-HP, Coherent, California, USA) operating at 80 MHz. The fundamental laser beam was split into two beams: one beam was frequency-doubled (400 nm) or directly used as pump light to excite organic photovoltaic (OPV) samples. The other beam serves as the detection beam for differential absorption measurements, focused onto a nonlinear crystal to generate supercontinuum white light. The detection beam is guided to a monochromator (Omni-λ200i, Zolix, Beijing, China) and detected by a CCD detector (Paschen Instruments, Lund, Sweden). The time delay between the pump and probe beams is controlled by a mechanical delay line. For TA measurements, all samples are mounted in a nitrogen-filled optical chamber.

Single crystal X-ray diffraction. Single crystals of **Por-Cage** suitable for X-ray diffraction were obtained by crystallization from CH_3CN/Isopropyl ether (6 mg/mL) at 15 °C. Single-crystals X-ray diffraction data for **Por-Cage** was collected on a Bruker D8 VENTURE diffractometer using a mirror monochromated Ga-Kα radiation. Using Olex2 1.5, the structures were solved with the SIR2004 [2] structure solution program using Direct Methods and refined with the XH [3] refinement package using CGLS minimisation. Data refinement and reduction were undertaken with Bruker SAINT. The structures were solved by direct methods and refined by full-matrix least-squares on F2

with anisotropic displacement using the SHELXTL-97 software package. Details on crystals data collection and refinement were summarized in Table S1. CCDC: 2494623.

DFT Calculations. All calculations were carried out with the Gaussian 16 A 03 software. The ωB7XD[1] functional was adopted for all calculations For geometry optimization and frequency calculations, the def2-SVP[2, 3] basis set was used for all atoms. The vertical excitation energies of the first 40 singlet/triplet excited states were calculated at the TD-ωB7XD/def2-SVP calculation level using time-dependent density functional theory. All wave function analyses were finished via the Multiwfn 3.8(dev)[3, 4] code. The isosurface maps were rendered by means of the VMD 1.9.3[6] visualization program based on the files exported by Multiwfn.

3. Synthesis of the compounds and supramolecules

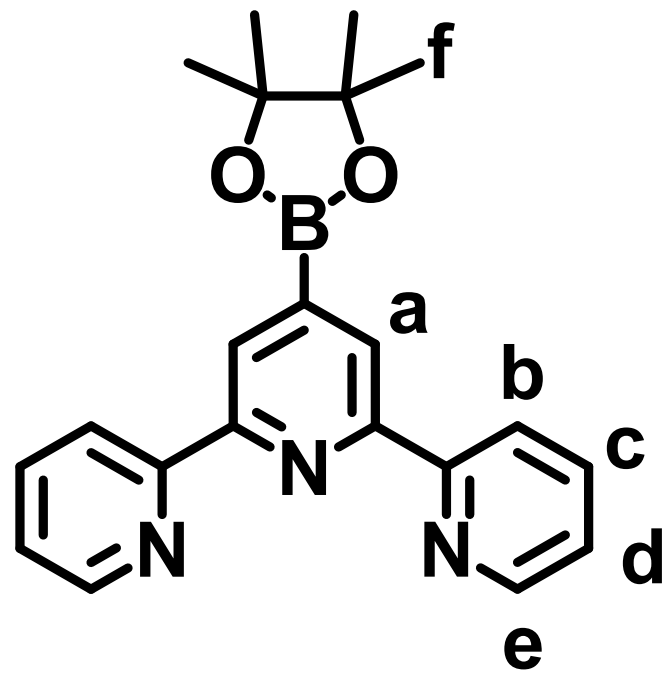

Compound **1**: Precisely weigh 4'-bromo-2,2':6',2"-terpyridine (1 g, 3.2 mmol), pinacol boronic acid ester (1.22 g, 4.8 mmol), potassium acetate (942.14 mg, 9.6 mmol), and $Pd(dppf)_2Cl_2$ (117 mg, 0.16 mmol) were weighed precisely and added to a 200 mL three-neck flask. Subsequently, 50 mL of anhydrous 1,4-dioxane was drawn into the mixture using a syringe. The entire reaction system was evacuated and purged with nitrogen gas three times to ensure a nitrogen atmosphere throughout. The reaction proceeded at 85 °C for 12 h. After reaction completion, the mixture was cooled to room temperature, dried under vacuum, and 100 mL of dichloromethane was added to the solid. Following sonication, the dichloromethane-insoluble solid was filtered off, and the filtrate was collected. Vacuum drying yielded 900 mg of purple oily compound **1** (78%). ^{1}H NMR (500 MHz, $CDCl_3$, 300 K) δ 8.80 (s, 2H, *Ph-H^a*), 8.74 – 8.71 (d, *J* = 15.0 Hz, 2H, *Ph-H^b*), 8.61 – 8.58 (d, *J* = 15.0 Hz, 2H, *Ph-H^c*), 7.88 – 7.83 (t, *J* = 12.5 Hz, 2H, *Ph-H^d*), 7.35 – 7.30 (m, 2H, *Ph-H^e*), 1.38 (s, 13H, *-CH_3-H^f*). ^{13}C NMR (100 MHz, $CDCl_3$) δ 156.49, 154.76, 149.15, 136.76, 126.27, 123.60, 121.22, 84.48, 24.95. ESI-MS (*m/z*): Calcd. for $[C_{21}H_{22}BN_3O_2+H]^+$: 360.22. Found: 360.22.

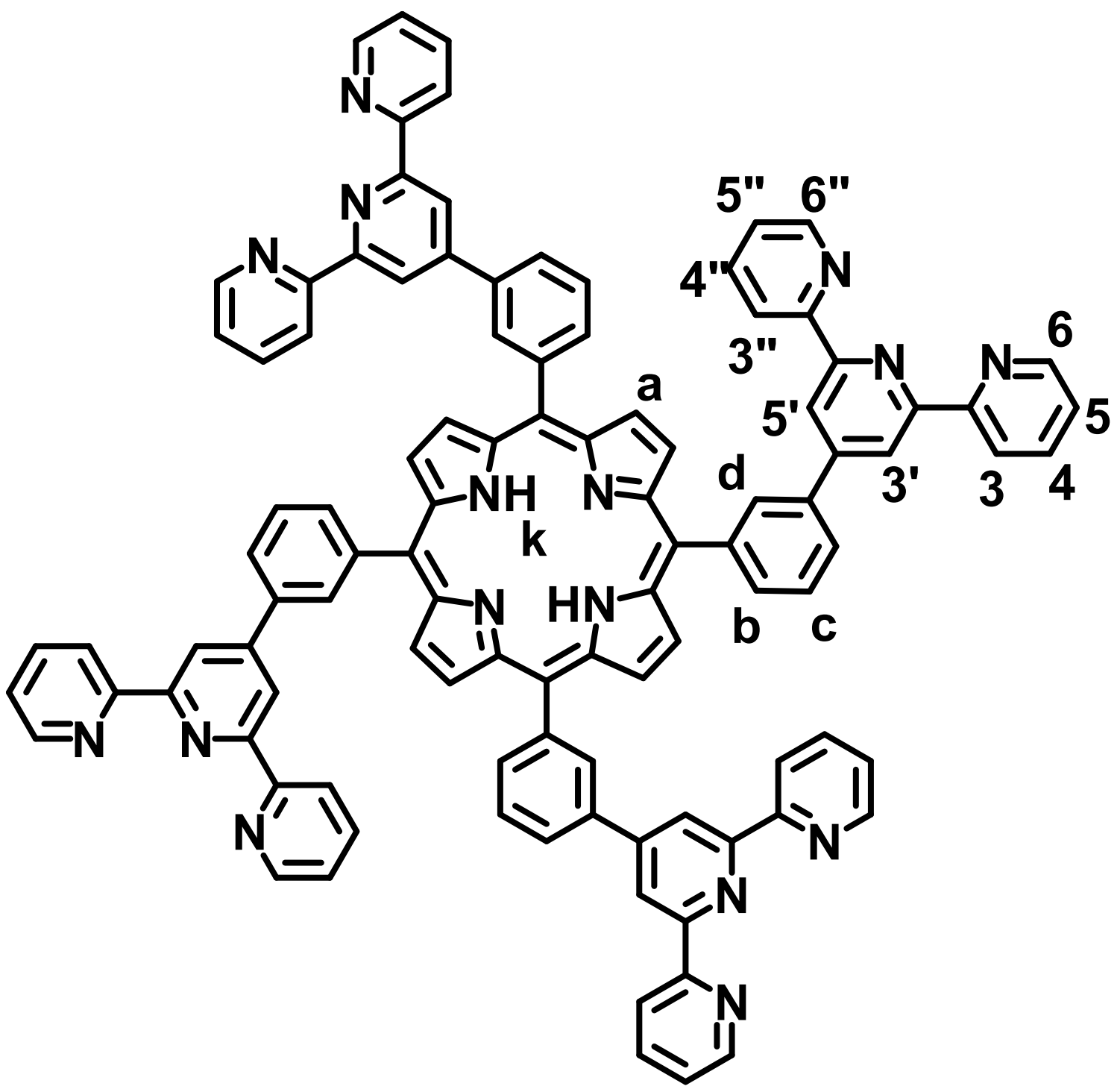

Ligand L: Add compound 2 (466.10 mg, 0.50 mmol), compound 1 (900.00 mg, 2.51 mmol), $Pd(PPh_2)_2Cl_2$ (70.10 mg, 0.10 mmol), and Na_2CO_3 (636.00 mg, 6.00 mmol) into a 250 mL three-neck flask. Under nitrogen atmosphere, add 60 mL toluene, 36 mL water, and 24 mL tert-butanol using a syringe. After sonication to ensure complete dispersion of reactants, evacuate and replace with nitrogen gas three times to maintain an inert atmosphere throughout the reaction. Stir at 85 °C for 4 days. After reaction completion, allow the mixture to cool to room temperature. Transfer to a separatory funnel, add CH_2Cl_2 to extract the mixture. Combine the organic layers, wash with brine, and dry over anhydrous $MgSO_4$ to remove excess moisture. Vacuum concentrate the residue. Purify the residue by rapid column chromatography (SiO_2) and elute with CH_2Cl_2:CH_3OH (100 : 2, v/v) as the eluent, yielding 600 mg of purple solid (78% yield). ^{1}H NMR (400 MHz, $CDCl_3$, 300 K) δ 9.00 – 8.90 (t, *J* = 25.0 Hz,16H, *Tpy-H*$^{3',5'}$, *Por-H*^{a}), 8.81 (s, 4H, *Ph-H*^{d}), 8.67 – 8.54 (m, 16H, *Tpy-H*$^{3,3''}$, *Tpy-H*$^{6,6''}$), 8.39 – 8.30 (d, *J* = 36.0 Hz, 8H, *Ph-H*^{b},*Ph-H*^{e}), 7.96 – 7.89 (m, 4H, *Ph-H*^{c}), 7.87 – 7.78 (m, 8H, *Tpy-H*$^{4,4''}$), 7.33 – 7.27 (m, 4H, *Tpy-H*^{5}), 7.26 – 7.20 (m, 4H, *Tpy-H*$^{5''}$), -2.56 – -2.72 (s, 2H, *Por-NH*^{k}). ^{13}C NMR (126 MHz, $CDCl_3$) δ 155.15, 155.09, 155.07, 149.22, 149.18, 148.07, 141.94, 141.88, 135.75, 134.09, 132.19, 132.15, 126.33, 125.77, 122.76, 122.70, 122.65, 120.30, 120.21, 120.13, 118.76, 118.32. ESI-TOF (*m/z*): Calcd. for

$[C_{104}H_{66}N_{16} + H]^+$: 1549.78, found: 1540.78; $[C_{104}H_{66}N_{16} + 2H]^{2+}$:770.89, found: 770.80.

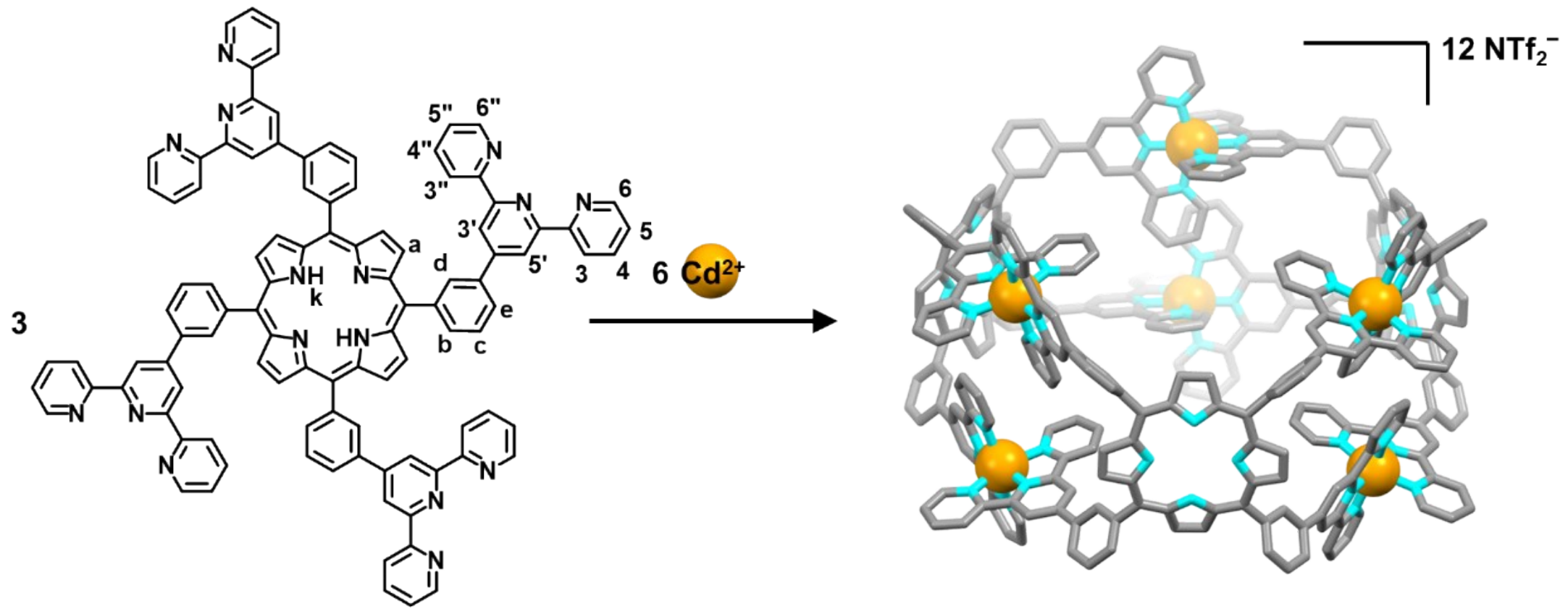

Por-Cage: First, precisely weighted ligand L (10.00 mg, 6.49 μmol) into a 30 mL glass vial, then slowly add 10 mL of $CHCl_3$ and sonicate until completely dissolved. Next, add dropwise a CH_3OH solution (1 mg/mL) containing $Cd(NO_3)_2$•$4H_2O$ (4.00 mg, 12.98 μmol), followed by 10 mL of methanol. Stir the mixture at 60 °C for 8 hours. After the reaction, heating was stopped. Upon cooling to room temperature, an excess of $LiNTf_2$ was added until a distinct precipitate formed. The mixture was transferred to a centrifuge tube and centrifuged. The supernatant was removed, and the residue was washed three times with a mixture of distilled water and methanol. The washed residue was dried in an oven, yielding 13.72 mg of purple solid (98% yield). ^{1}H NMR (500 MHz, CD_3CN, 300 K) δ 9.10 – 9.05 (d, J = 20.0 Hz, 8H, *Por-H*^{a}), 8.97 (s, 8H, *Tpy-H*$^{3',5'}$), 8.75 – 8.72 (d, J = 15.0 Hz, 4H, *Ph-H*^{b}), 8.70 (s, 4H, *Ph-H*^{e}), 8.56 – 8.50 (t, J = 12.0 Hz, 12H, *Tpy-H*$^{3,3''}$, *Ph-H*^{d}), 8.26 – 8.21 (t, J = 12.5 Hz, 4H, *Ph-H*^{c})), 7.90 – 7.85 (d, J = 25.0 Hz, 8H, *Tpy-H*$^{6,6''}$), 7.74 – 7.69 (t, J = 12.5 Hz, 8H, *Tpy-H*$^{4,4''}$), 6.99 – 6.94 (m, 8H, *Tpy-H*$^{5,5''}$), -2.80 (s, 2H, *Por-NH*^{k}). ESI-TOF (*m/z*): 2605.01$[M-3NTf_2^-]^{3+}$ (calcd *m/z*: 2605.01), 1883.73$[M-4NTf_2^-]^{4+}$ (calcd *m/z*: 1883.73), 1450.95$[M-5NTf_2^-]^{5+}$ (calcd *m/z*: 1450.95), 1162.43 $[M-6NTf_2^-]^{6+}$ (calcd *m/z*: 1162.44), 956.35 $[M-7NTf_2^-]^{7+}$ (calcd *m/z*: 956.35), 801.79 $[M-8NTf_2^-]^{8+}$ (calcd *m/z*: 801.79), 681.58 $[M-9NTf_2^-]^{9+}$ (calcd *m/z*: 681.58).

4. ESI-MS spectra data of MOCs (NTf_2^- as counterion)

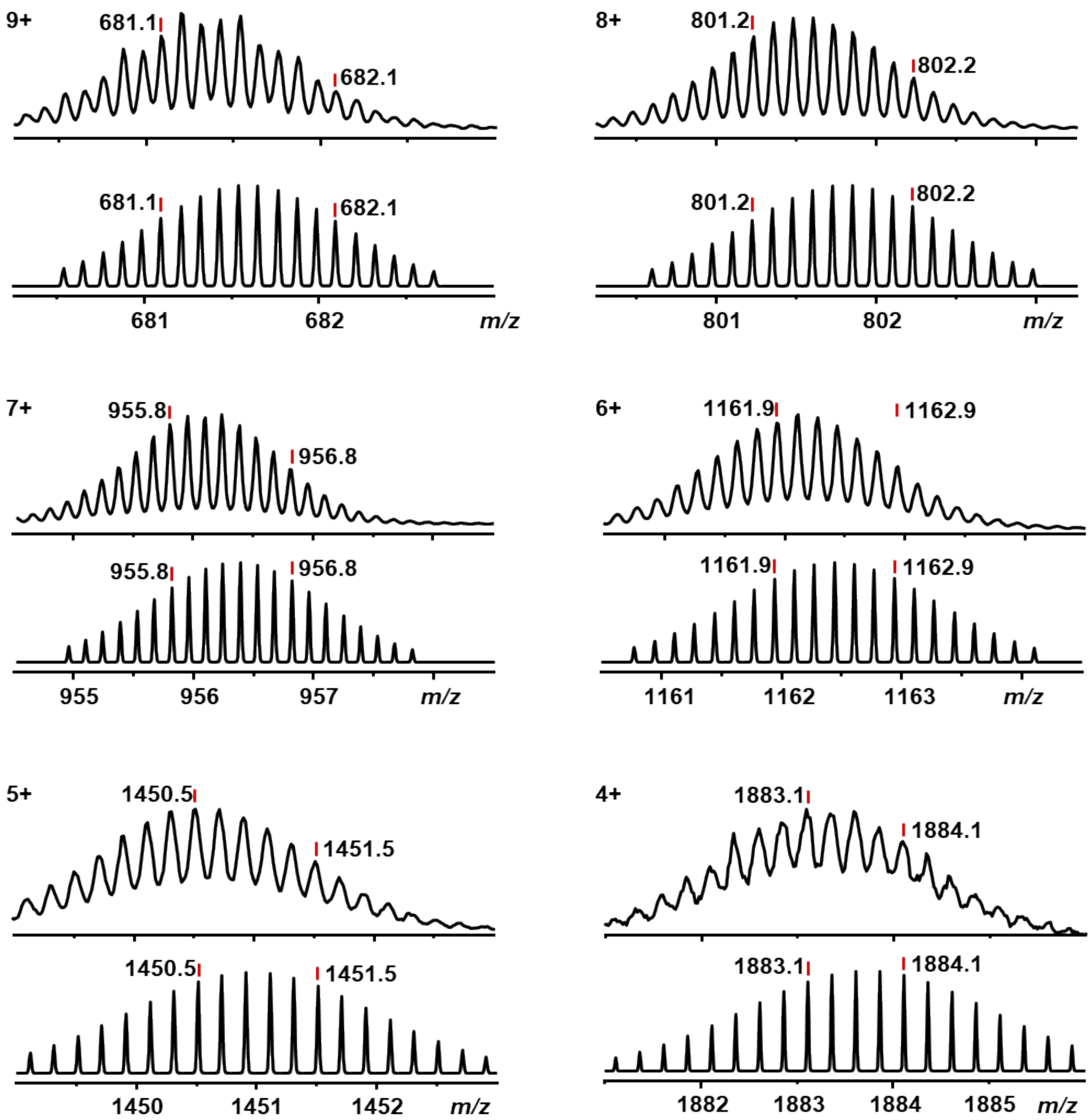

Figure S1. Measured (top) and calculated (bottom) isotope patterns for different charge states observed from **Por-Cage** (NTf_2^- as counterion).

5. ^{1}H NMR, ^{13}C NMR, 2D COSY NMR, 2D NOESY NMR, 2D DOSY NMR

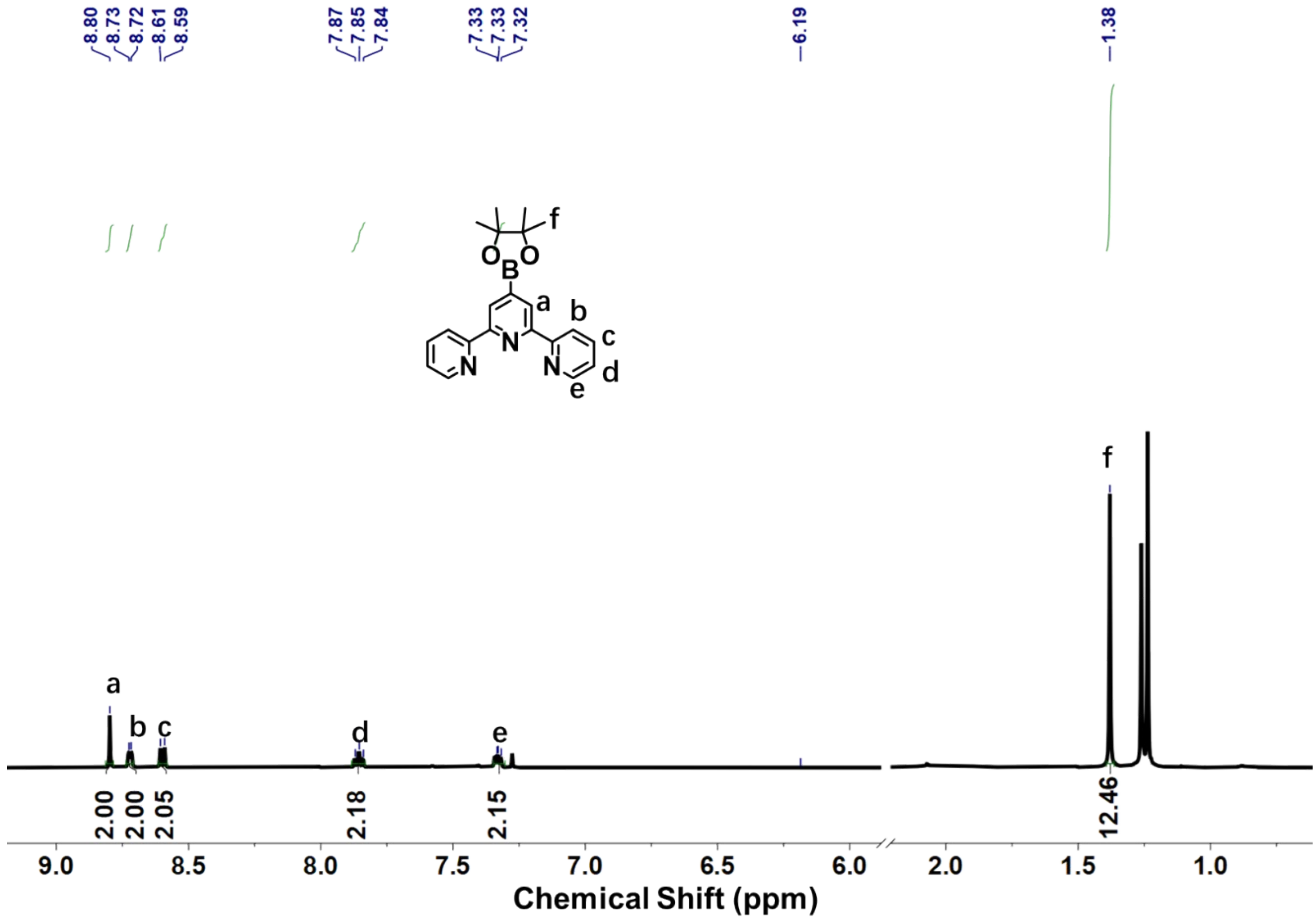

Figure S2. ^{1}H NMR (400 MHz, $CDCl_3$, 300 K) spectrum of compound **1.**

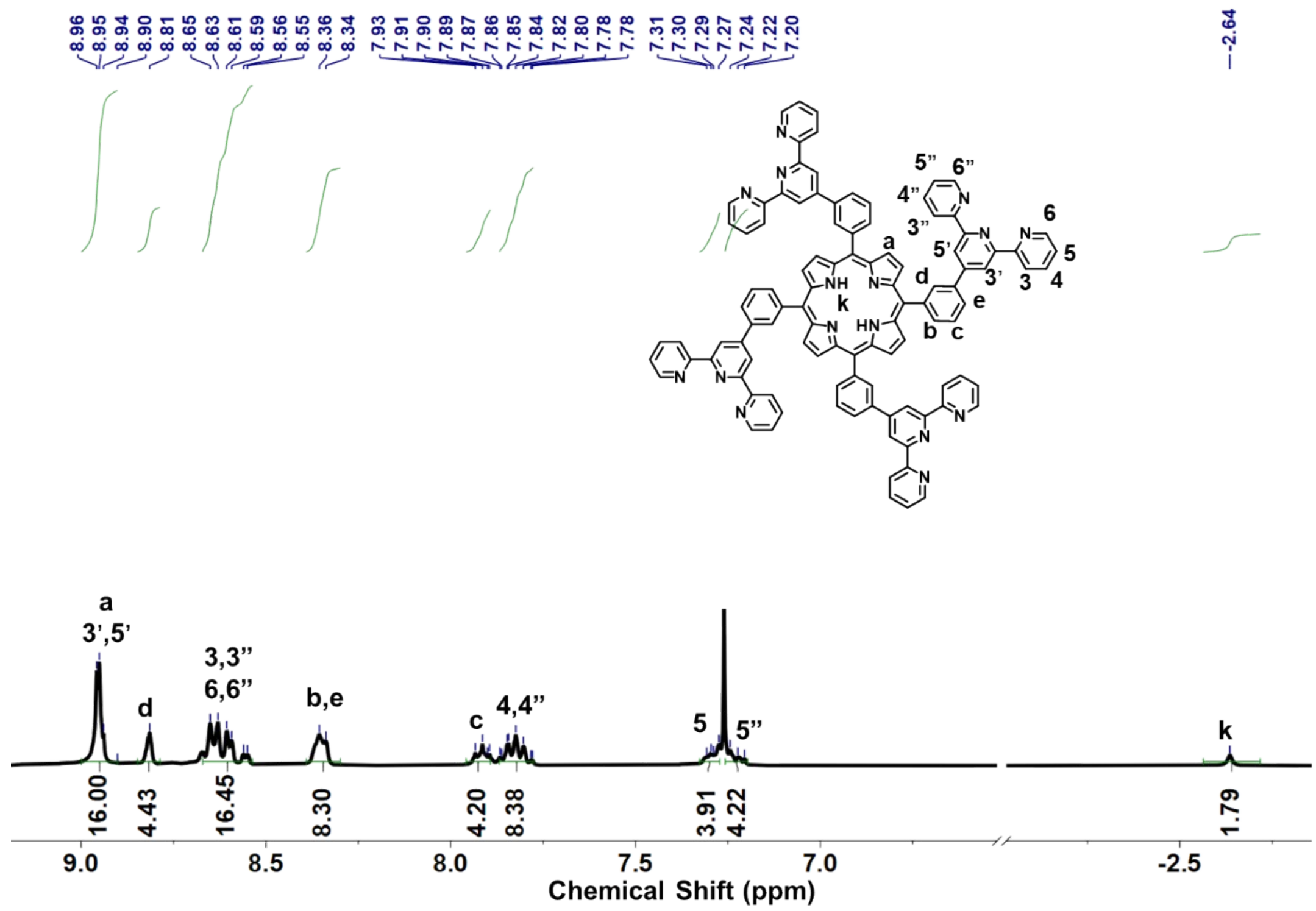

Figure S3. ^{1}H NMR (400 MHz, $CDCl_3$, 300 K) spectrum of ligand **L.**

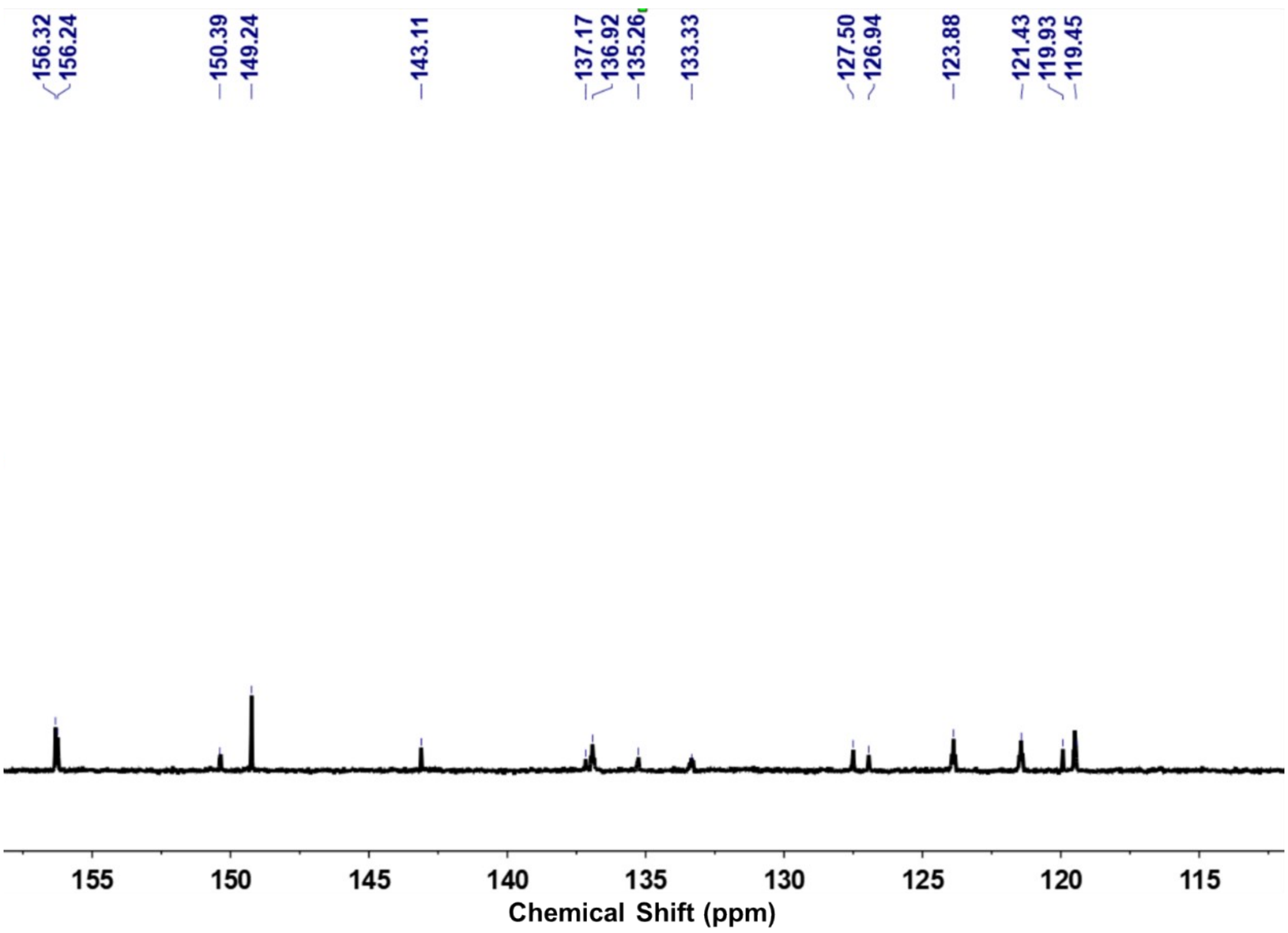

Figure S4. ^{13}C NMR (101 MHz, $CDCl_3$, 300 K) spectrum of ligand **L.**

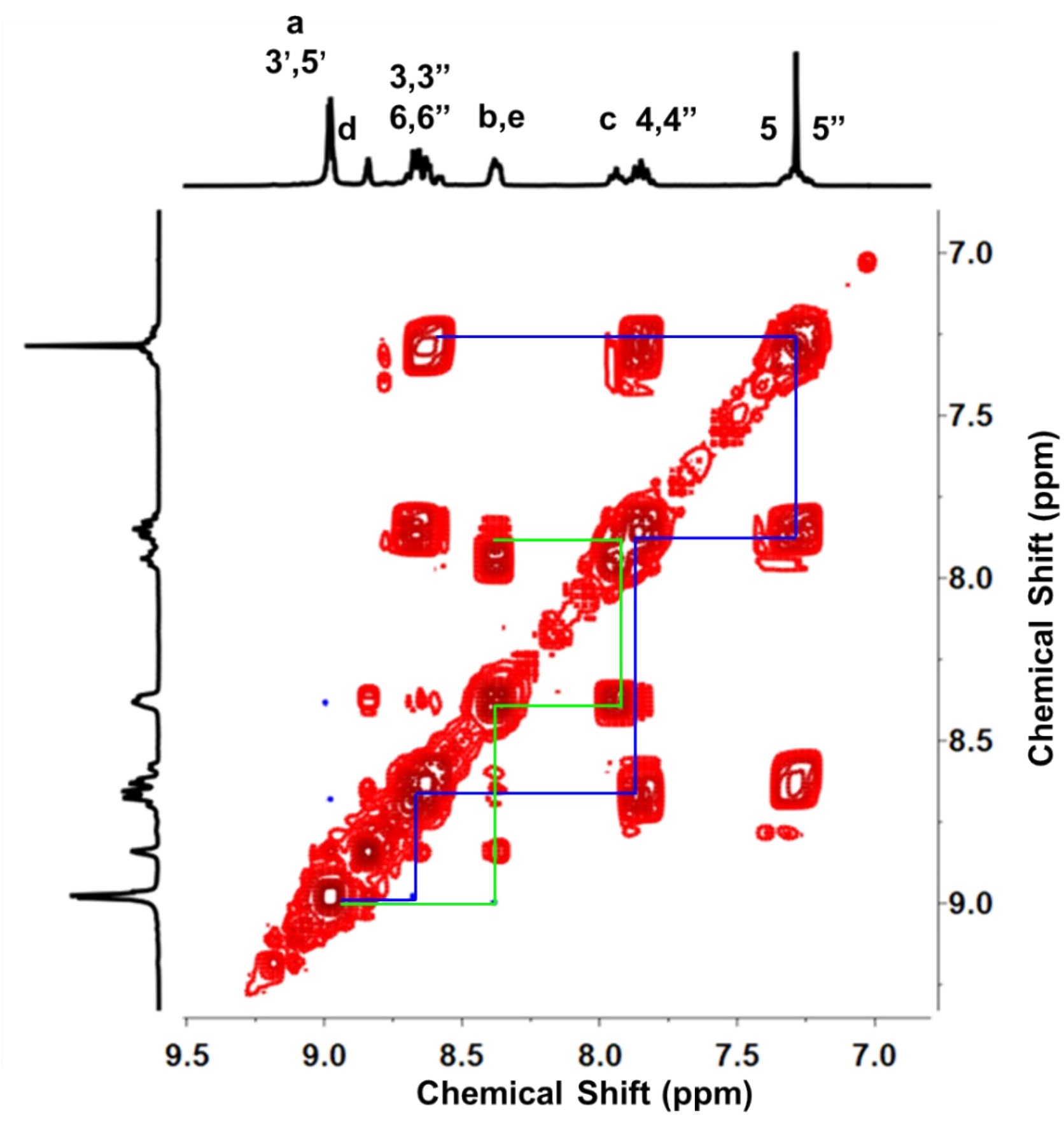

Figure S5. 2D COSY NMR (400 MHz, $CDCl_3$, 300 K) spectrum of ligand **L.**

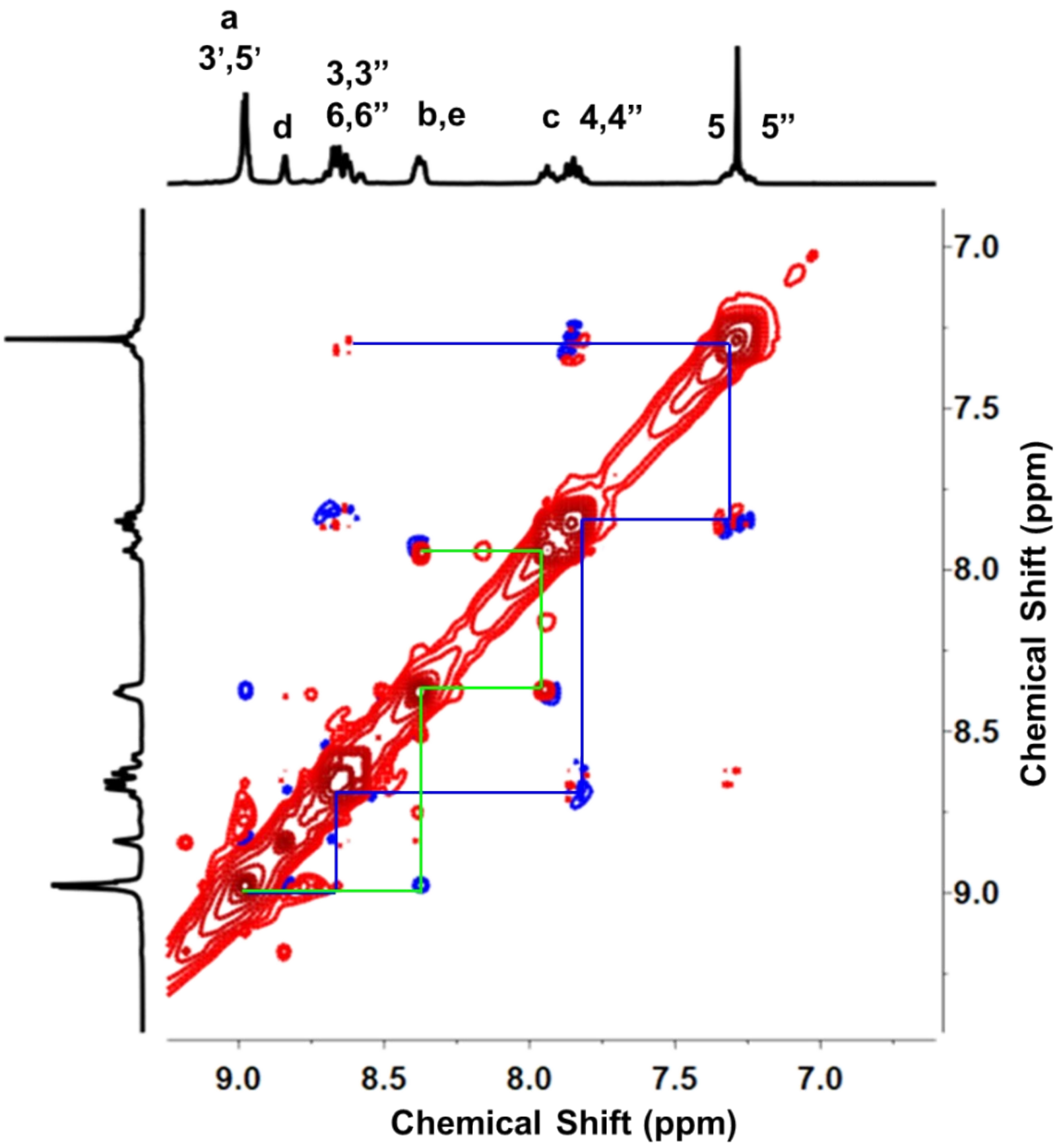

Figure S6. 2D NOESY NMR (400 MHz, $CDCl_3$, 300 K) spectrum of ligand **L.**

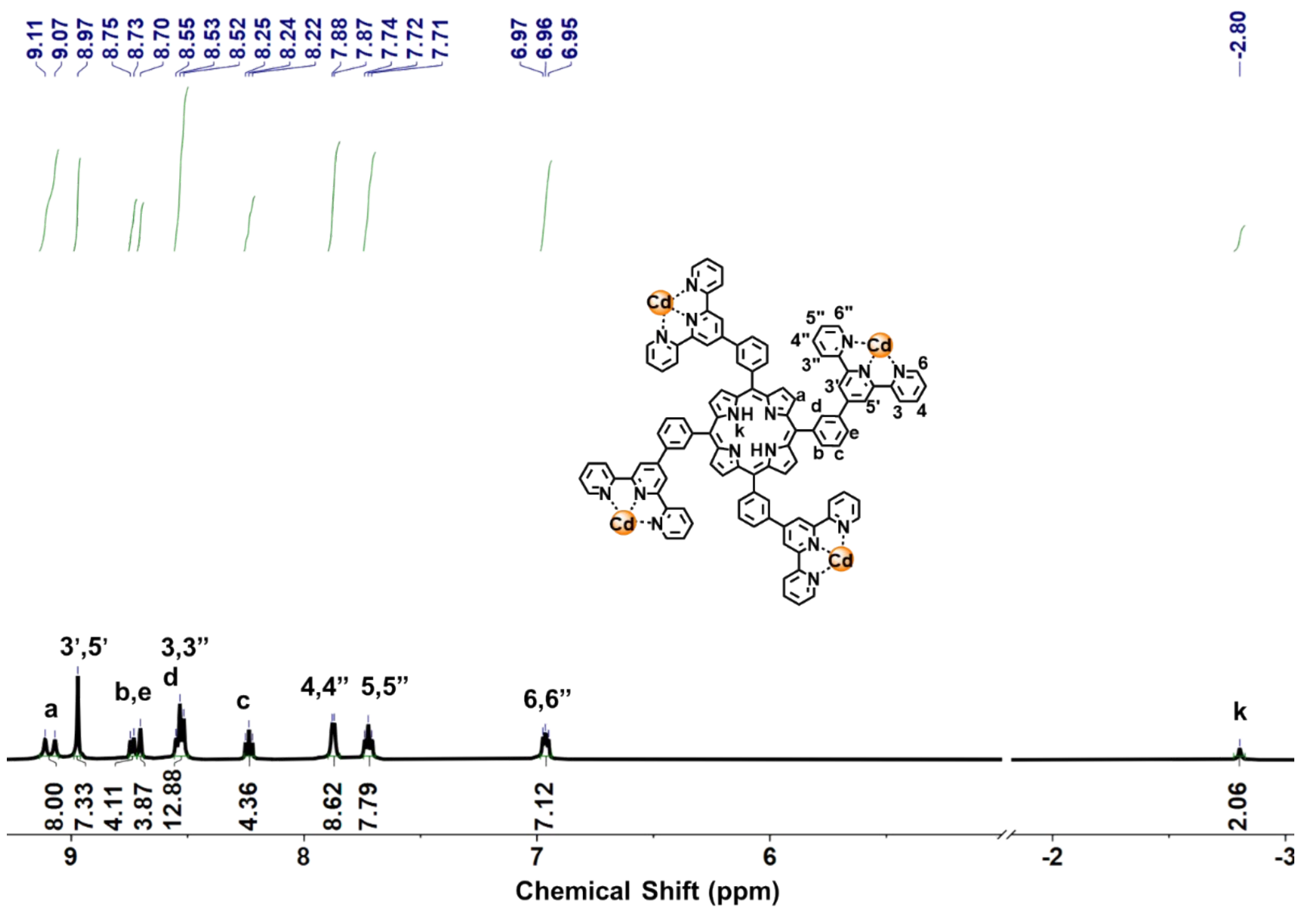

Figure S7. ^{1}H NMR (500 MHz, CD_3CN, 300 K) spectrum of metallo-organic cage **Por-Cage.**

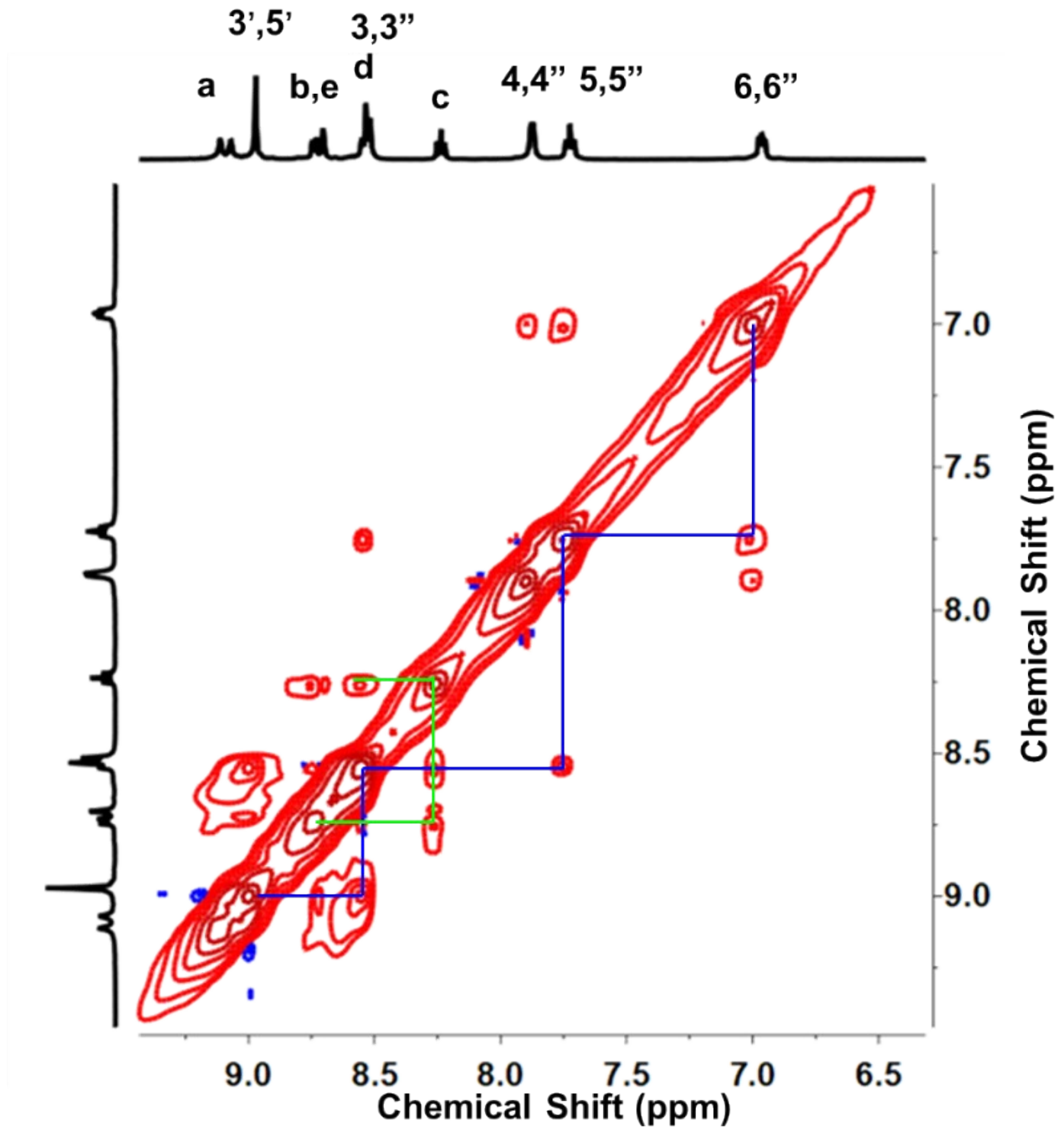

Figure S8. 2D COSY NMR (400 MHz, CD_3CN, 300 K) spectrum of metallo-organic cage **Por-Cage.**

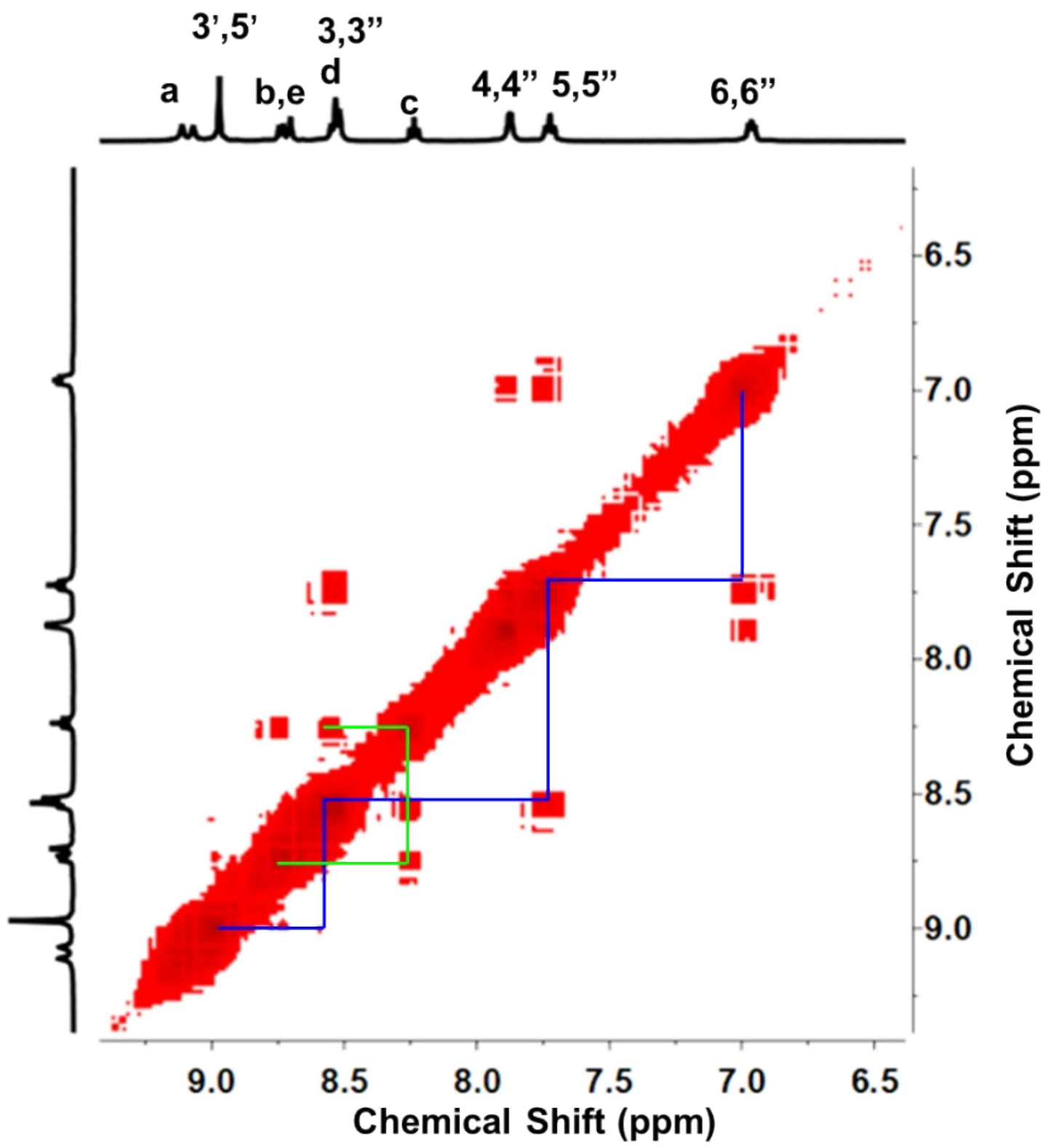

Figure S9. 2D NOESY NMR (400 MHz, CD_3CN, 300 K) spectrum of metallo-organic cage **Por-Cage.**

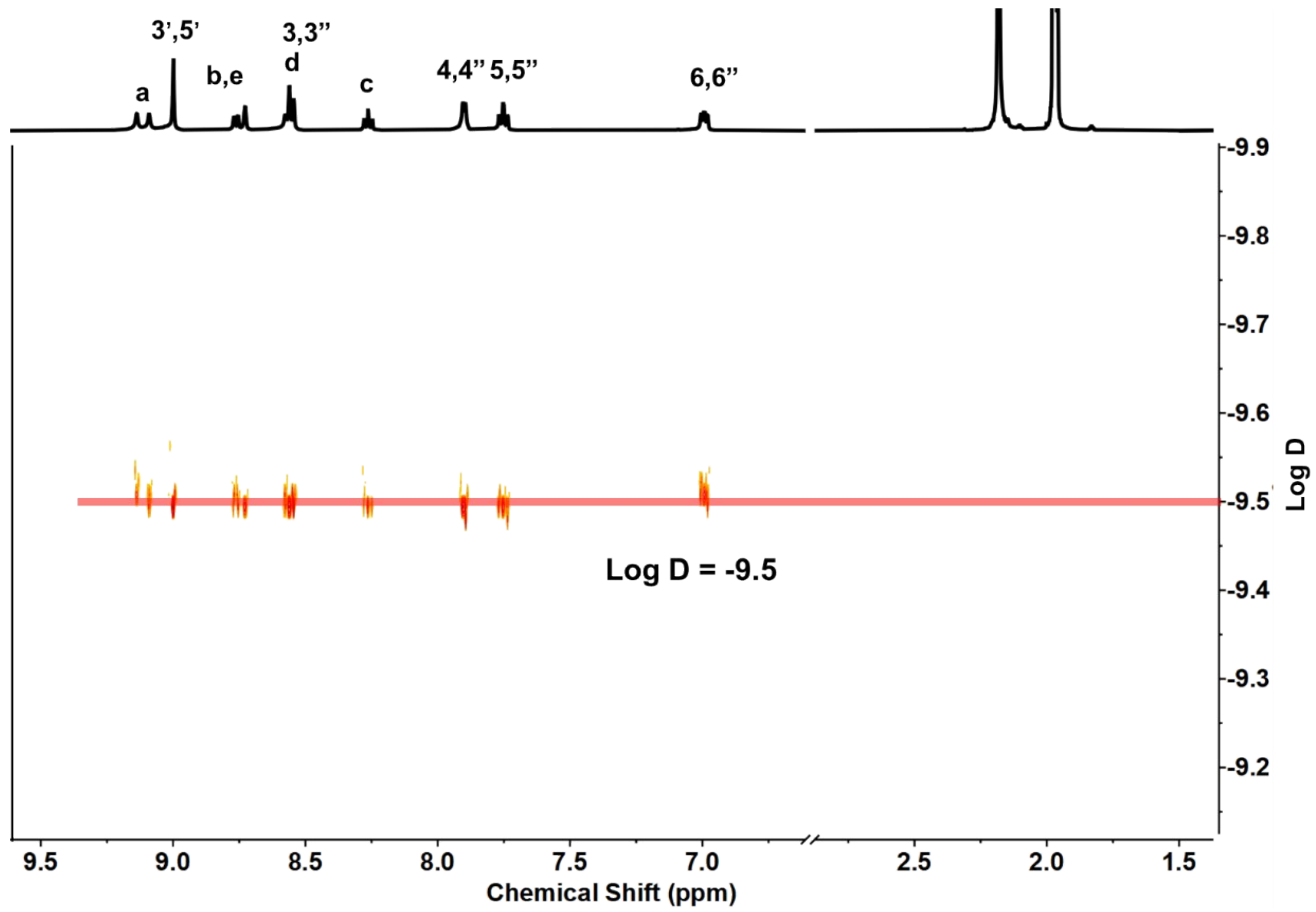

Figure S10. 2D DOSY NMR (500 MHz, CD_3CN, 300 K) spectrum of metallo-organic cage **Por-Cage.**

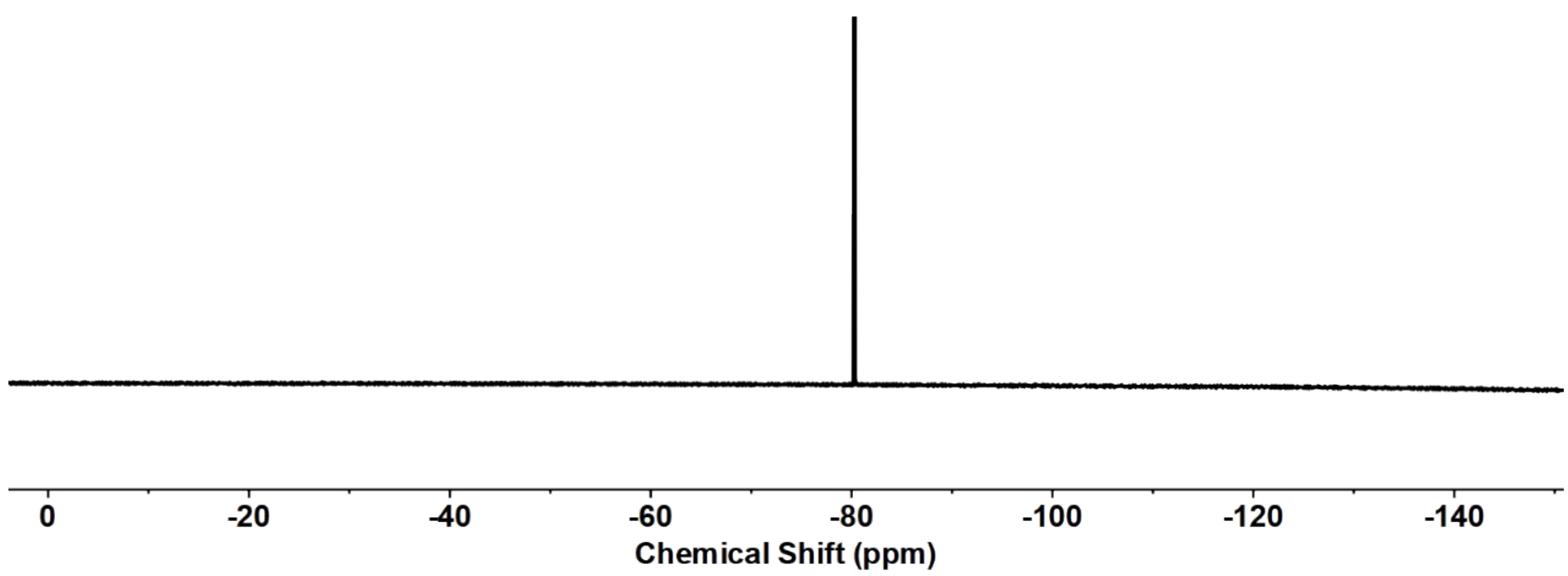

Figure S11. ^{19}F NMR (500 MHz, CD_3CN, 300 K) spectrum of metallo-organic cage **Por-Cage.**

6. Investigation of optical and electrochemical properties of Por-Cage

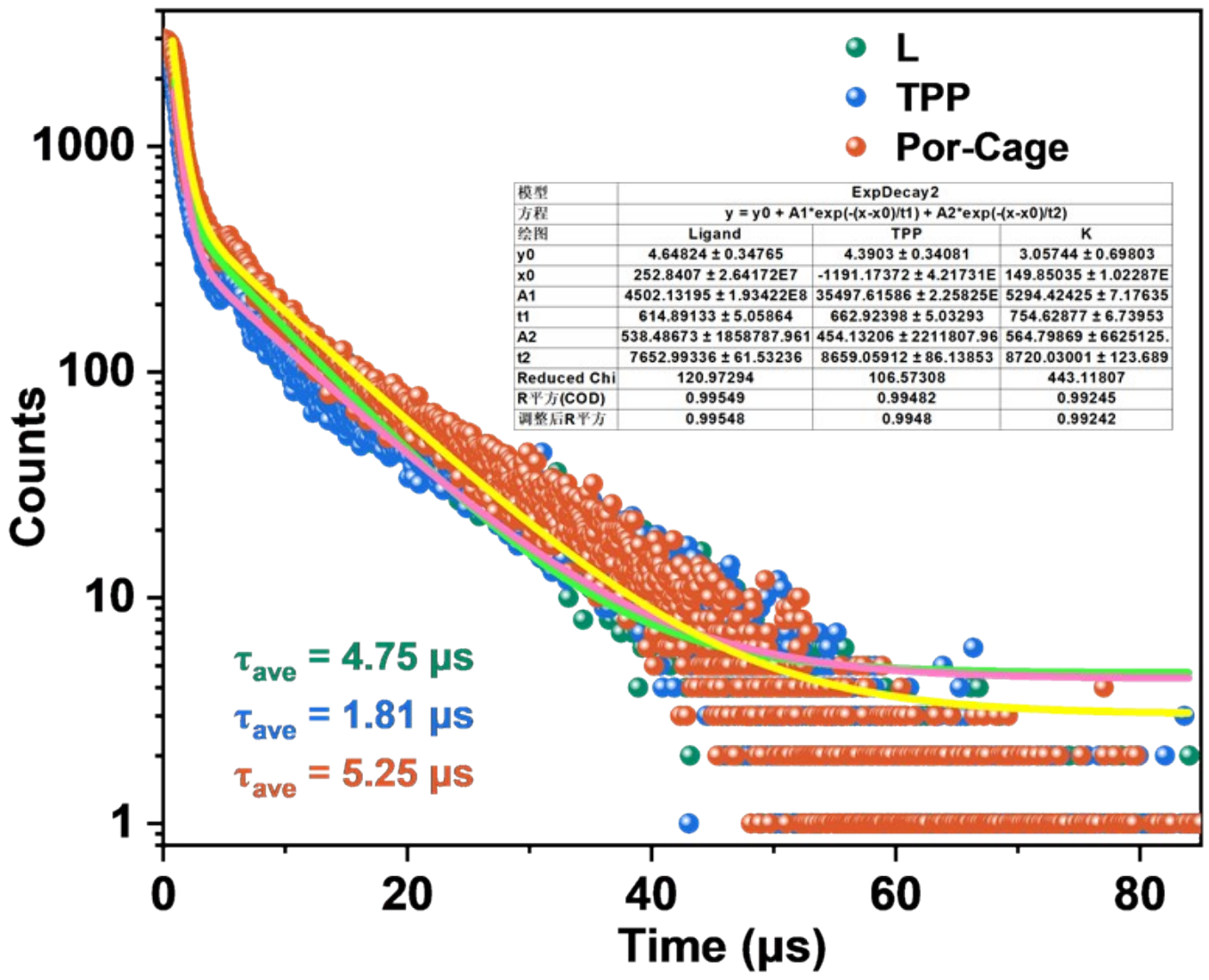

Figure S12. Fluorescence lifetime fitting curves for the metallo-organic cage **Por-Cage** in acetonitrile, ligand **L** and **TPP** in chloroform.

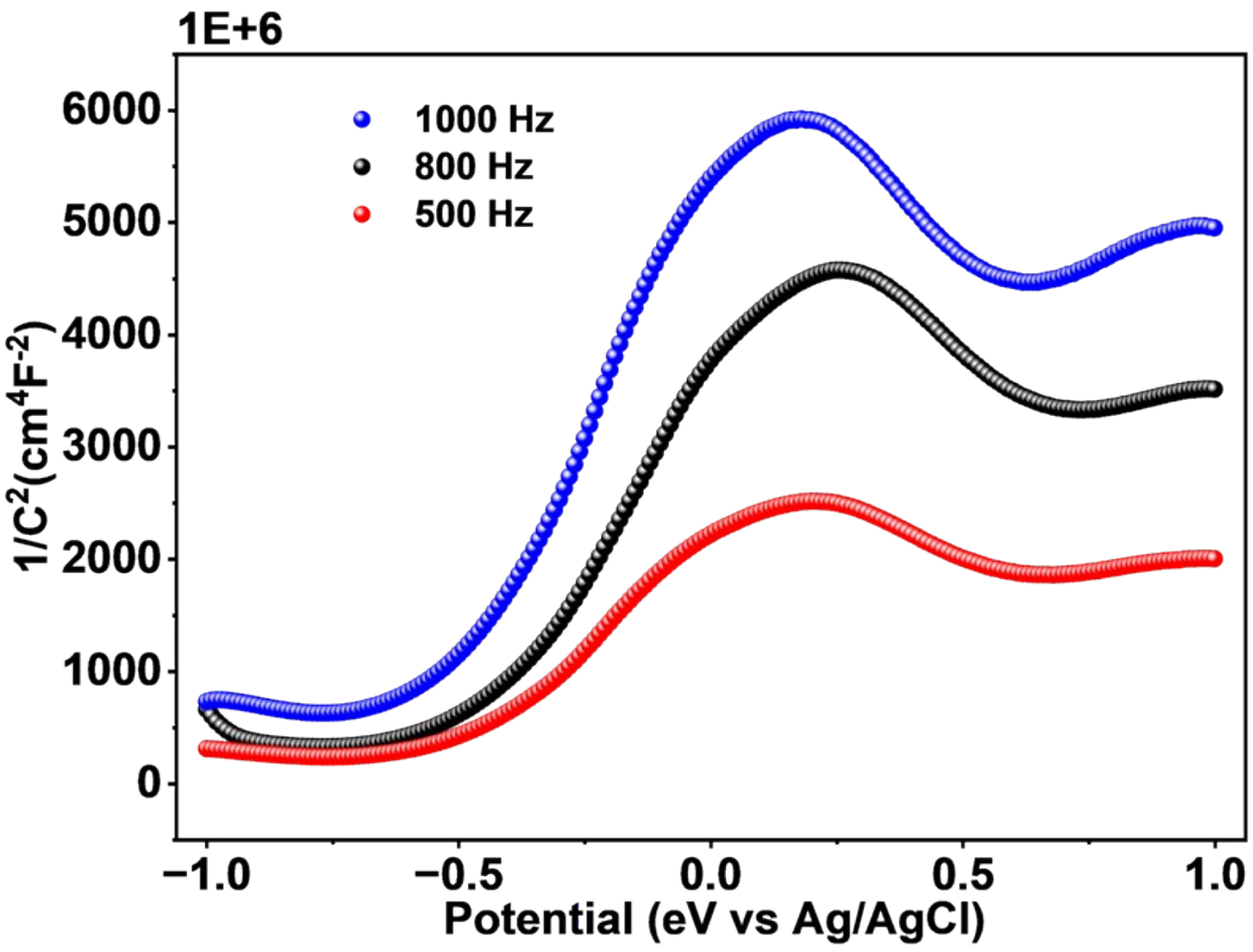

Figure S13. Mott-Schottky curves of the metallo-organic cage **Por-Cage** at different frequencies.

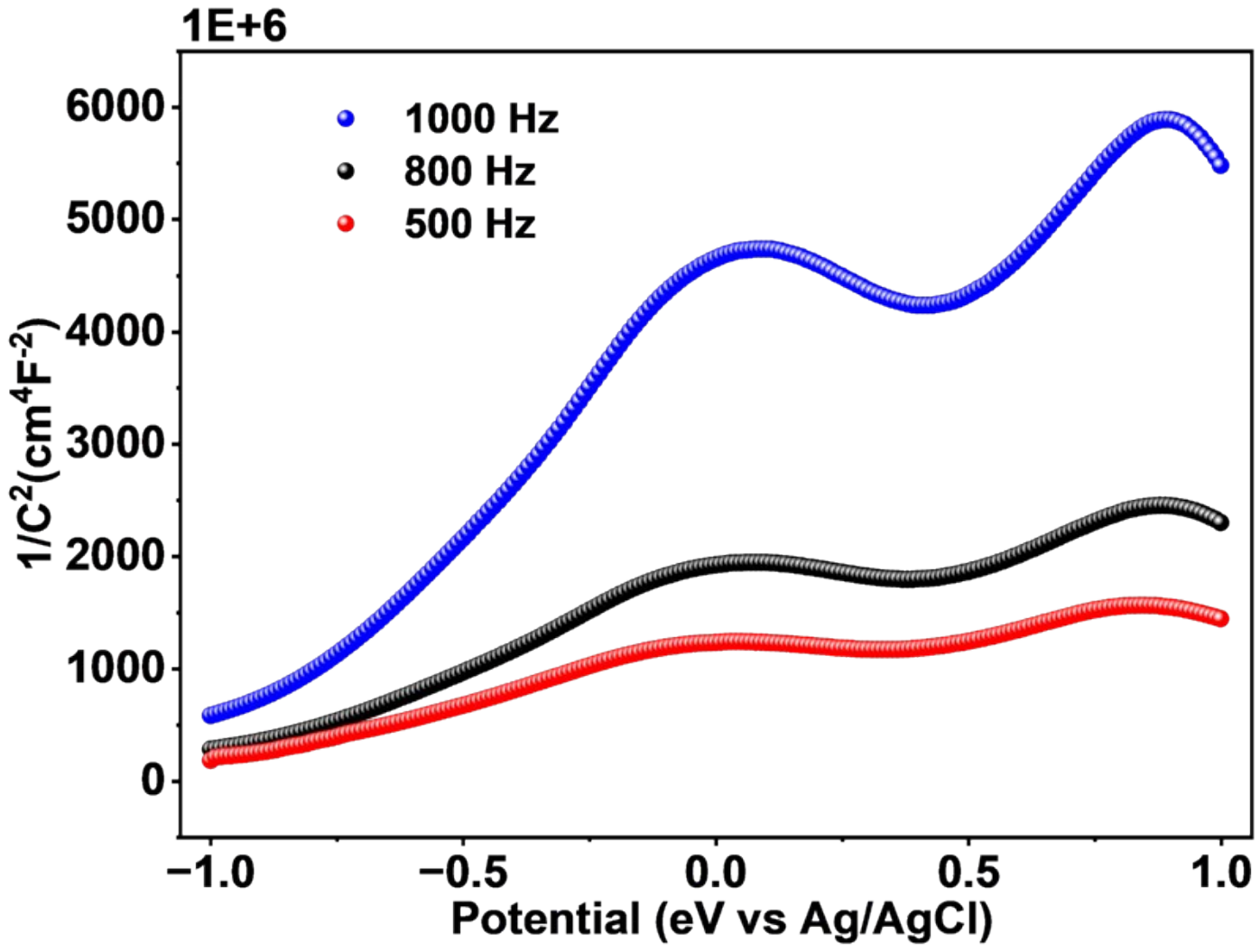

Figure S14. Mott-Schottky curves of the ligand **L** at different frequencies.

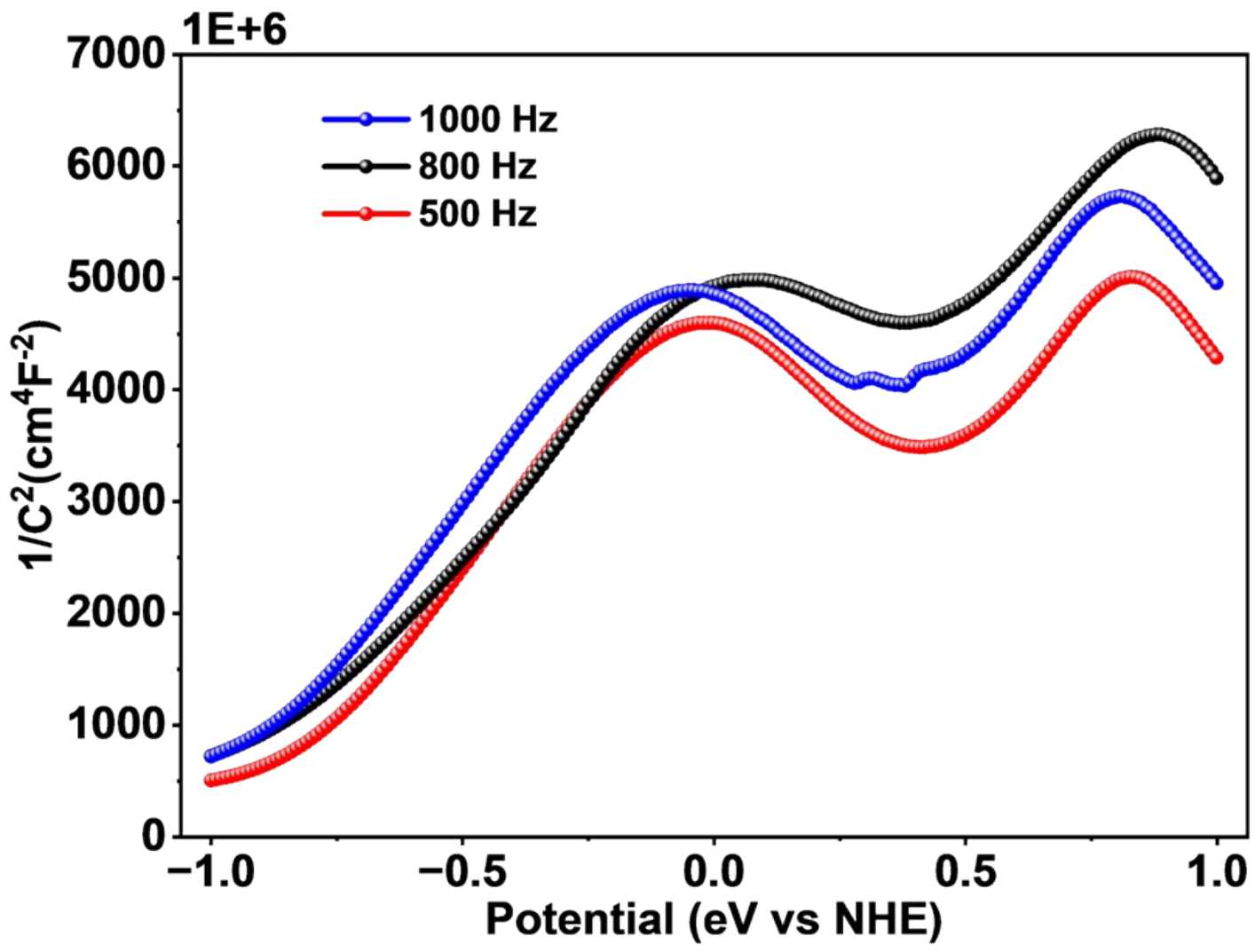

Figure S15. Mott-Schottky curves of the **TPP** at different frequencies.

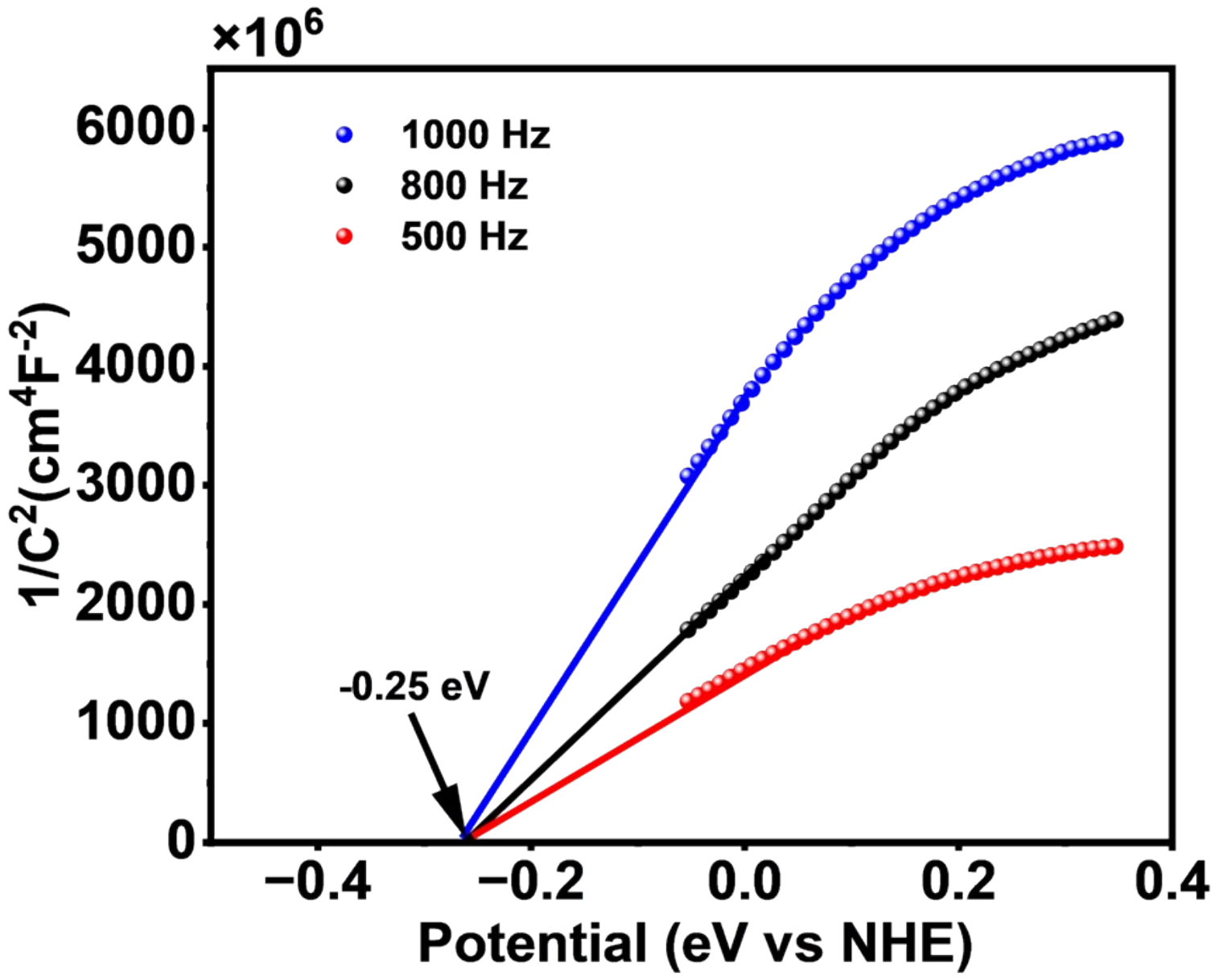

Figure S16. Mott-Schottky curves of the **Por-Cage** at different frequencies.

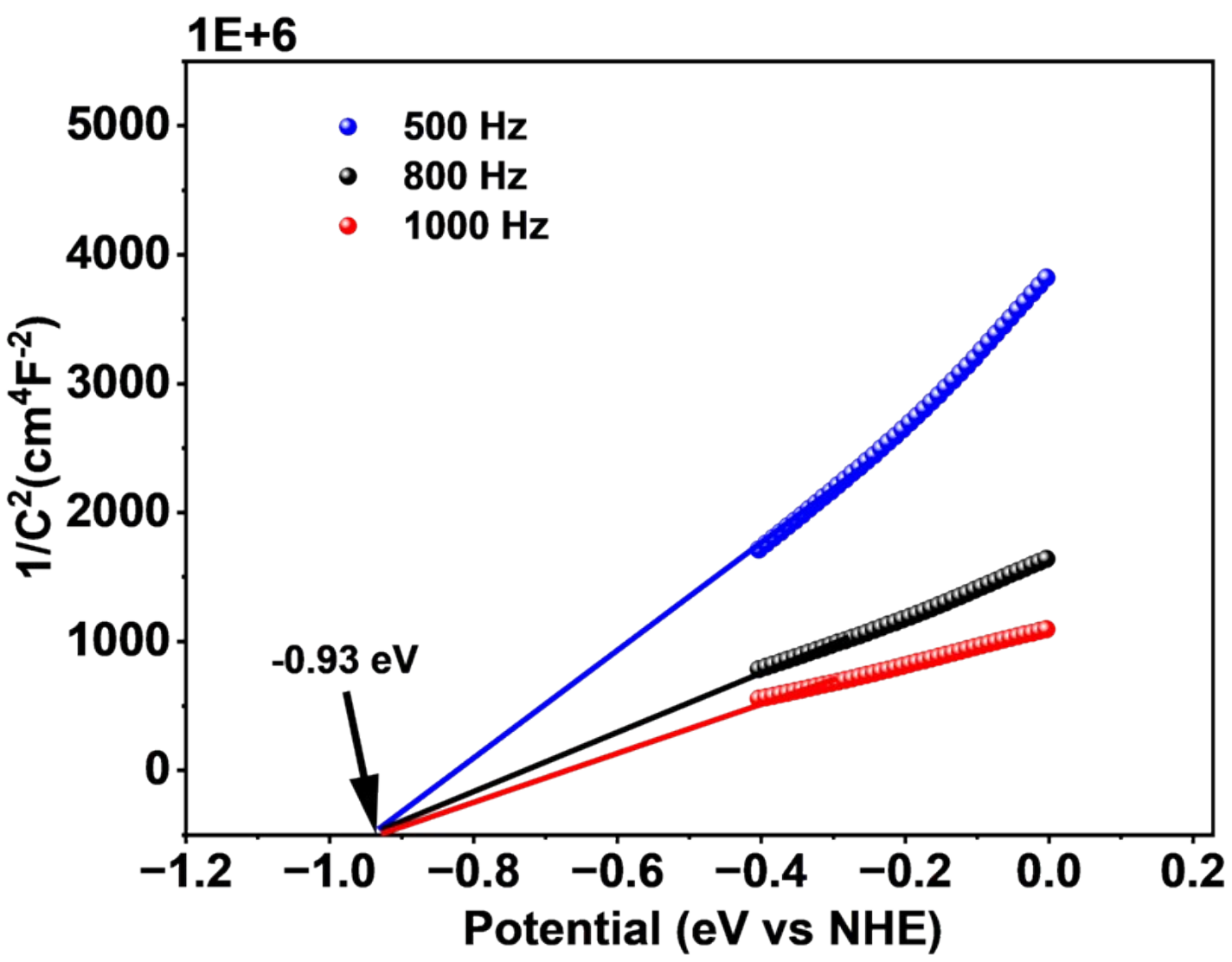

Figure S17. Mott-Schottky curves of the ligand **L** at different frequencies.

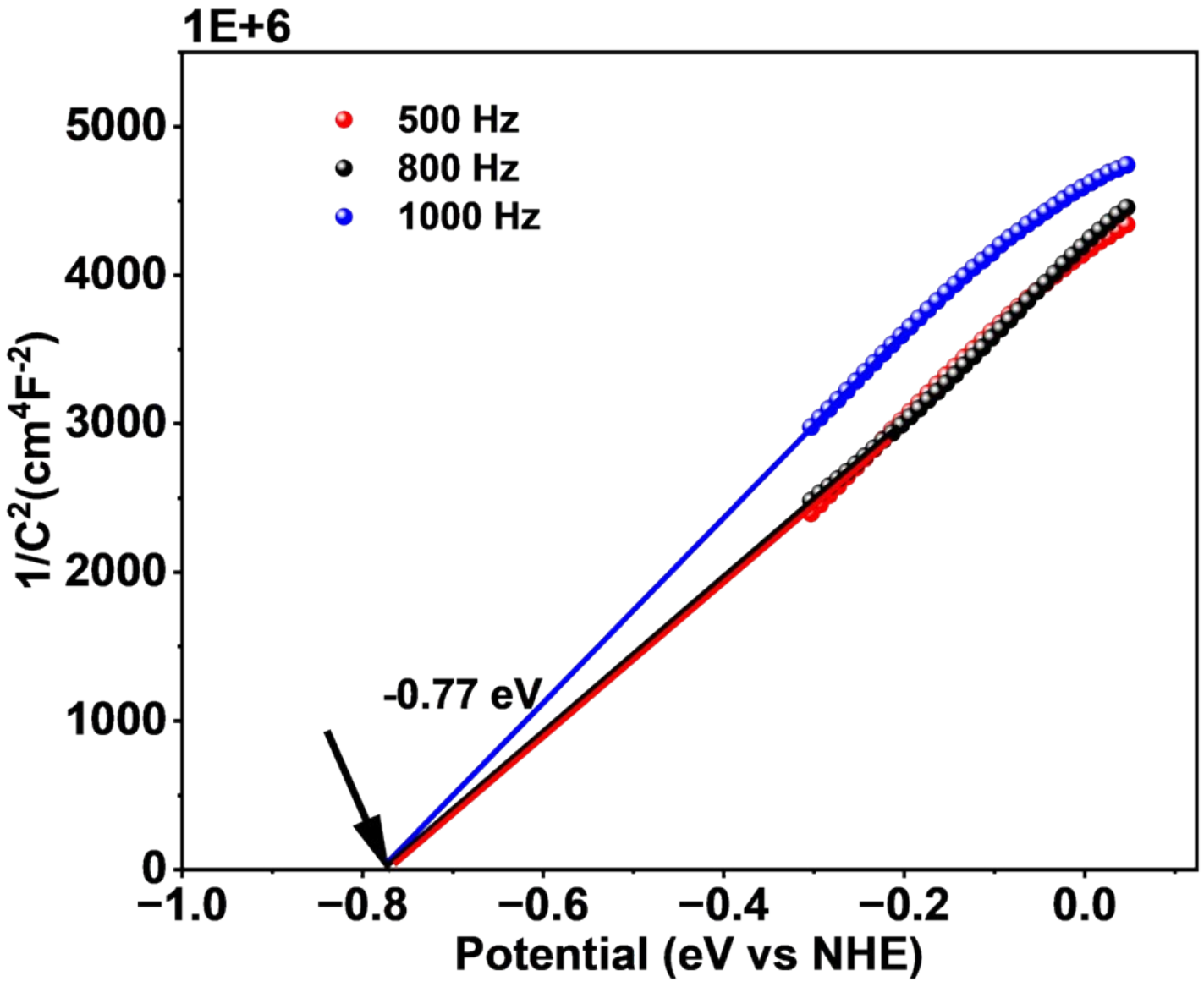

Figure S18. Mott-Schottky curves of the **TPP** at different frequencies.

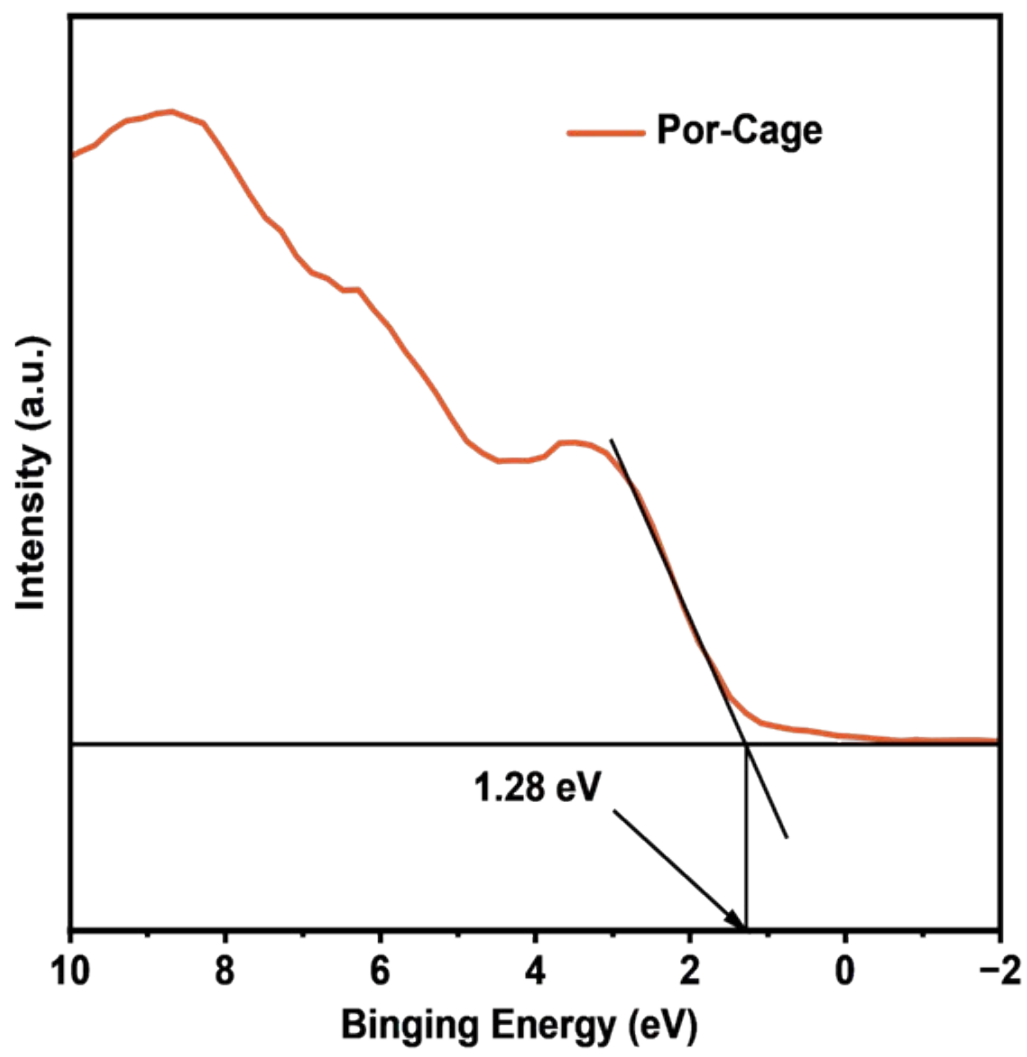

Figure S19. Valence band spectrum of **Por-Cage**. The corresponding EVB XPS of **Por-Cage** is measured to be 1.28 eV. Then, the EVB vs. standard hydrogen electrode (EVB, NHE) can be calculated according to the following formula: EVB NHE = φ + EVB XPS – 4.44. where φ is the work function of the instrument (4.2 eV). Thus, the EVB NHE of **Por-Cage** is calculated to be 1.04 eV.

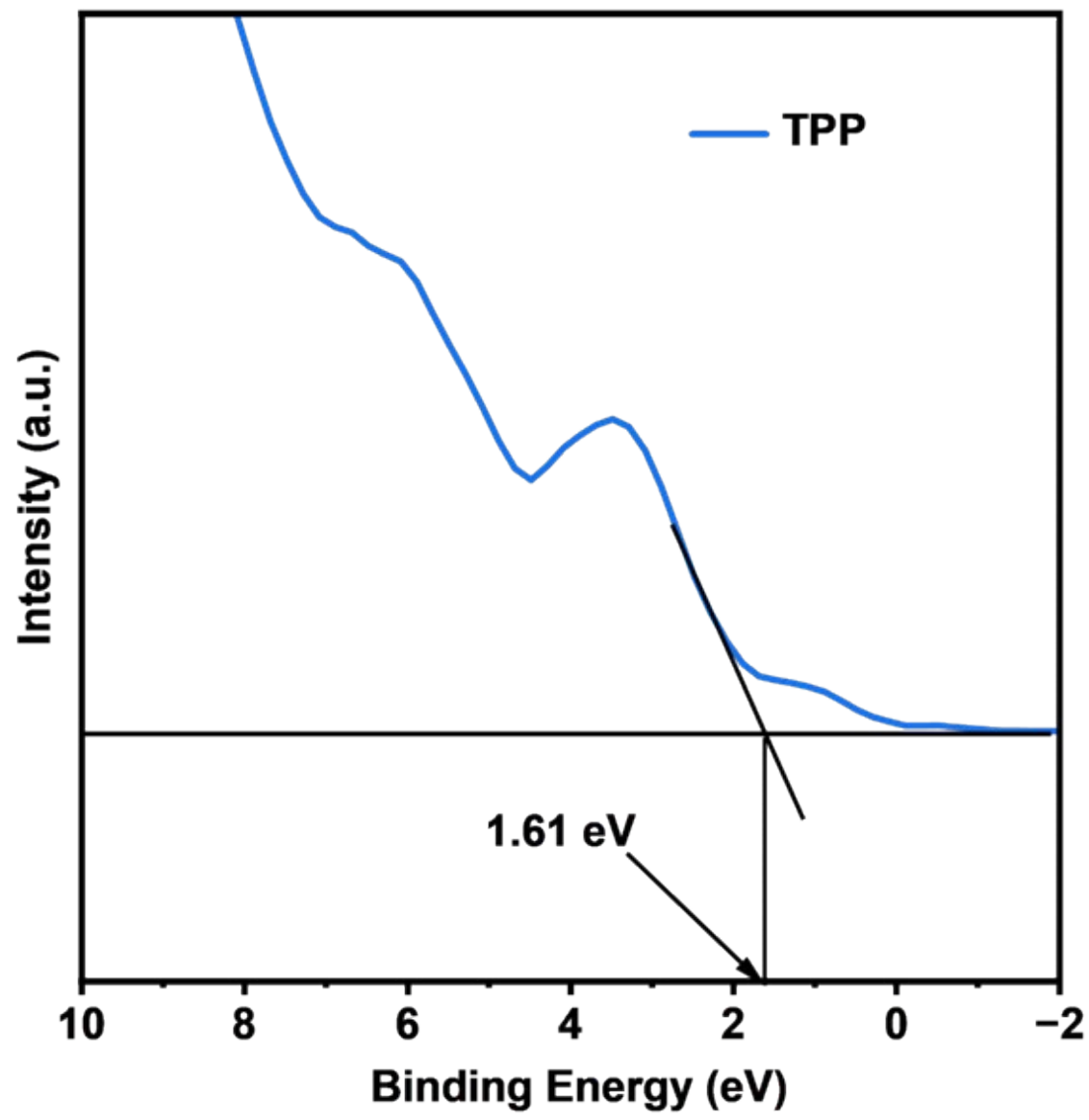

Figure S20. Valence band spectrum of **TPP**. The corresponding EVB XPS of **TPP** is measured to be 1.61 eV. Then, the EVB vs. standard hydrogen electrode (EVB, NHE) can be calculated according to the following formula: EVB NHE = φ + EVB XPS – 4.44. where φ is the work function of the instrument (4.2 eV). Thus, the EVB NHE of **TPP** is calculated to be 1.37 eV.

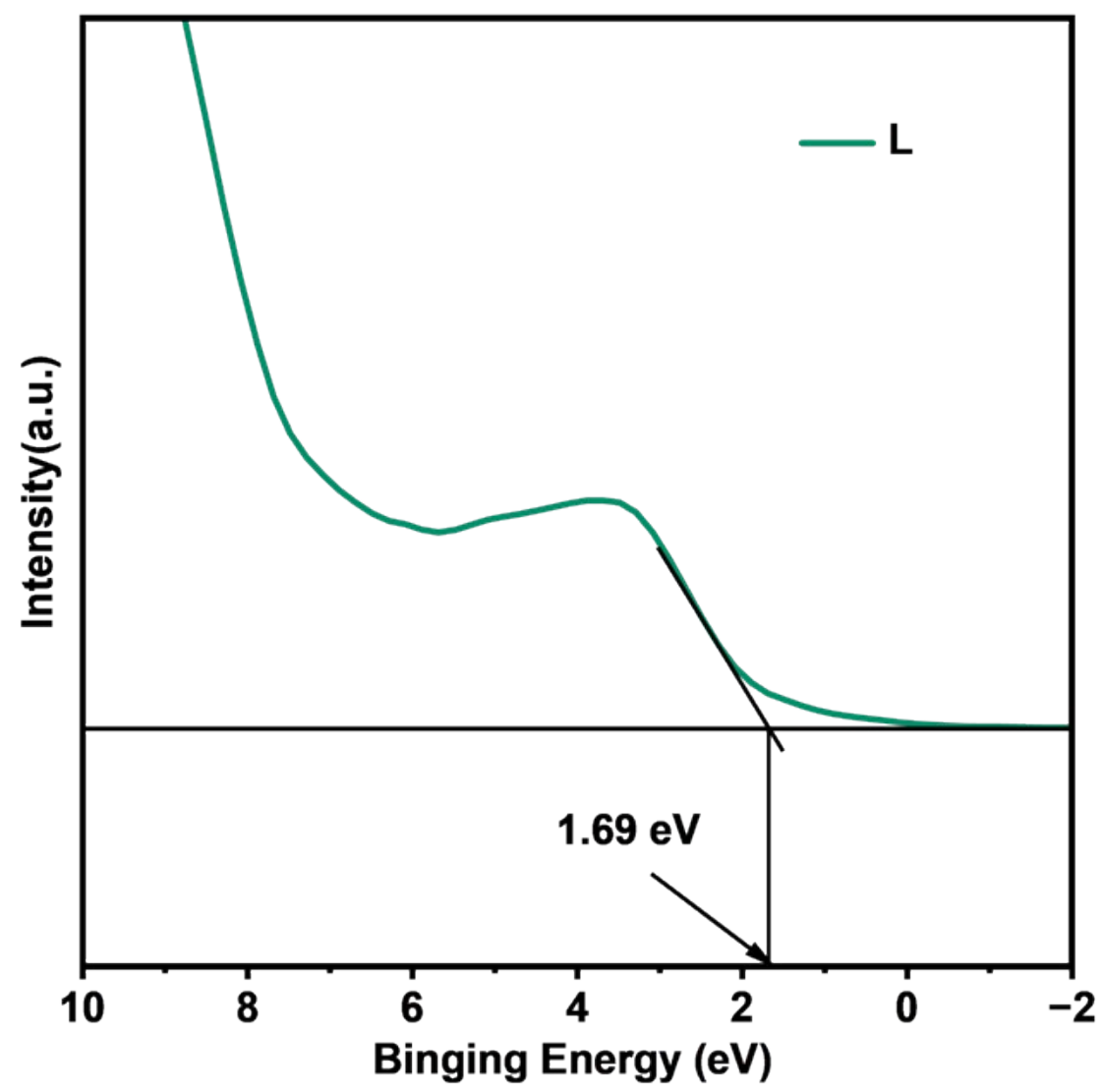

Figure S21. Valence band spectrum of **L**. The corresponding EVB XPS of **L** is measured to be 1.69 eV. Then, the EVB vs. standard hydrogen electrode (EVB, NHE) can be calculated according to the following formula: EVB NHE = φ + EVB XPS – 4.44. where φ is the work function of the instrument (4.2 eV). Thus, the EVB NHE of **TPP** is calculated to be 1.45eV.

The bottle was irradiated by a multi-channel photoreaction system 4-HZJ (Beijing Perfect Light Technology Co., LTD., Beijing, China, PCX50C). The apparent quantum yield (AQY) of the photocatalyst was measured with 385 nm, 420 nm, 450 nm, 485 nm, and 520 nm bandpass filters. The active area of the reactor is about 5.72 cm^2. Use an optical radiometer to take the average value of monochromatic light intensity at three representative points. Therefore, the light intensity at 385 nm, 420 nm, 450 nm, 485 nm, and 520 nm is calculated as 0.313, 0.262, 0.269, 0.562, and 0.569 W•cm^{-2}, respectively. AQY is calculated as follows:

$$AQY = \frac{n_{CEESO} \times N_A}{I \times A \times t \times \frac{\lambda}{hc}} \times 100\%$$

n = number of produced CEESO molecules (mol)

$\mathbf{N_A}$ (Avogadro constant) = 6.02 × 10^{23} mol^{-1}

I = the intensity difference of irradiation light (W•cm^2) = 0.269 W•cm^2(450 nm)

A = Irradiation area (cm^2) = 5.72 cm^2

t = the photoreaction time (s) = 1200 s

λ = the wavelength of the monochromatic light (m) = 450 × 10^{-9} m

h (Planck constant) = 6.626 × 10^{-34} J·s

c (Speed of light) = 3 × 108 m•s^{-1}

Table S1. Parameter values in AQY calculations.

λ	I	Conversion	n(CEESO)	AQY(%)
385	0.313	30 %	75.0 μmol	1.08
420	0.262	28 %	70 μmol	1.11
450	0.269	33 %	82.5 μmol	1.19
485	0.562	57 %	142.5 μmol	0.91
520	0.569	55 %	137.5 μmol	0.81

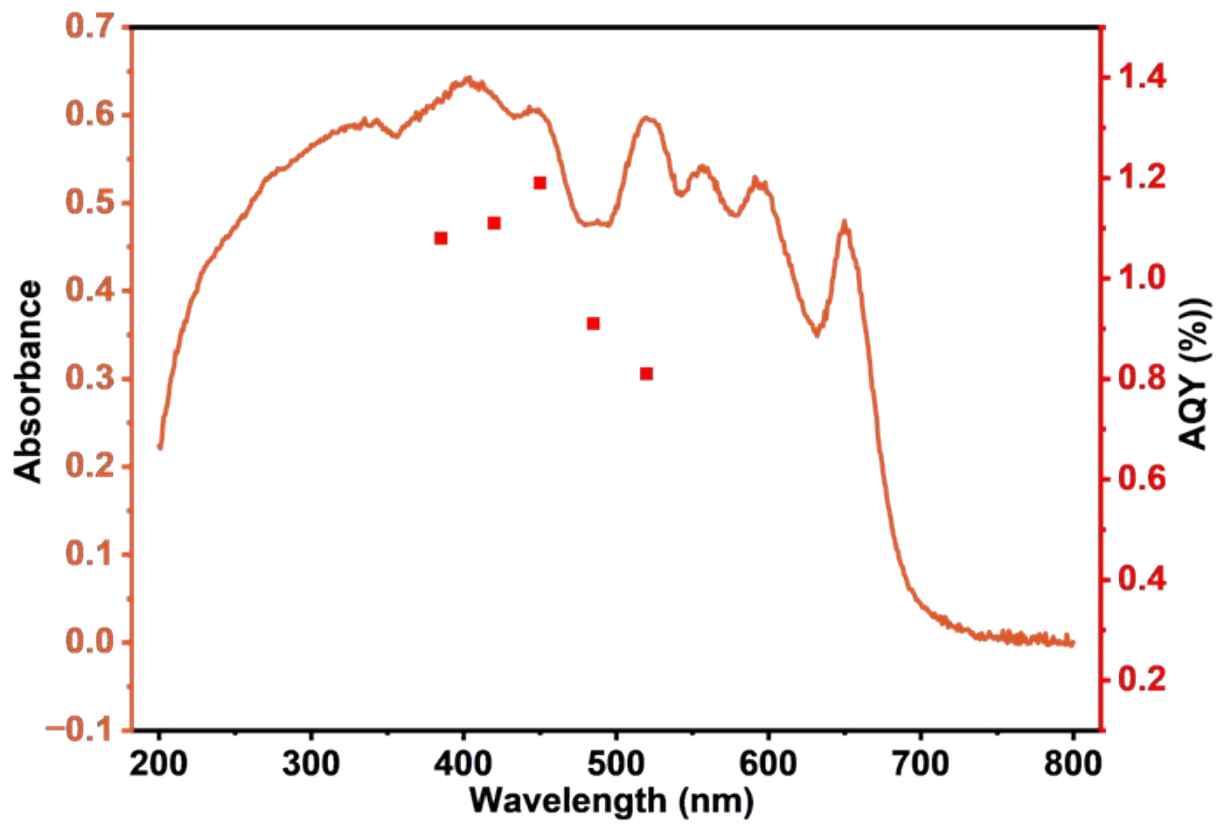

Figure S22. Wavelength-dependent AQY values (measured in 20 min) and solid state UV-visible spectrum of **Por-Cage**.

The $^1O_2$ production specific calculation process is as follows:

Experiments were performed using a classic $^1O_2$ probe molecule DMA (9,10-dimethylanthracene). The ratio of catalysis: DMA was all kept to be 1 : 100 by changing the amount of three catalysts. $[Ru(bpy_3)]^{2+}$ was used as a reference sensitizer ($\Phi_{\triangle}$=73 %)[7]. The quantum yields were calculated according to the following equations,

$$\Phi_{\triangle}^{S} = \Phi_{\triangle}^{R} \frac{S^S F^R}{F^R S^R}$$

$$F = 1 - 10^{-OD420nm}$$

where S denotes the calibrated slope of the linear fit of the cumulative changes of the optical intensity at 420 nm vs. the cumulative irradiation time. F stands for the absorption correction factor, OD corresponds to the absorbance of the photosensitizer. The superscript "S" denotes the samples, and "R" denotes the reference, i.e. $[Ru(bpy_3)]^{2+}$. The singlet oxygen quantum yield ($\Phi_{\triangle}$) has been further quantified by monitoring the degradation of DMA, giving relative value of 79.12%, 49.05%, and 16.61% for **Por-Cage**, **TPP**, and **L** respectively.

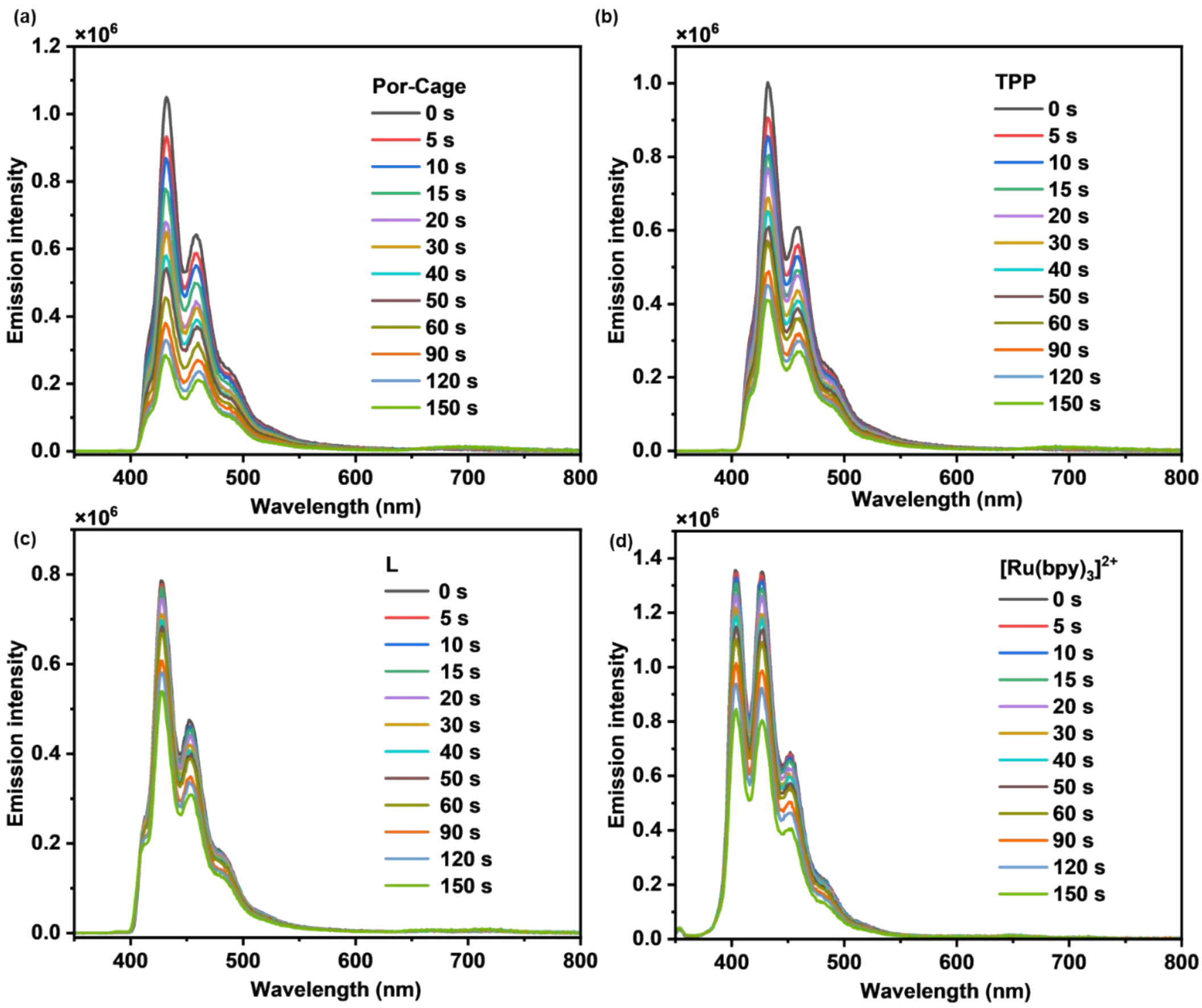

Figure S23. Emission spectra of DMA in the presence of (a) **Por-Cage**, (b) **TPP**, (c) **L** and (d) **$[Ru(bpy_3)]^{2+}$** in CH_3OH at equal Mn unit mols (λex=320 nm) irradiating with white light.

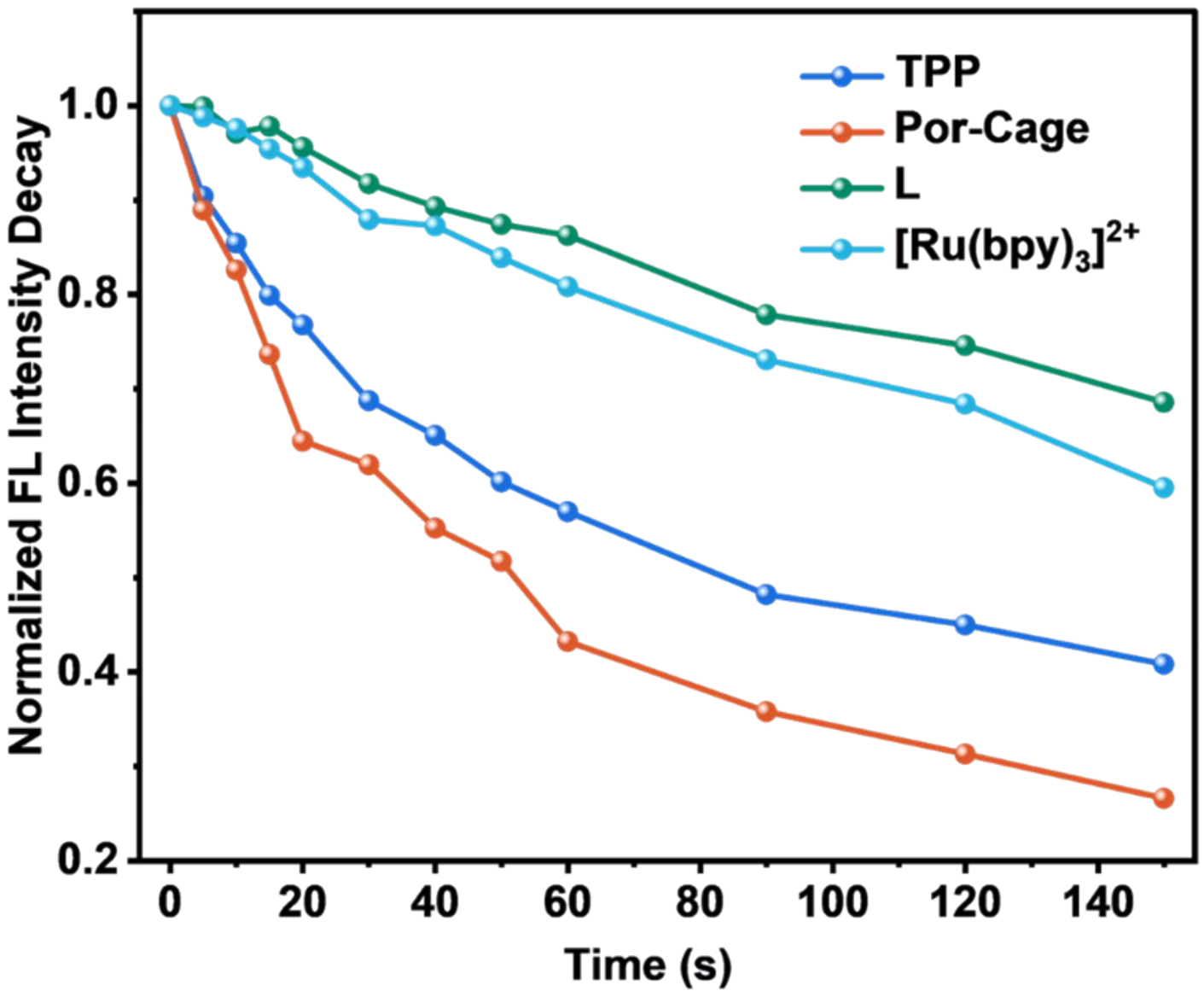

Figure S24. The plots for the FL intensity decays of DMA using **Por-Cage**, **TPP**, **L** and **$[Ru(bpy_3)]^{2+}$** as monitored by the emission at 432 nm.

Table S2. Specific values for single line state oxygen production rate calculations

	S	OD	F	Φ (%)
**[Ru(bpy$_3$)]$^{2+}$**	-0.0044	0.1245	0.2492	73
**Por-Cage**	-0.0104	0.3405	0.5434	79.12
**TPP**	-0.0086	0.5650	0.7249	49.05
**L**	-0.0030	0.5962	0.7466	16.61

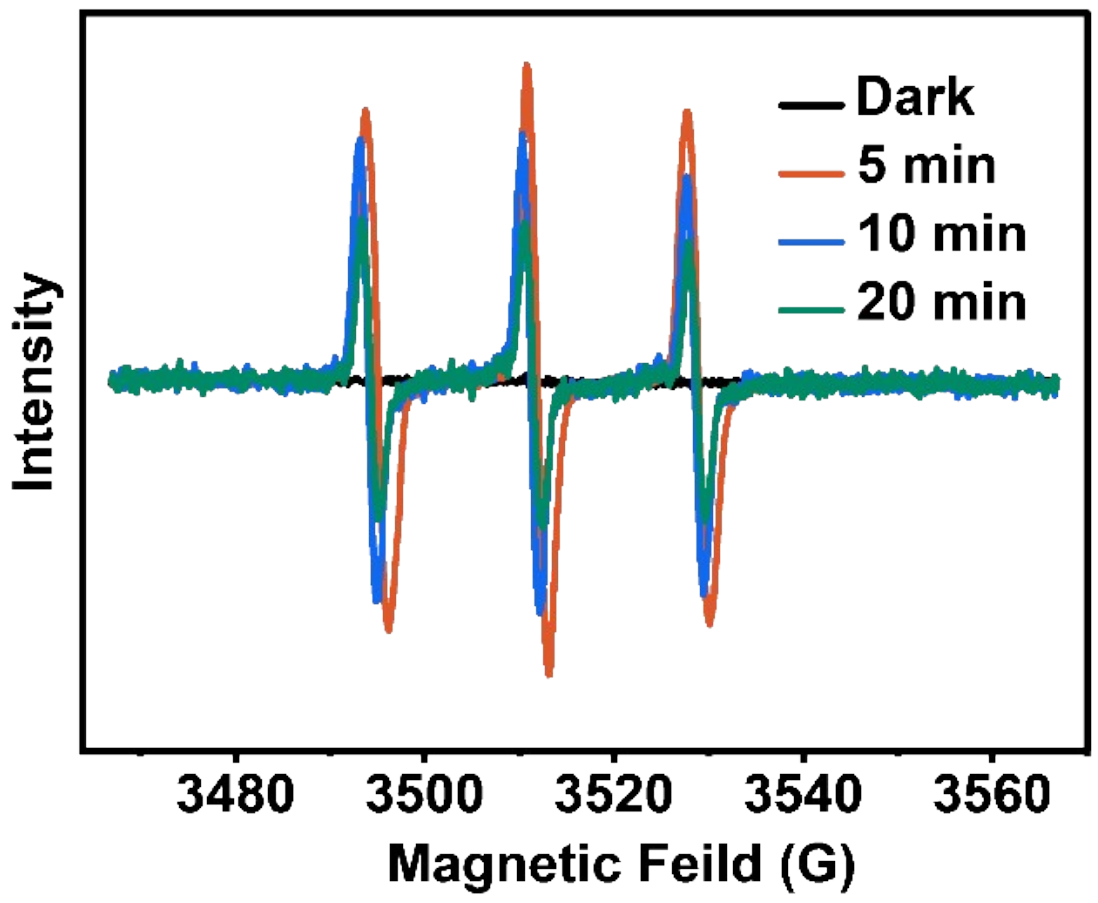

Figure S25. EPR spectra of **Por-Cage** mixed with 4-oxo-TMP under visible light irradiation at dark, 5 min, 10 min and 20 min.

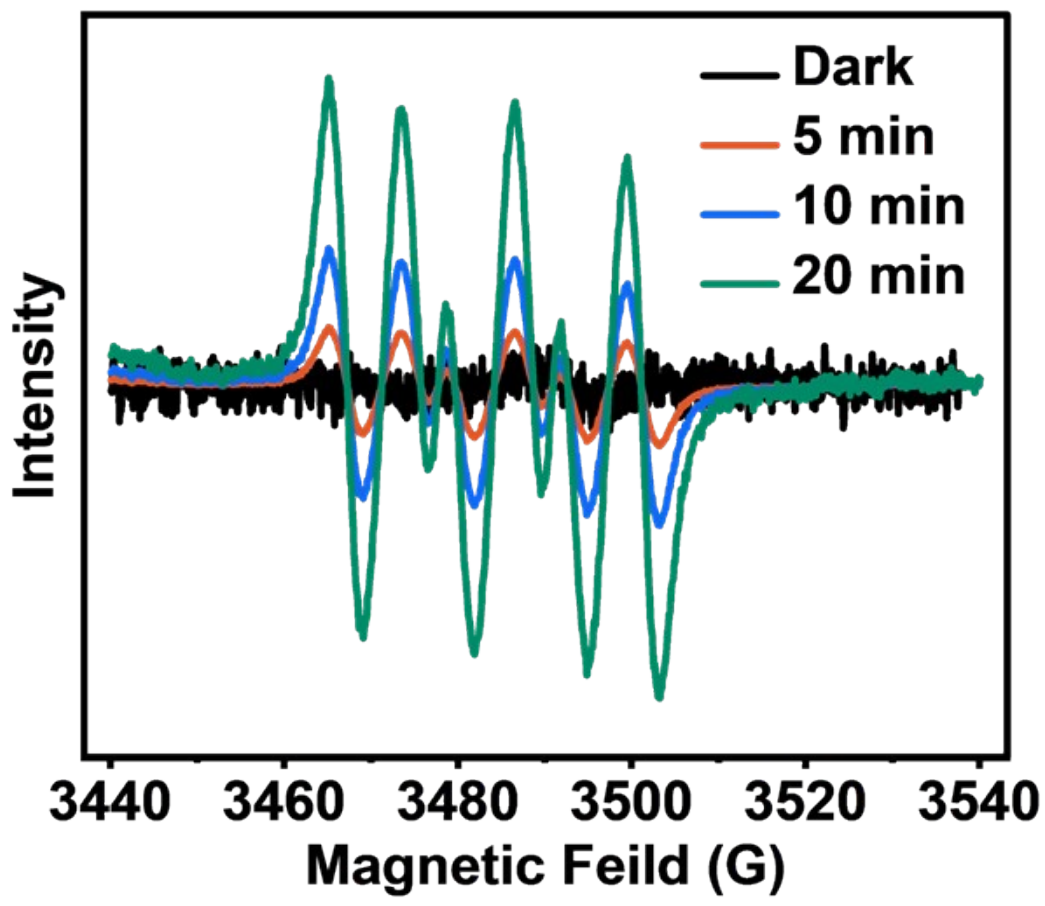

Figure S26. EPR spectra of **Por-Cage** mixed with DMPO under visible light irradiation at dark, 5 min, 10 min and 20 min.

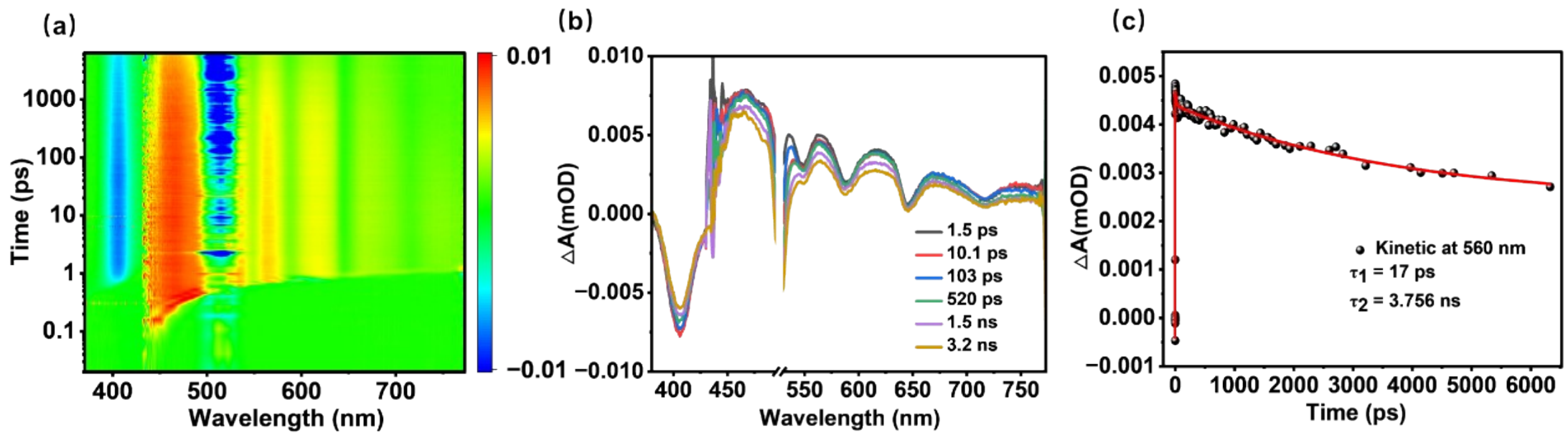

Figure S27. (a) 2D contour plot of ns-TA for **Por-Cage**; (b) fs-Transient absorption (TA) spectra of **Por-Cage** in CH_3CN at various time delays after excitation at 515 nm; (c) Decay kinetic curves of **Por-Cage** at 450 nm.

7. Investigation of the Photocatalytic Properties of the Metallo-Organic Cage Por-Cage

Since all three catalysts are insoluble in methanol, CD_3OD was selected as the reaction solvent for heterogeneous catalysis. **Por-Cage** (7.14 mg, 0.83 μmol) was dispersed in 1 mL CD_3OD. 30 μL (250 μmol) of CEES dissolved in CD_3OD were added to the vial, which was then placed under white light LED illumination. The reaction was monitored via NMR spectroscopy.

Control experiments: Ligand **L** (3.8 mg, 0.0025 mmol) or **TPP** (1.60 mg, 0.0025 mmol) was dispersed in 1 mL CD_3OD and transferred to an open glass reactor. Then, 30 μL (250 μmol) of CEES dissolved in CD_3OD was added to the glass reactor. The mixture was placed under white light LED illumination and monitored by NMR spectroscopy.

Cyclic testing: After centrifuging the reaction system, thoroughly washing it with water and methanol, and drying it, the next round of testing can proceed.

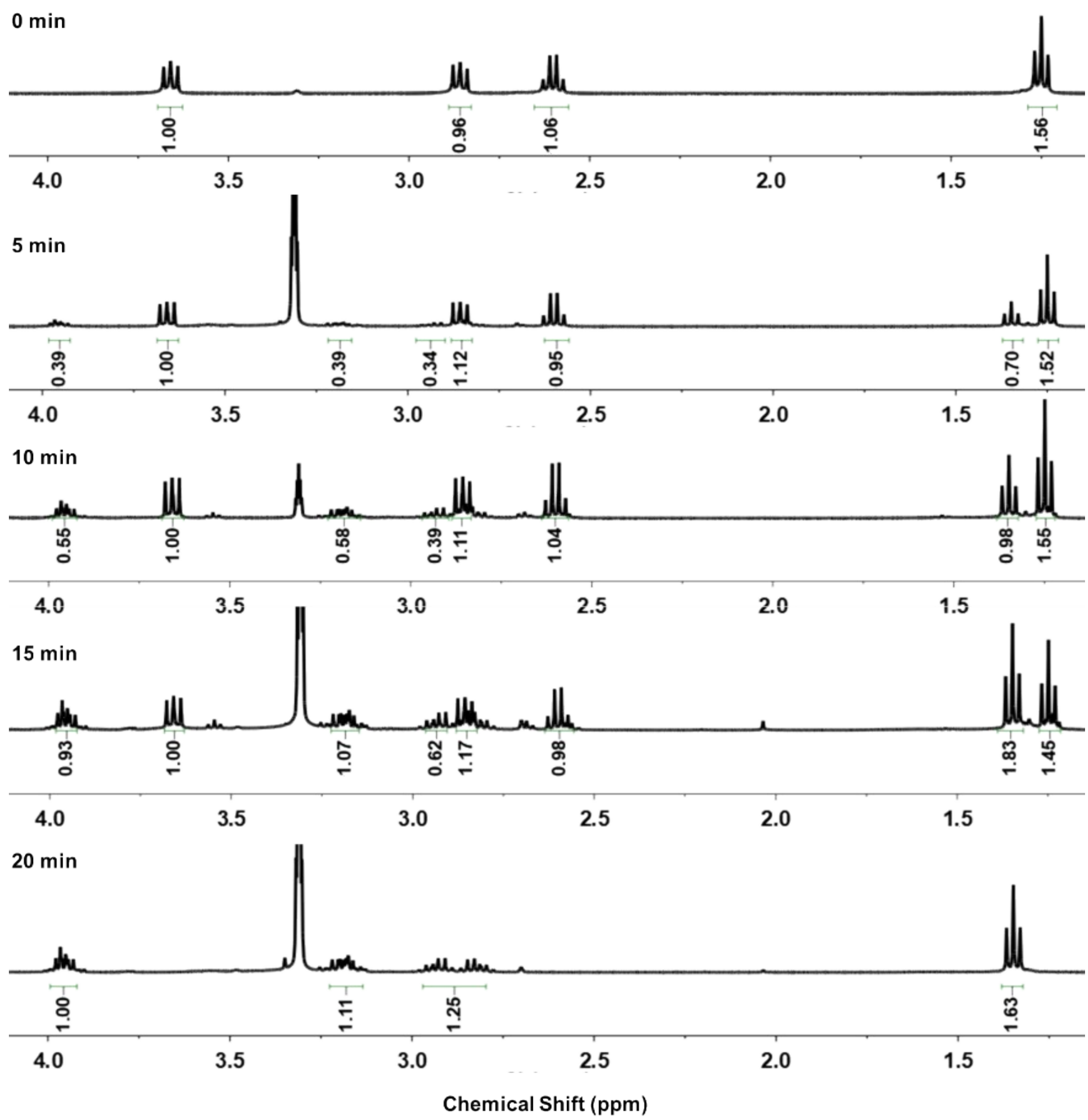

Figure S28. ^{1}H NMR spectrum of the oxidation reaction in the metallo-organic cage **Por-Cage** heterogeneous photocatalytic CEES system.

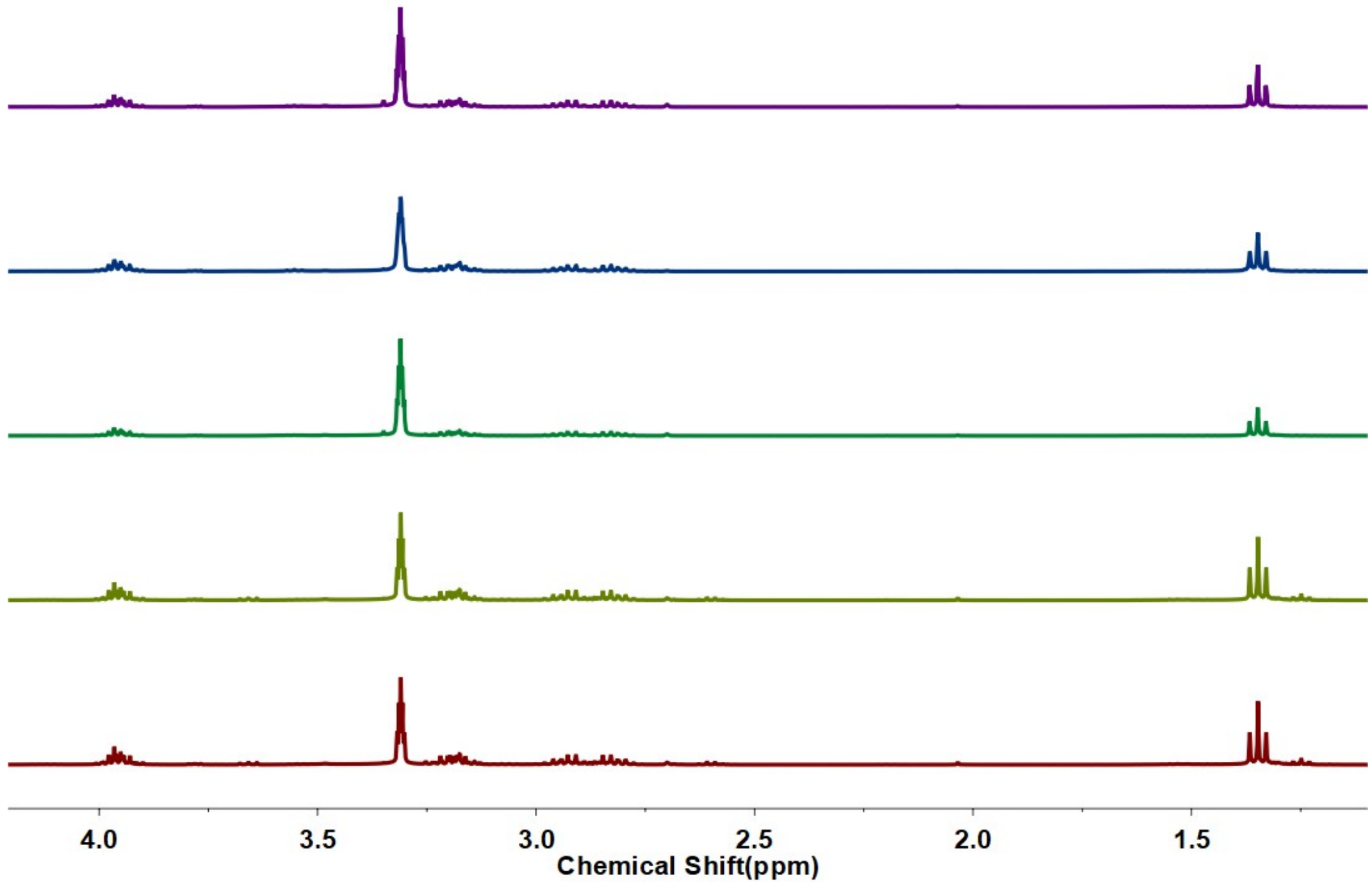

Figure S29. ^{1}H NMR spectra of the metallo-organic cage **Por-Cage** heterogeneous photocatalytic oxidation of CEES over five cycles.

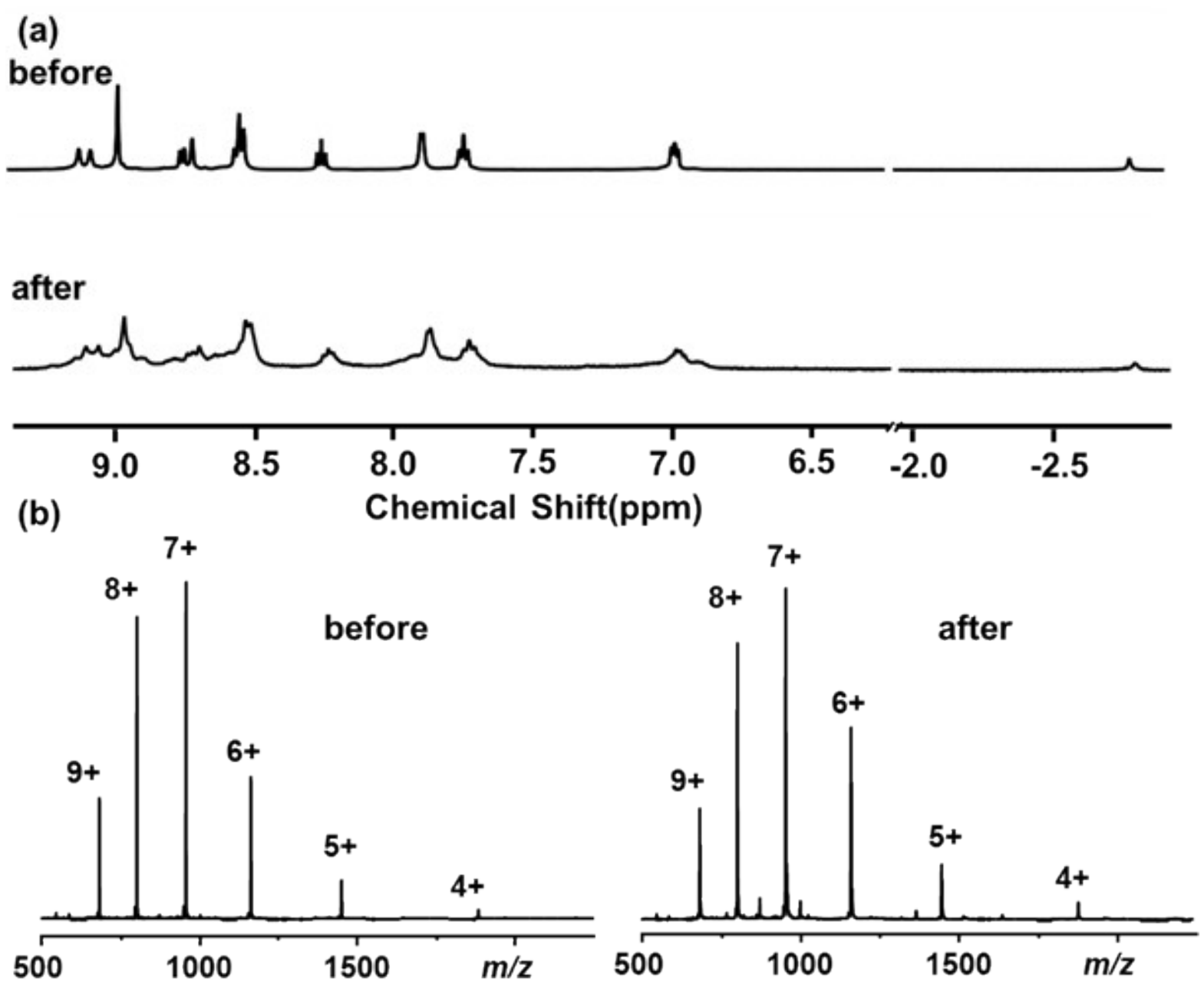

Figure S30. ^{1}H NMR spectra of metallo-cage **Por-Cage** before catalysis and after 5 cycles (500 MHz，CD_3CN, 300 K); (b) ESI-MS spectra of metallo-cage **Por-Cage** before catalysis and after 5 cycles.

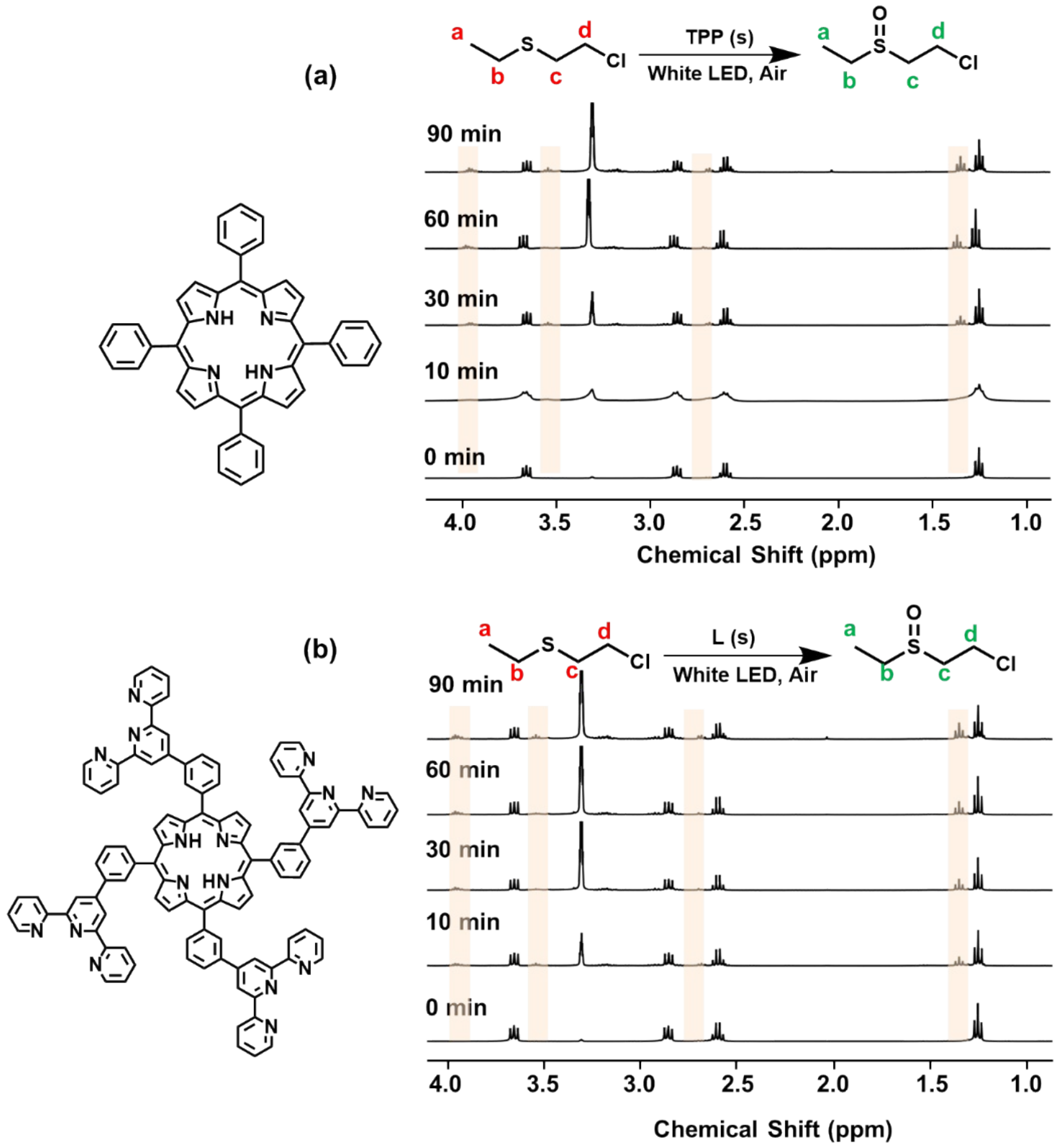

Figure S31. (a) ^{1}H NMR analysis of the photo-oxidation of CEES by **TPP** at solid states (400 MHz, CD_3OD, 300 K) (400 MHz, $CDCl_3$, 300 K); (b) ^{1}H NMR analysis of the photo-oxidation of CEES by ligand **L** at solid states.

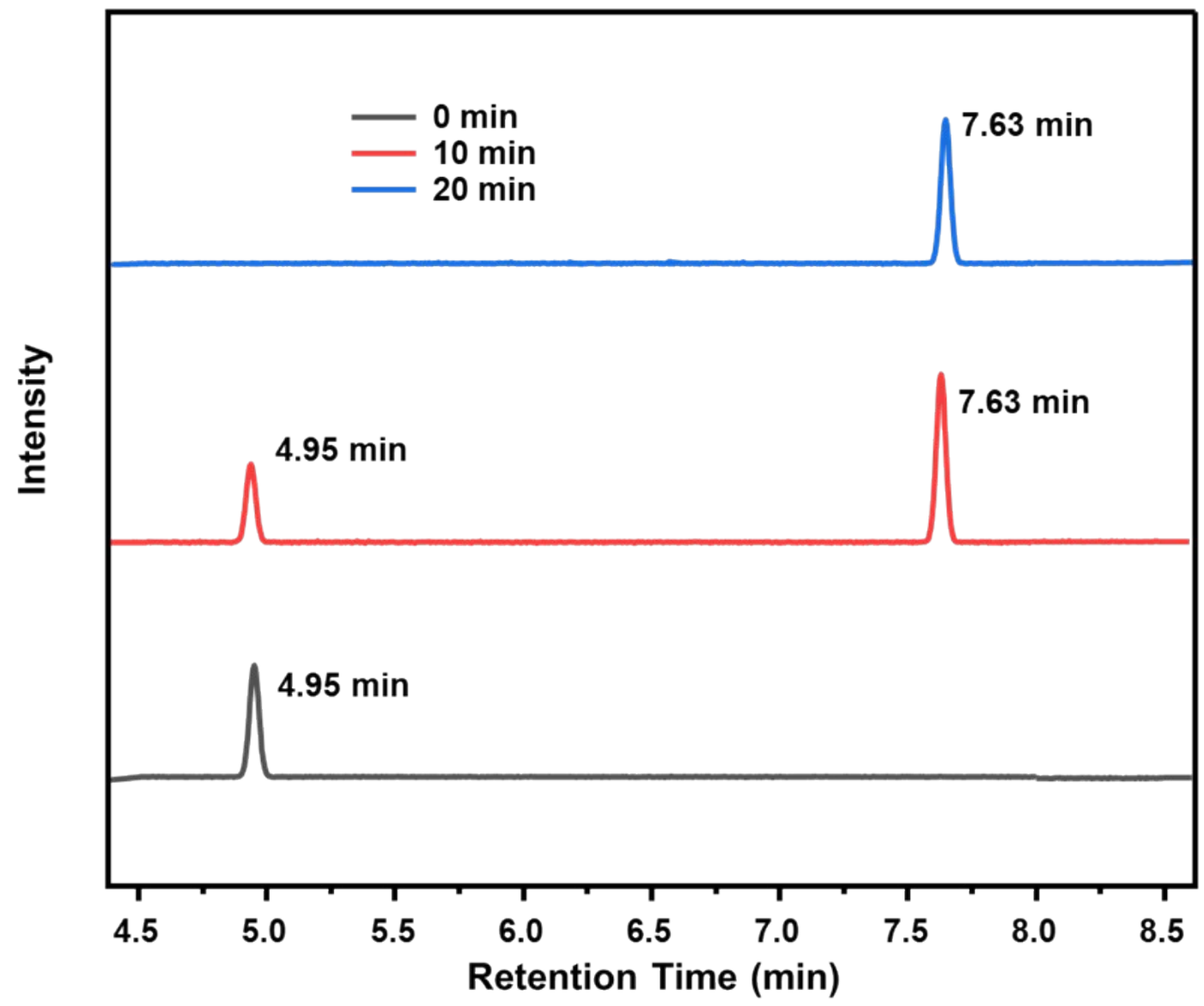

Figure S32. Gas chromatography (GC) signals indicating the progress of the oxidation of CEES to CEESO in the presence of **Por-Cage** at different times.

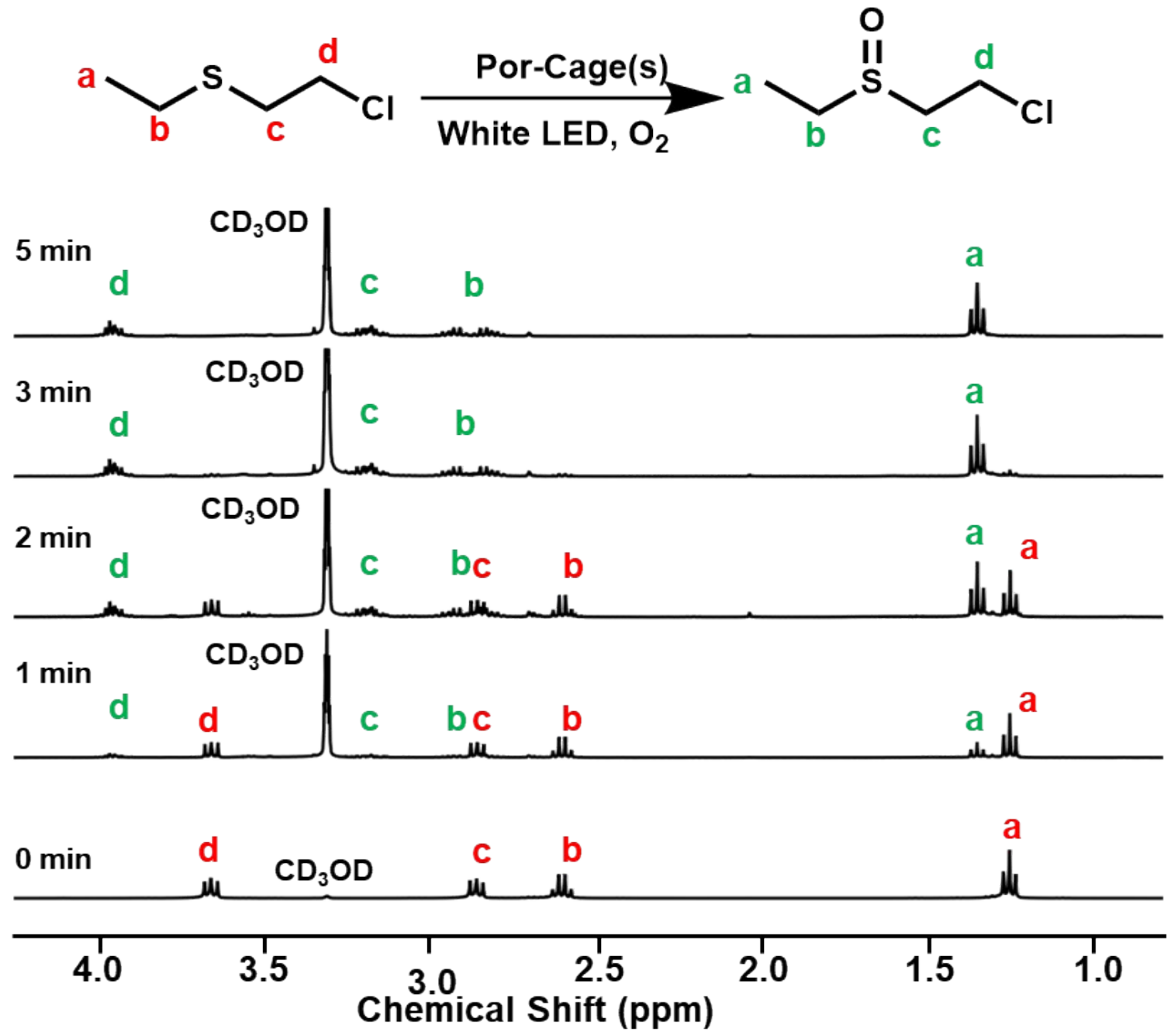

Figure S33. ^{1}H NMR analysis of CEES photo-oxidation by **Por-Cage** powder (400 MHz, CD_3OD, 300 K) at pure oxygen atmosphere.

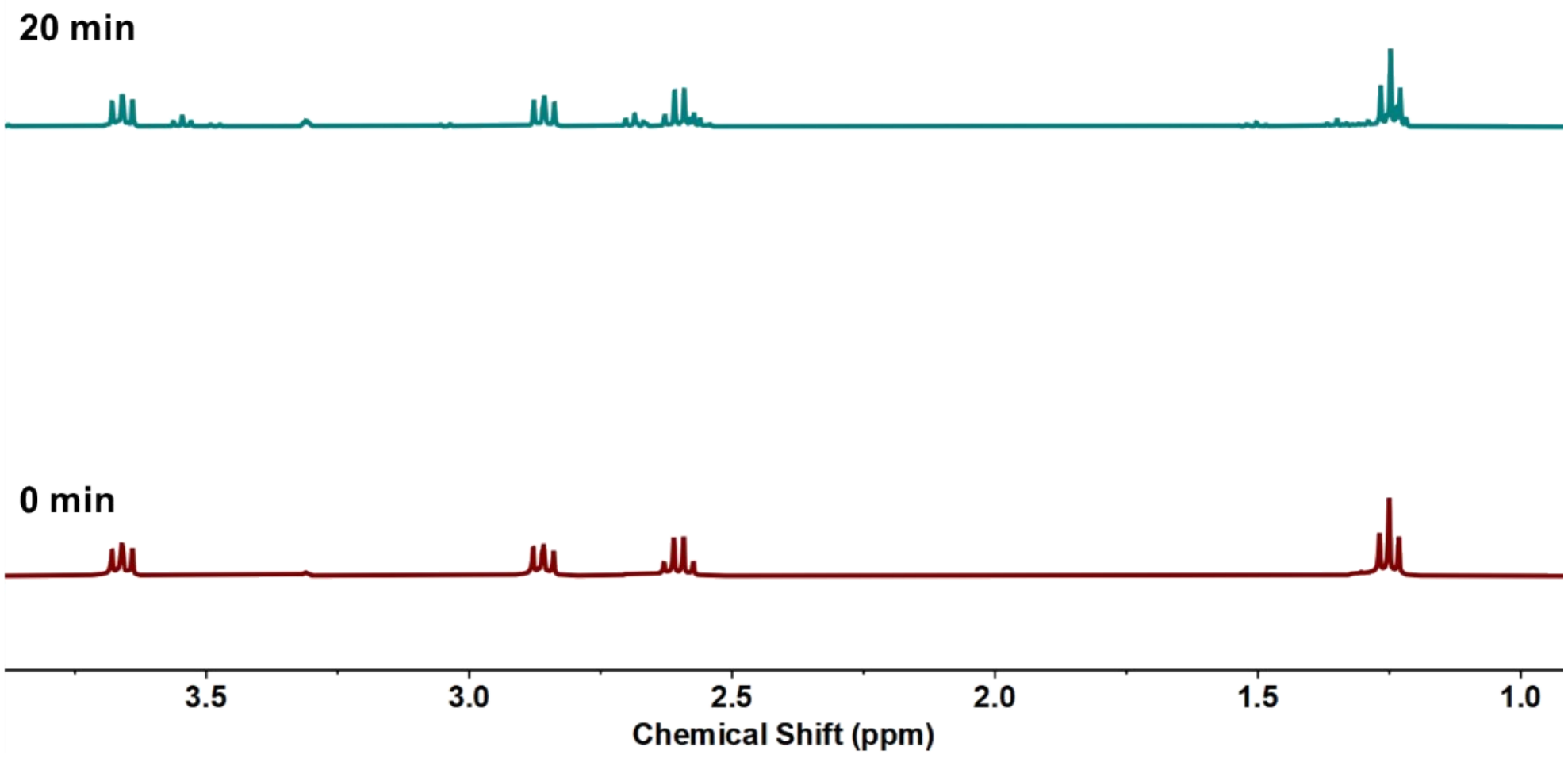

Figure S34. Control experiment for CEES photo-oxidation catalyzed by **Por-Cage** under an Ar atmosphere (500 MHz, CD_3OD, 300 K).

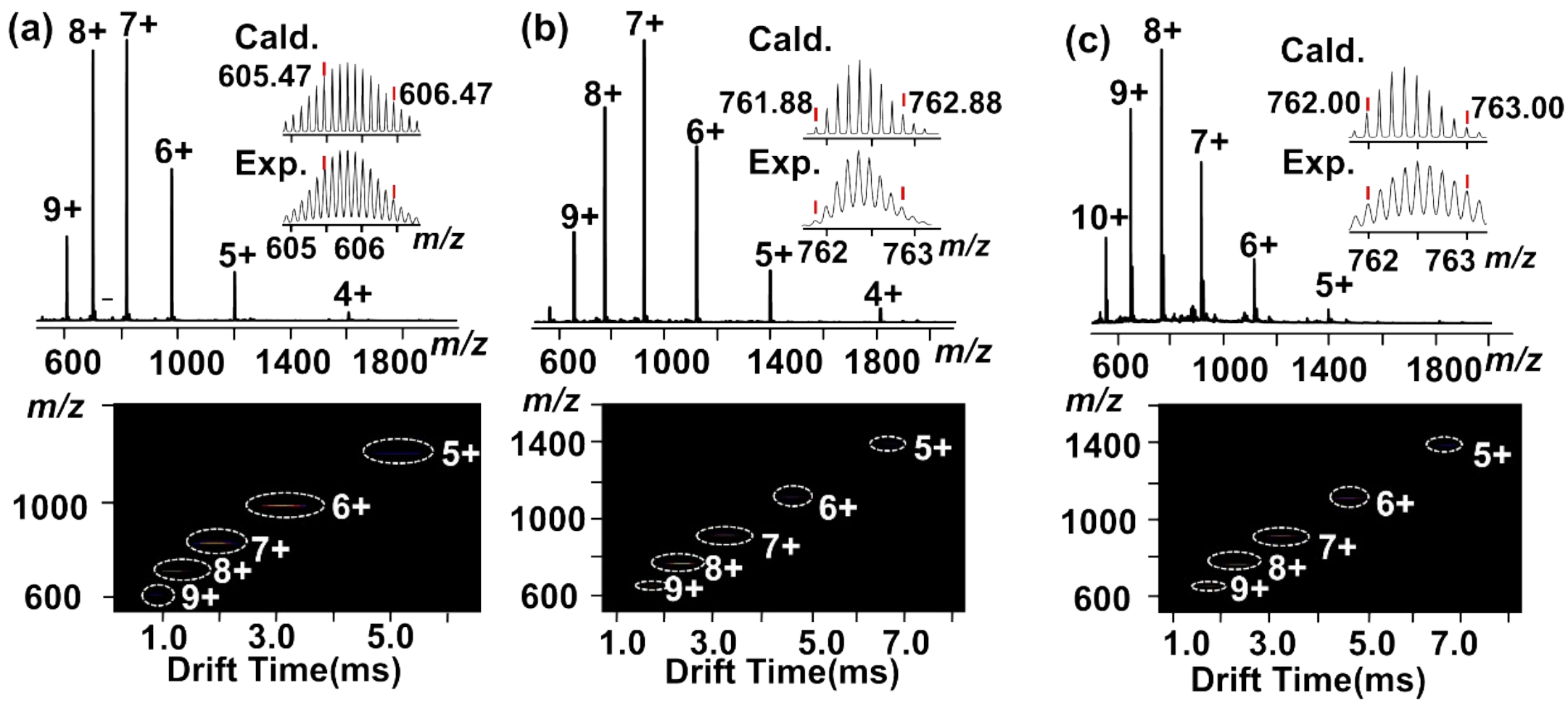

Figure S35. ESI-MS and TWIM-MS of (a) **Por-Cage-Zn**, (b) **Por-Cage-Co**, (c) **Por-Cage-Ni**.

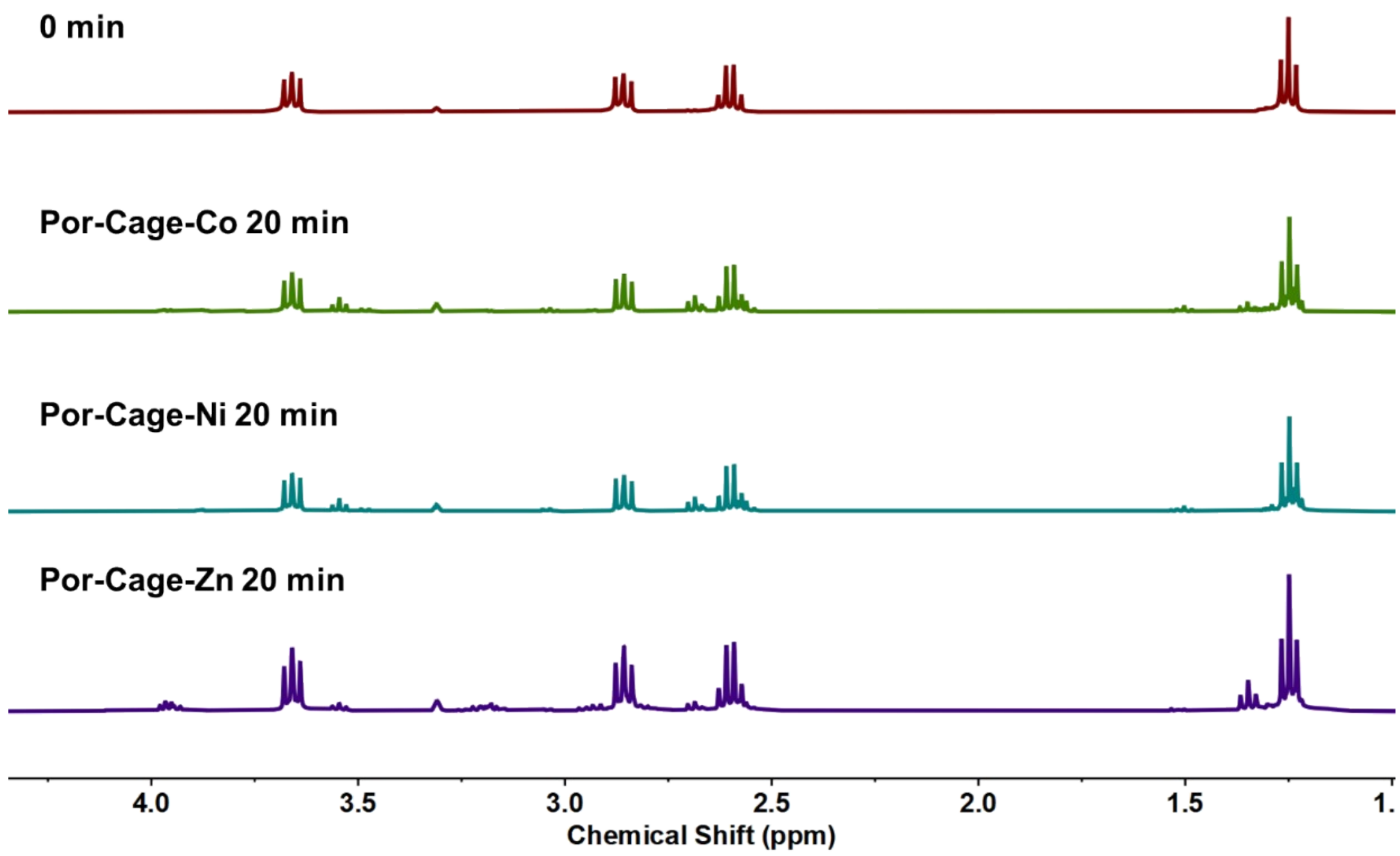

Figure S36. ^{1}H NMR spectrum of the oxidation reaction in the metallo-organic cage **Por-Cage-Co, Por-Cage-Ni, Por-Cage-Zn** heterogeneous photocatalytic CEES system (500 MHz, CD_3OD, 300 K).

Table S3. TON and TOF of **Por-Cage**, **TPP**, **L**.

Catalyst	Porphyrin units (μmol)	CEES (μmol)	t (min)	Conv. (%)	TON (per porphyrin)	TOF (h^{-1})
**Por-Cage**	2.5	250	20	100	100	303.03
**TPP**	2.5	250	20	5.4	5.4	16.36
**L**	2.5	250	20	10.2	10.2	30.91

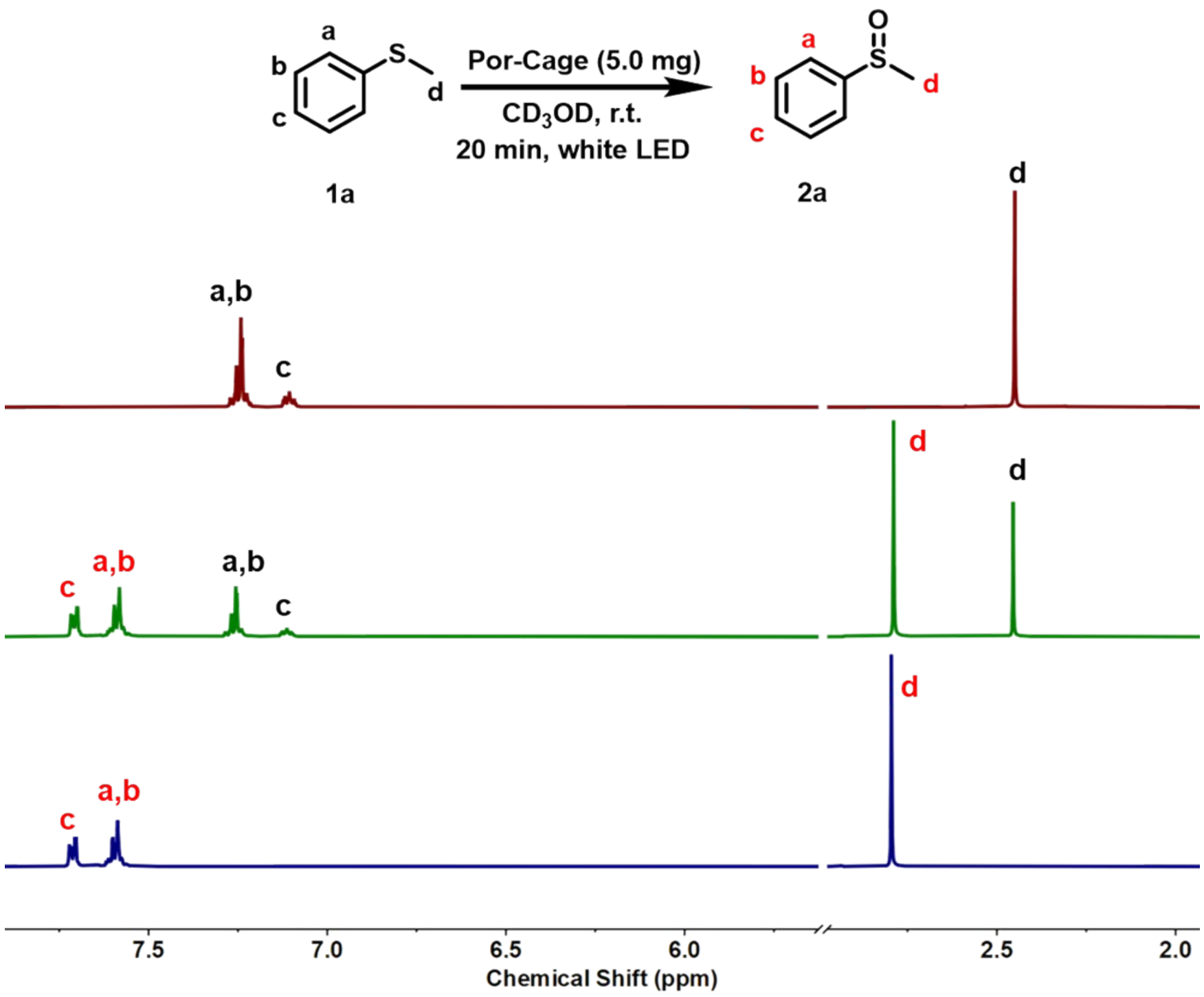

Figure S37. ^{1}H NMR spectrum of the oxidation reaction in the metallo-organic cage **Por-Cage** heterogeneous photocatalytic sulfide ether compound **1a** system.

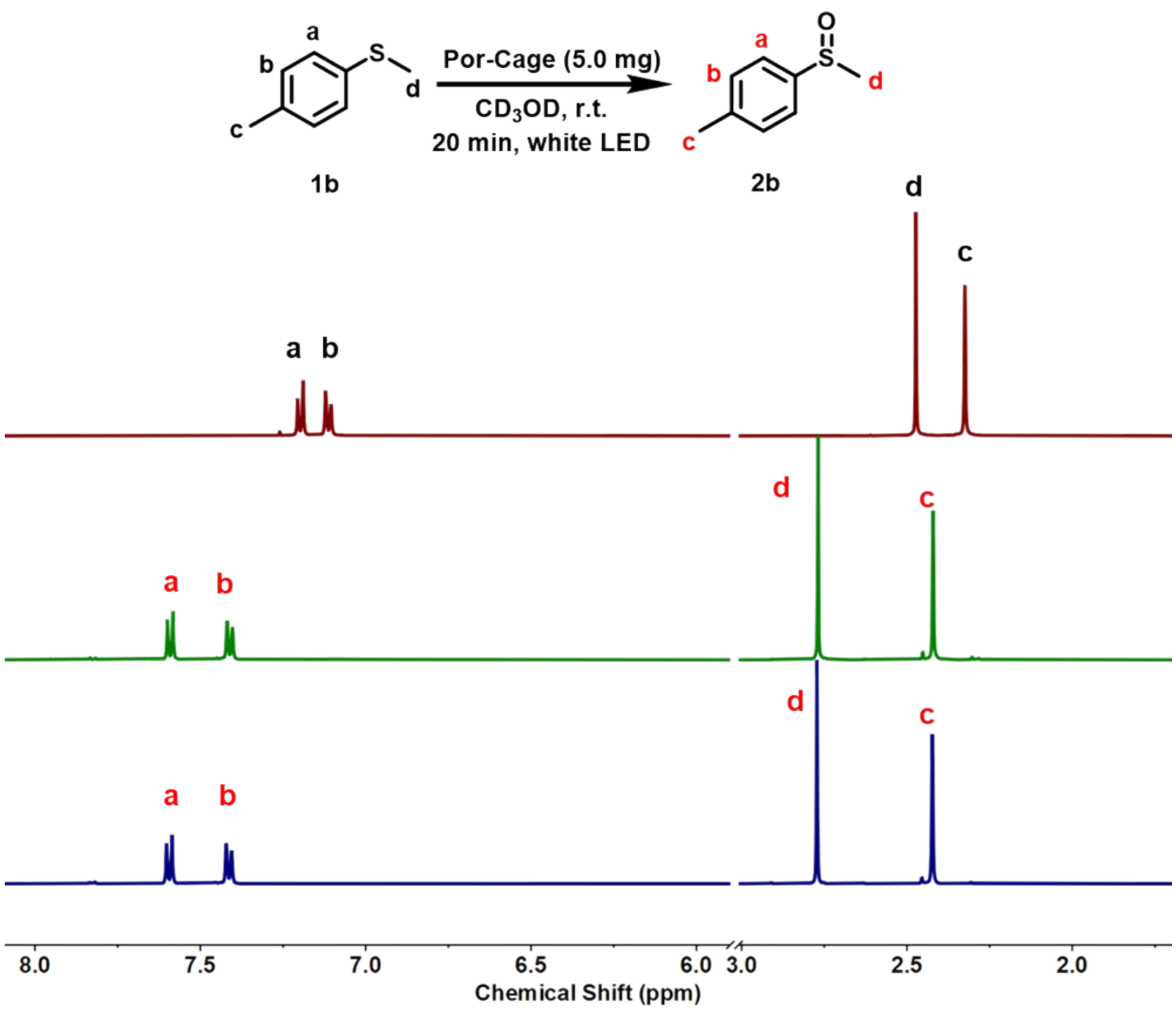

Figure S38. ^{1}H NMR spectrum of the oxidation reaction in the metallo-organic cage **Por-Cage** heterogeneous photocatalytic sulfide ether compound **1b** system.

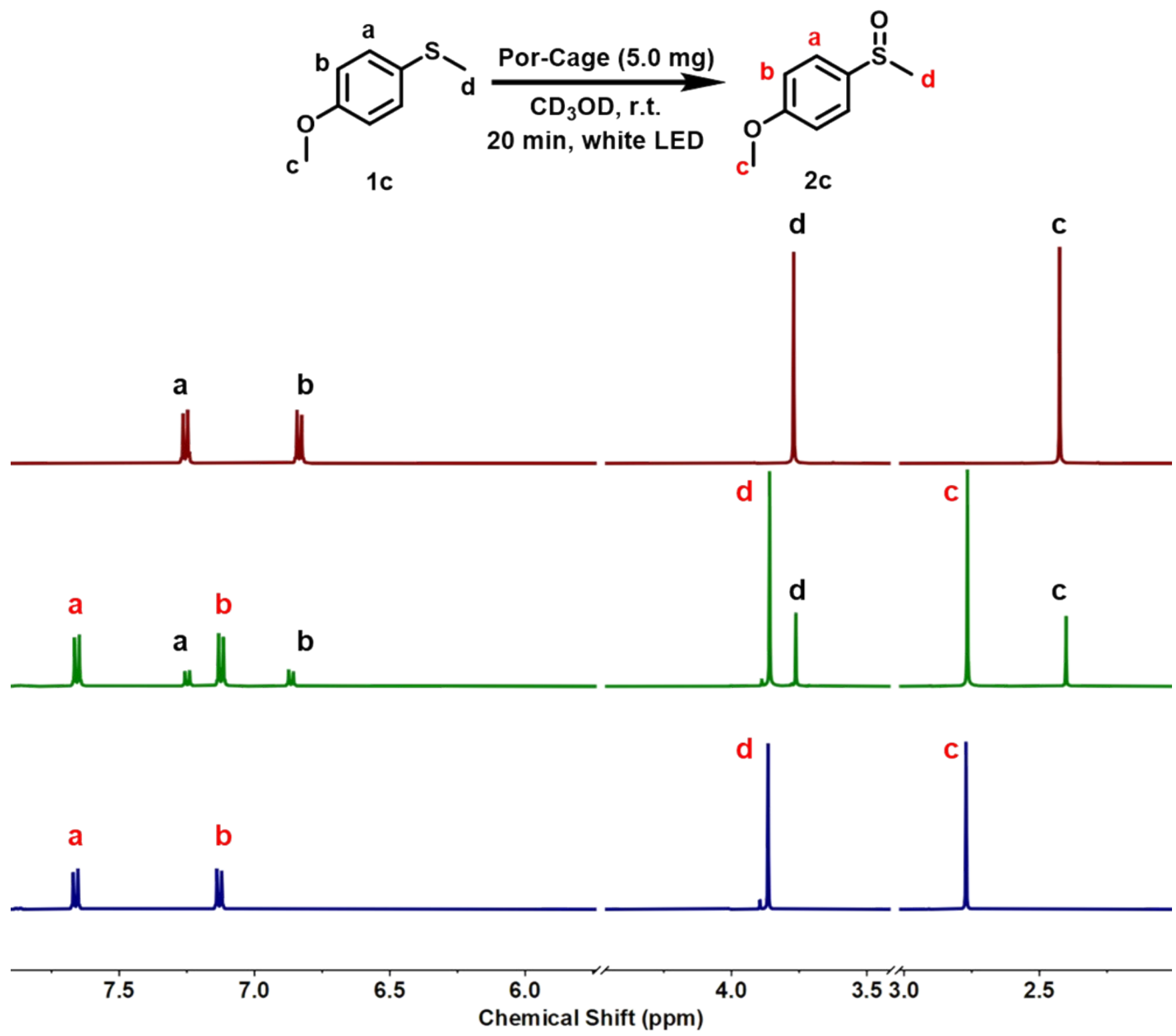

Figure S39. ^{1}H NMR spectrum of the oxidation reaction in the metallo-organic cage **Por-Cage** heterogeneous photocatalytic sulfide ether compound **1c** system.

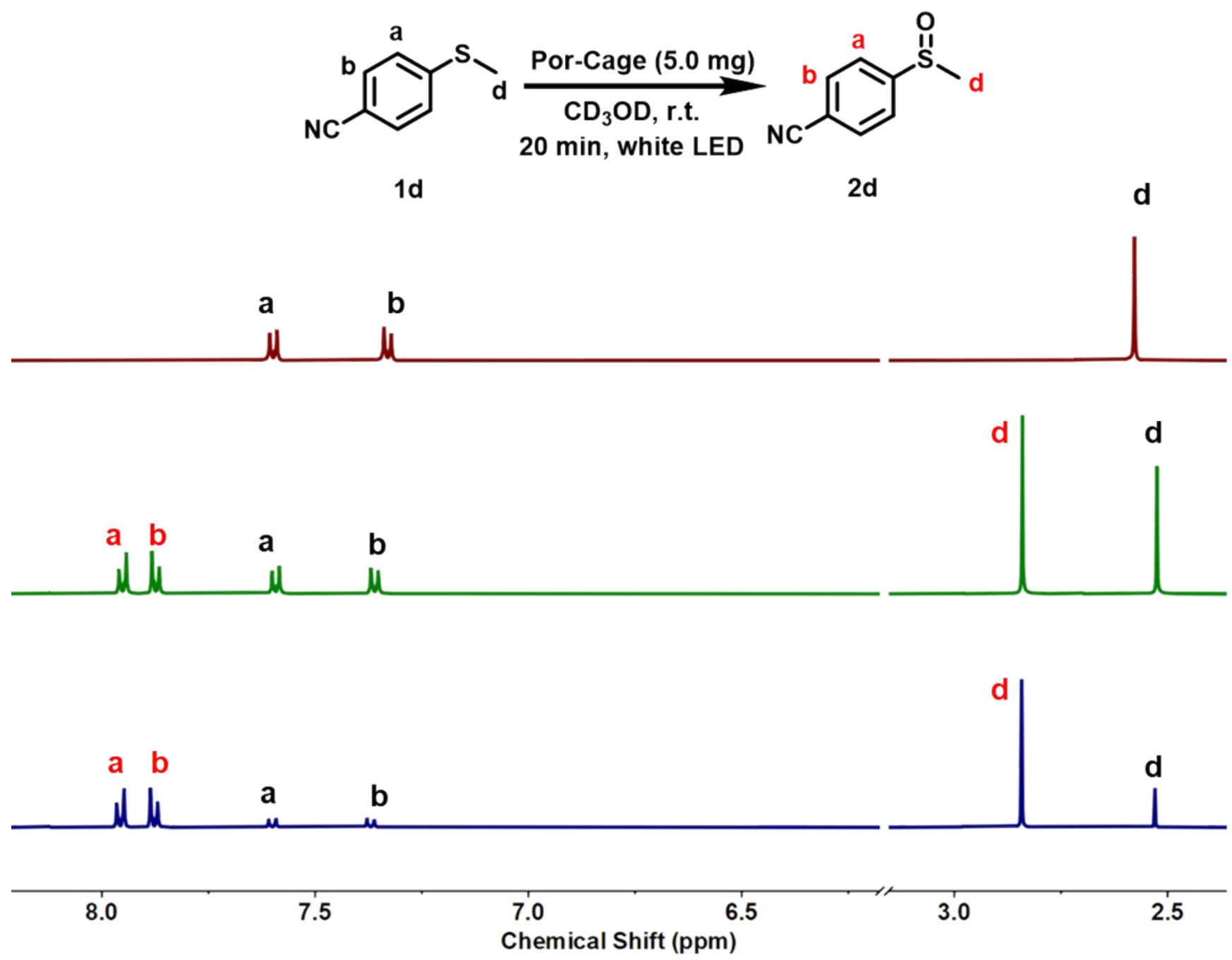

Figure S40. ^{1}H NMR spectrum of the oxidation reaction in the metallo-organic cage **Por-Cage** heterogeneous photocatalytic sulfide ether compound **1d** system.

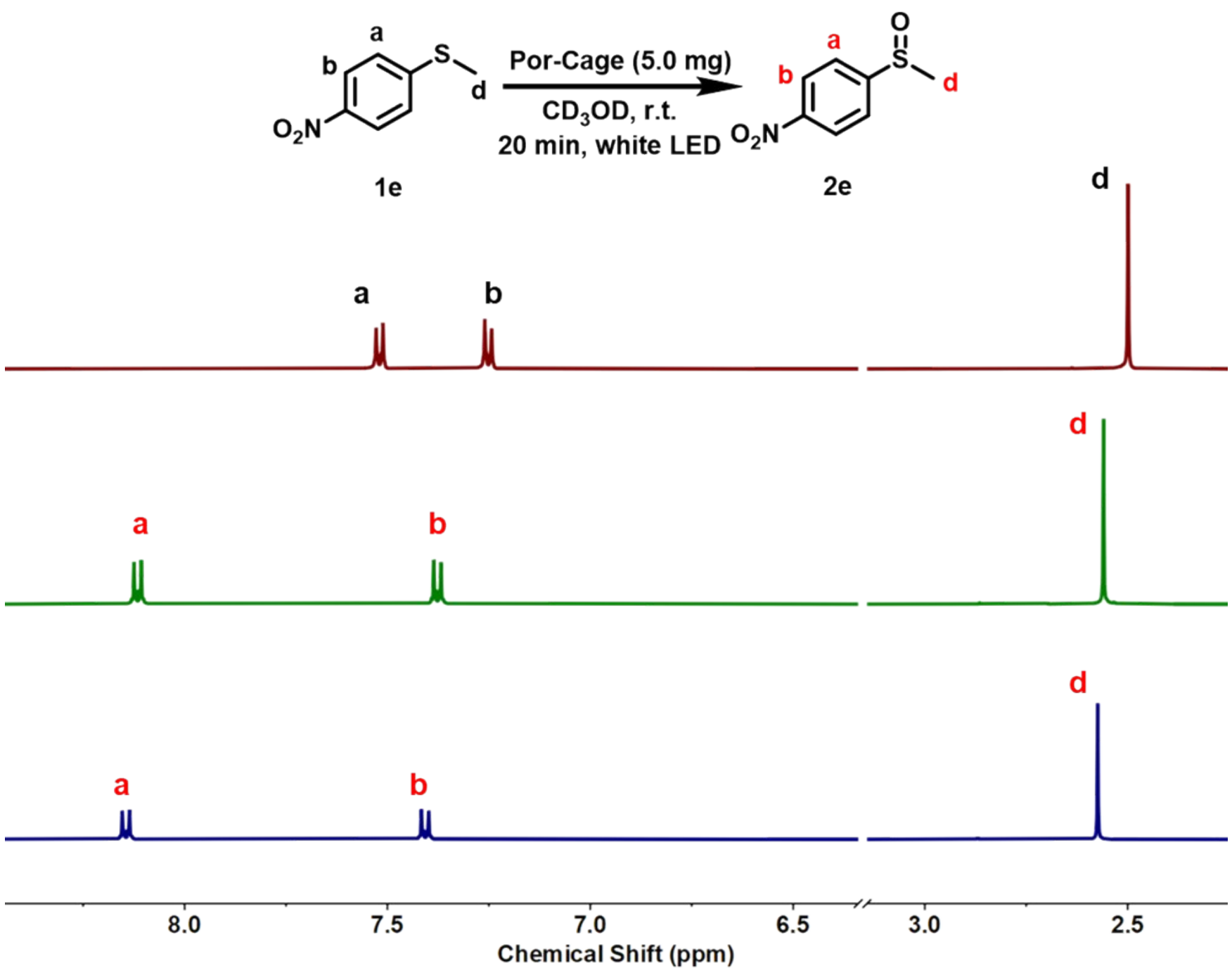

Figure S41. ^{1}H NMR spectrum of the oxidation reaction in the metallo-organic cage **Por-Cage** heterogeneous photocatalytic sulfide ether compound **1e** system.

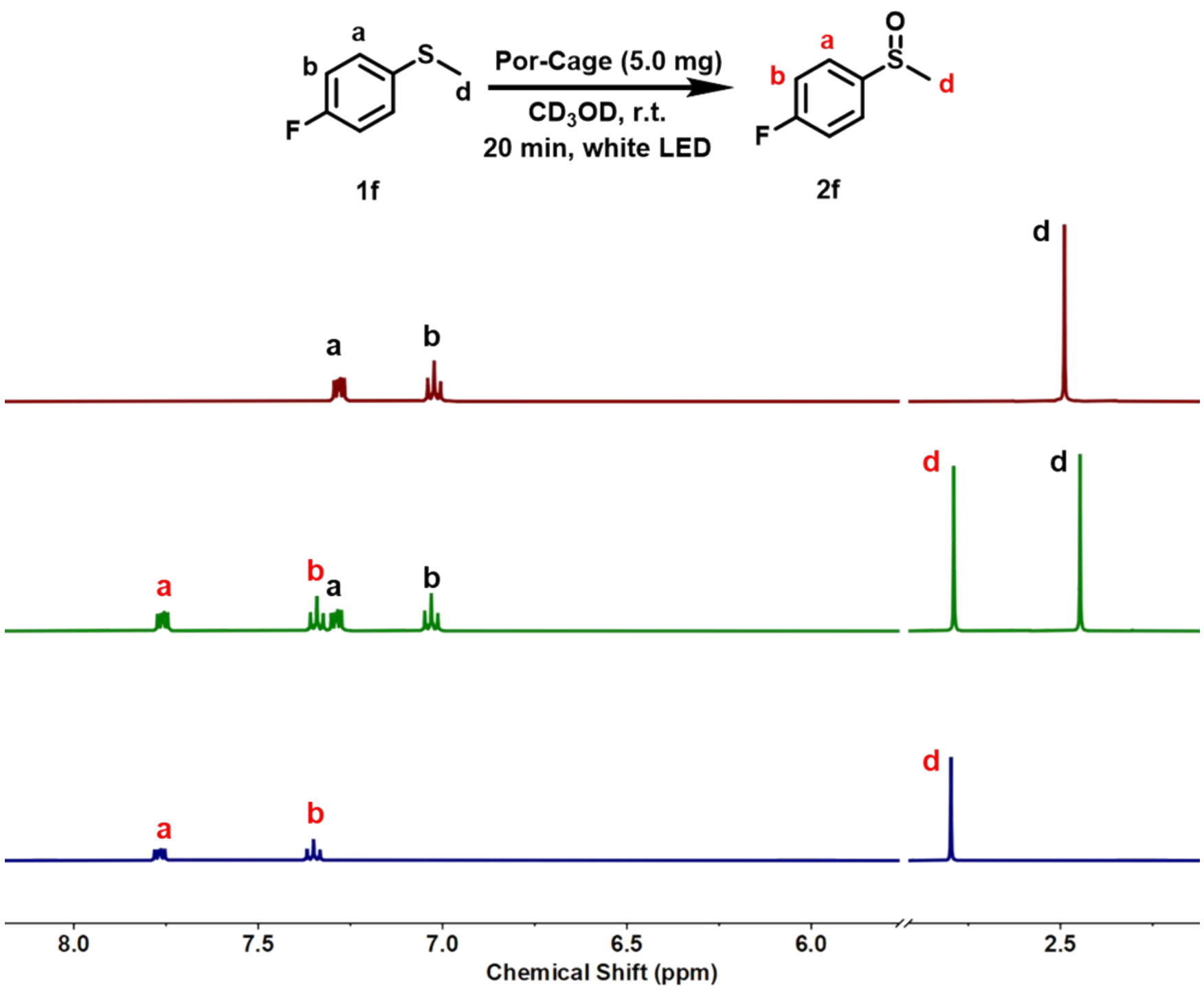

Figure S42. ^{1}H NMR spectrum of the oxidation reaction in the metallo-organic cage **Por-Cage** heterogeneous photocatalytic sulfide ether compound **1f** system.

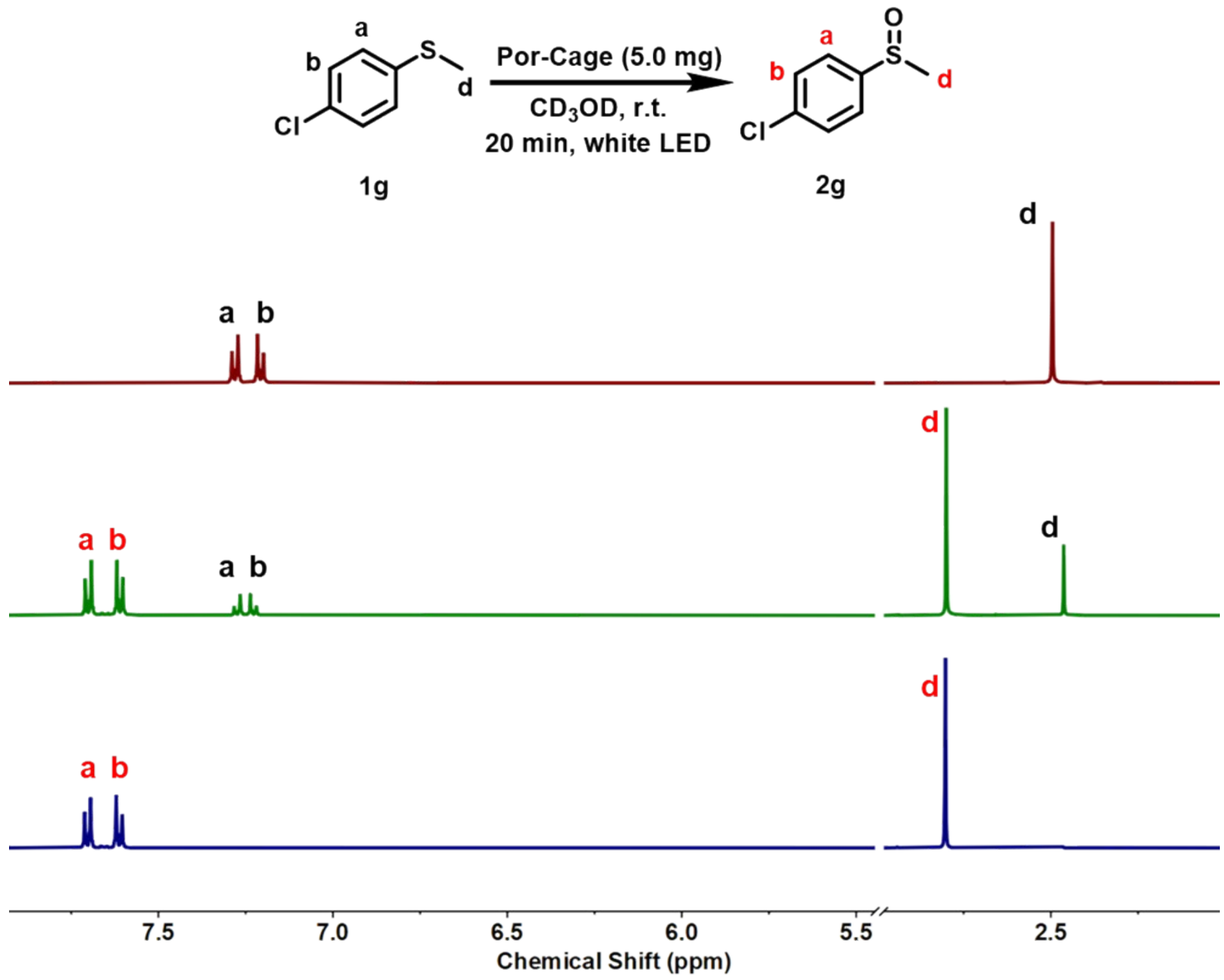

Figure S43. ^{1}H NMR spectrum of the oxidation reaction in the metallo-organic cage **Por-Cage** heterogeneous photocatalytic sulfide ether compound **1g** system.

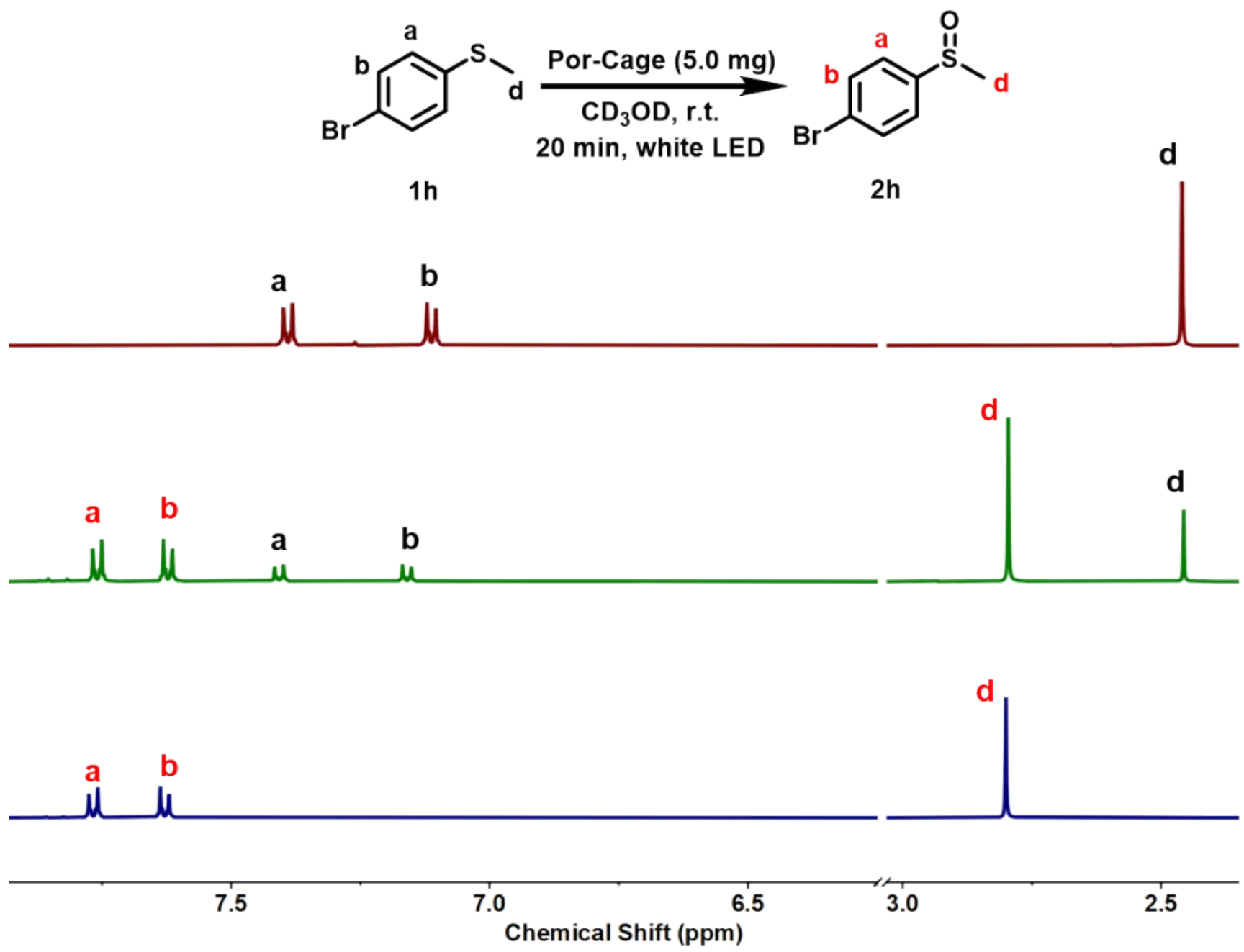

Figure S44. ^{1}H NMR spectrum of the oxidation reaction in the metallo-organic cage **Por-Cage** heterogeneous photocatalytic sulfide ether compound **1h** system.

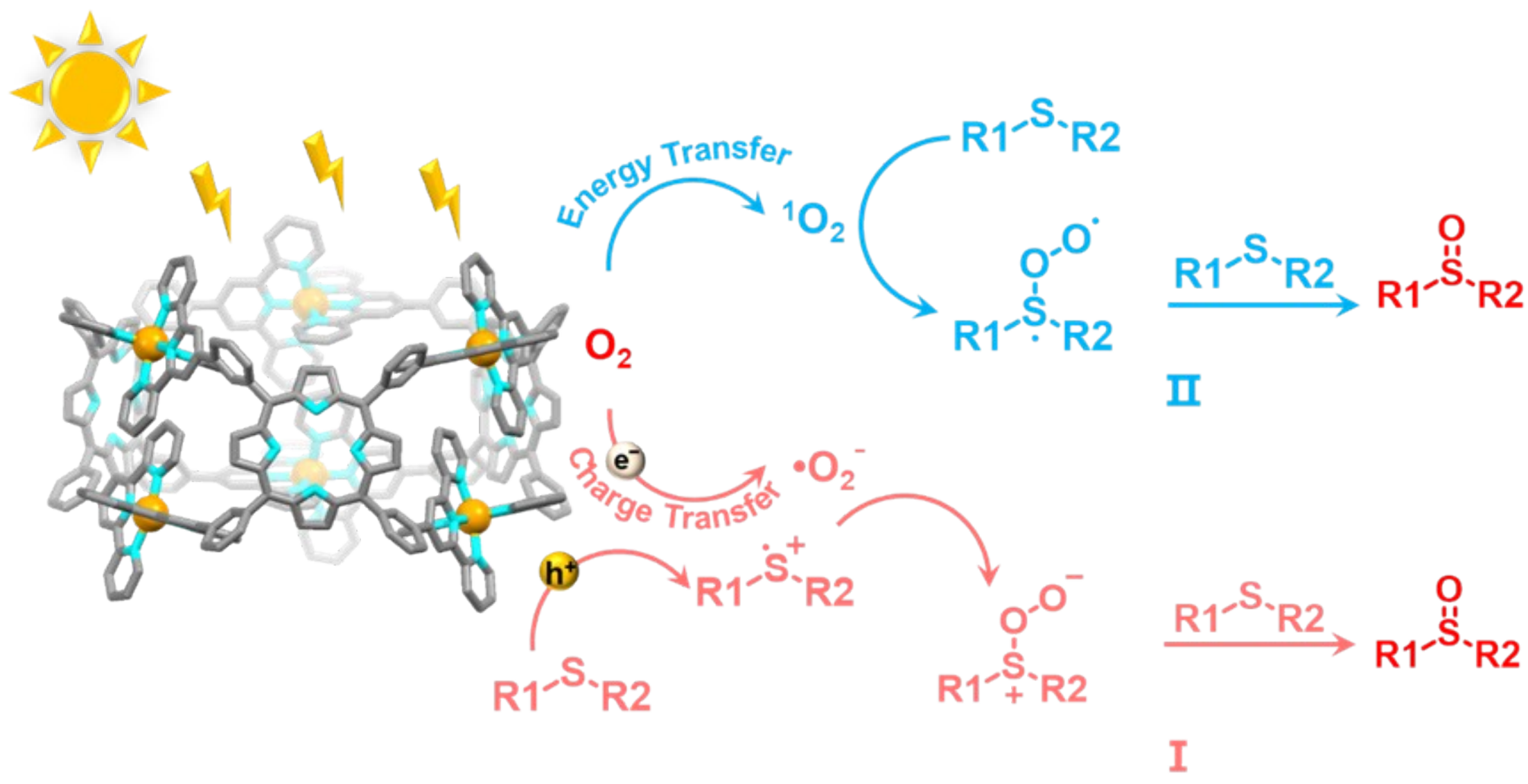

Figure S45. Proposed mechanism for the photocatalytic oxidation of thioanisole over **Por-Cage**.

8. X-ray crystallographic data and structures

Single crystals of **Por-Cage** suitable for X-ray diffraction were obtained by crystallization from CH_3CN/Isopropyl ether (6 mg/mL) at 15 °C. Single-crystals X-ray diffraction data for **Por-Cage** was collected on a Bruker D8 VENTURE diffractometer using a mirror monochromated Ga-Kα radiation. Using Olex2 1.5, the structures were solved with the SIR2004 [2] structure solution program using Direct Methods and refined with the XH [3] refinement package using CGLS minimisation. Data refinement and reduction were undertaken with Bruker SAINT. The structures were solved by direct methods and refined by full-matrix least-squares on F2 with anisotropic displacement using the SHELXTL-97 software package. Details on crystals data collection and refinement were summarized in Table S1. CCDC: 2494623.

.

Table S4. Crystal Data and Structure Refinement for **Por-Cage**.

Identification code	**Por-Cage**
Empirical formula	$C_{312}H_{198}Cd_6N_{48}$
Moiety formula	$C_{312}H_{198}Cd_6N_{48}$
Formula weight	5293.57
Temperature/K	100.00(11)
Crystal system	monoclinic
Space group	P21/c
a/Å	17.160(3)
b/Å	58.032(10)
c/Å	47.378(9)
α/°	90
β/°	99.683(8)
γ/°	90
Volume/Å^3	46509(15)
Z	4
ρ_{calc}g/cm^3	0.756
μ/mm^{-1}	1.663
F(000)	10776.0
Crystal size/mm^3	0.1 × 0.08 × 0.06
Radiation	GaKα (λ = 1.34139)
2Θ range for data collection/°	2.112 to 110.18
Index ranges	-20 ≤ h ≤ 18, -69 ≤ k ≤ 62, -56 ≤ l ≤ 49
Reflections collected	966853
Independent reflections	82417 [R_{int} = 0.1387, R_{sigma} = 0.1748]
Data/restraints/parameters	82417/0/3259
Goodness-of-fit on F^2	3.003
Final R indexes [I>=2σ (I)]	R_1 = 0.4100, wR_2 = 0.7486
Final R indexes [all data]	R_1 = 0.4775, wR_2 = 0.7845
Largest diff. peak/hole / e Å^{-3}	9.43/-4.84

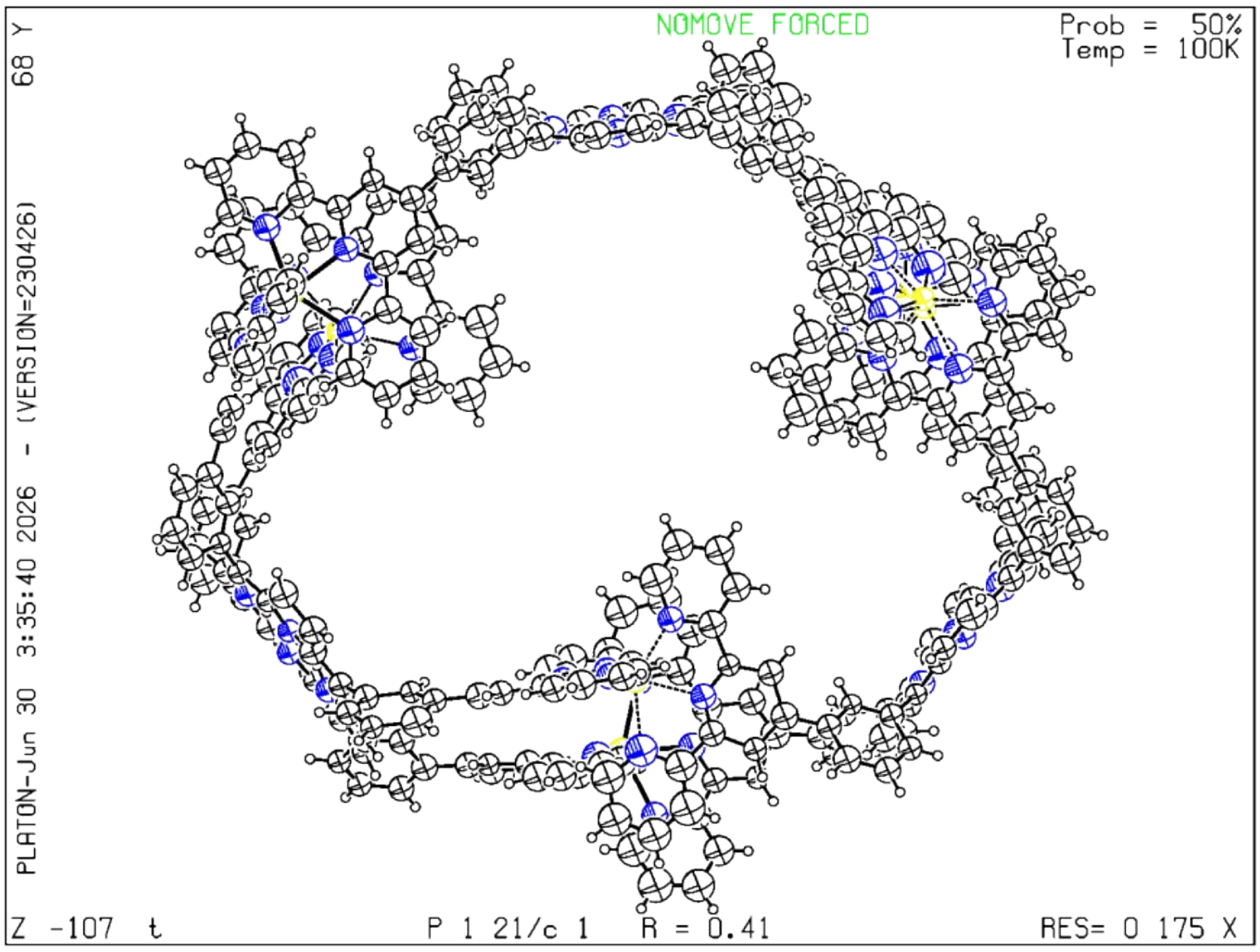

Figure S46. Ortep drawing of the asymmetric unit in the crystal structure of **Por-Cage** at 50% probability level.

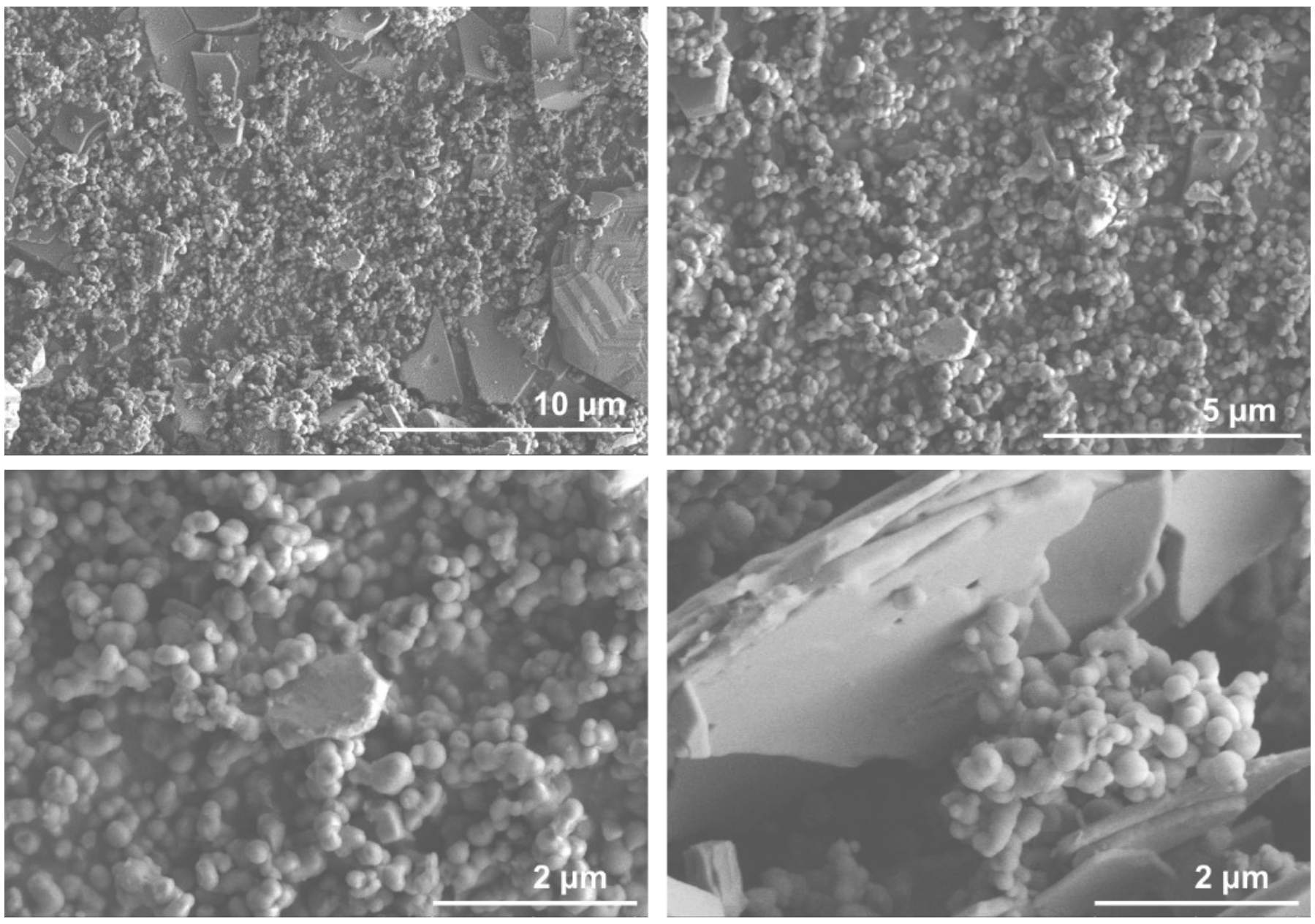

Figure S47. SEM images of the metallo-organic cage **Por-Cage** at different scales.

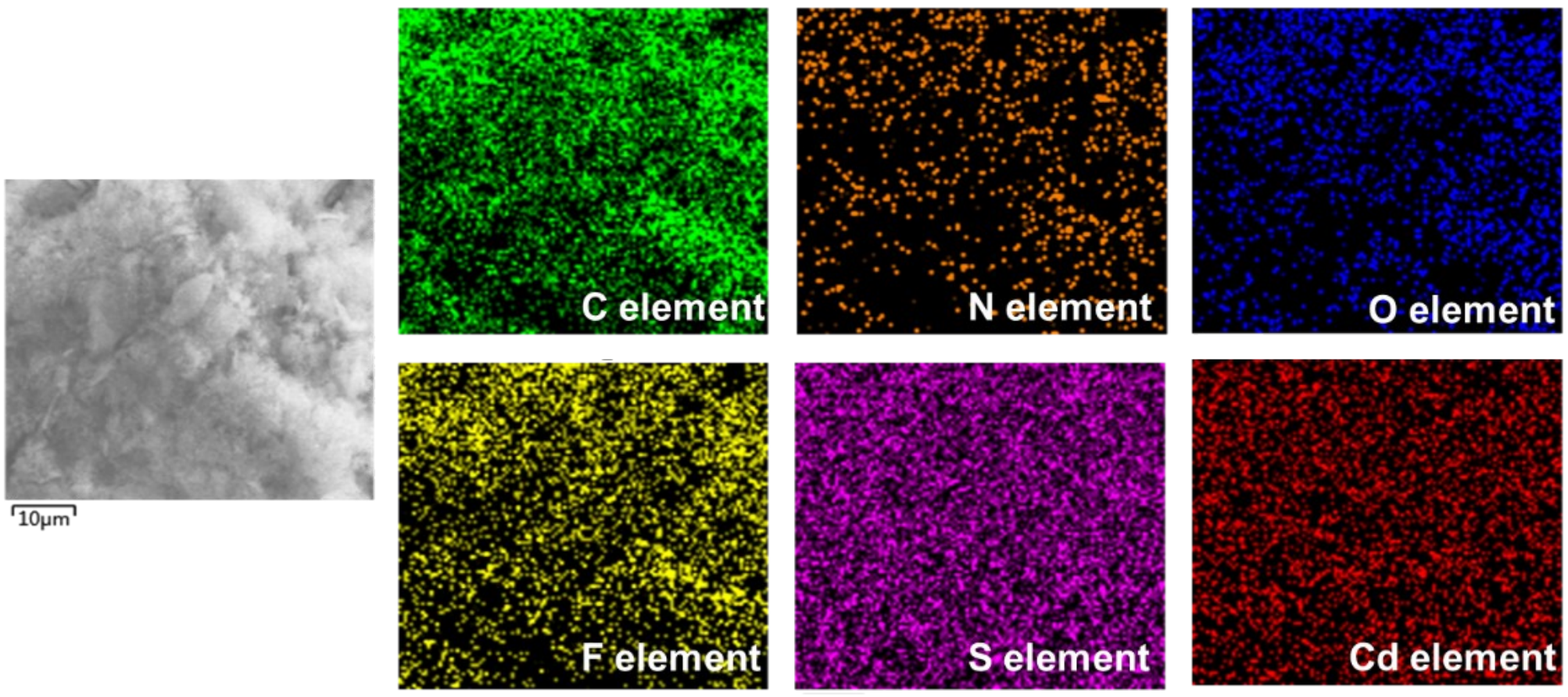

Figure S48. EDS mapping of the metallo-organic cage **Por-Cage**.

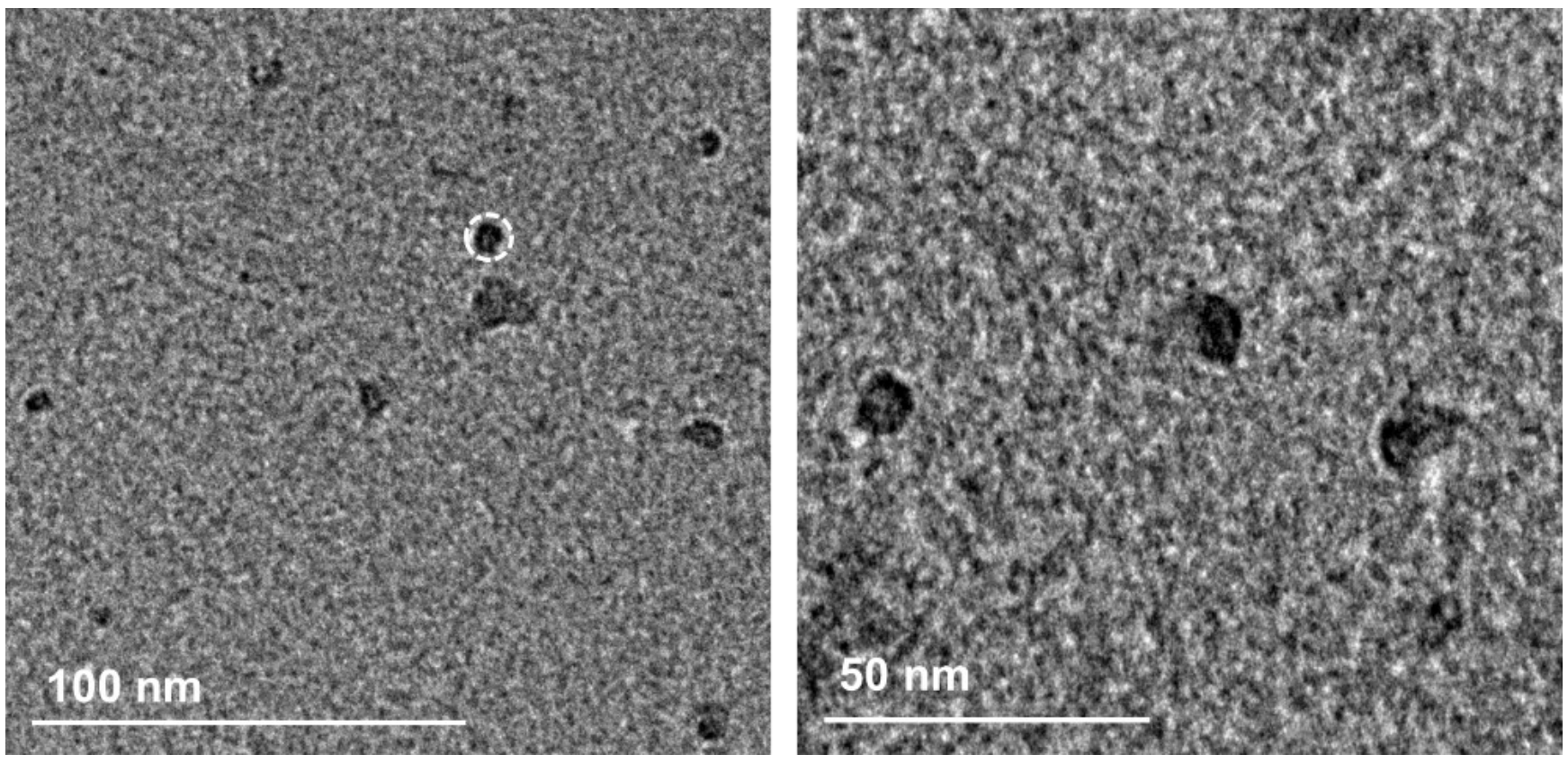

Figure S49. TEM images of the metallo-organic cage **Por-Cage** at different scales.

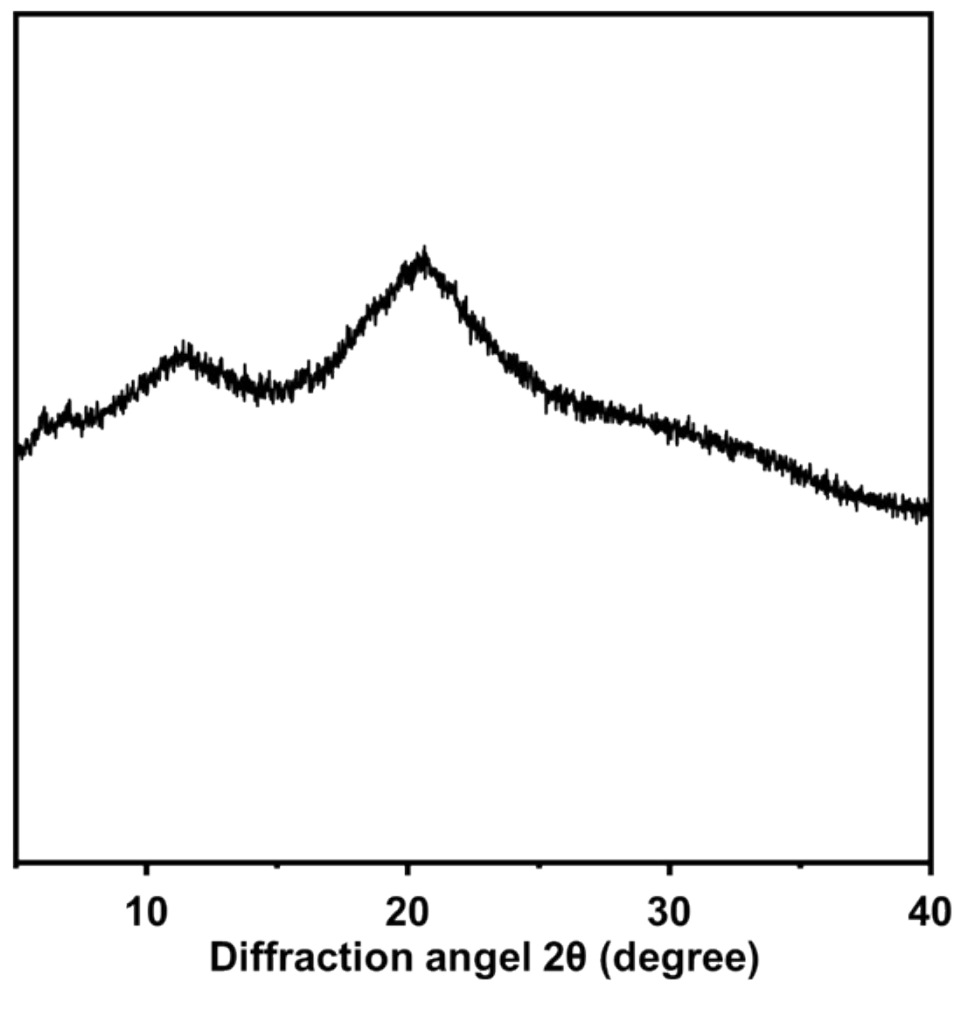

Figure S50. PXRD patterns of metallo-organic cage **Por-Cage**.

9. TD-DFT Calculations

Table S5. The singlet and triplet excited states transition configurations of model **Por-Cage** revealed by TD-DFT calculations.

Structure	Excited state	Excited energy(eV)	Transition configuration (%)
	S1	2.0653	H-1 -> L+10 29.3%, H -> L+11 25.2%, H-3 -> L+8 19.2%, H-2 -> L+9 17.3%
	T1	0.4899	H-1 -> L+8 80.9%, H-3 -> L+10 52.9%, H-1 <- L+8 -37.9%, H-1 -> L+9 37.6%, H-3 <- L+10 -29.0%, H-1 <- L+9 -17.6%
**Por-Cage**	T2	0.4899	H -> L+9 80.9%, H-2 -> L+11 52.9%, H <- L+9 -37.9%, H -> L+8 37.6%, H-2 <- L+11 -29.0%, H <- L+8 -17.6%
	T3	1.2939	H-1 -> L+10 71.2%, H-3 -> L+8 20.9%, H-3 -> L+9 9.7%, H-1 <- L+10 -8.5%
	T4	1.294	H -> L+11 71.2%, H-2 -> L+9 20.9%, H-2 -> L+8 9.7%, H <- L+11 -8.5%

Table S6. The singlet and triplet excited states transition configurations of model **TPP** revealed by TD-DFT calculations.

Structure	Excited state	Excited energy(eV)	Transition configuration (%)
**TPP**	S1	2.2025	H -> L 73.1%, H-1 -> L+1 24.4%
	T1	0.7205	H -> L 101.6%, H <- L -26.7%, H-1 -> L+1 23.8%, H-1 <- L+1 -10.2%
	T2	1.6396	H -> L+1 93.2%
	T3	1.9699	H-1 -> L+1 70.8%, H -> L 11.7%H -> L+2 6.6%
	T4	2.1887	H-1 -> L 87.4%

10. References

1. J.-D. Chai, M. Head-Gordon. Long-range corrected hybrid density functionals with damped atom-atom dispersion corrections. P*hys. Chem. Chem. Phys*. 2008, ***10****(44),* 6615-6620. DOI: 10.1039/B810189B
2. F. Weigend, R. Ahlrichs. Balanced basis sets of split valence, triple zeta valence and quadruple zeta valence quality for H to Rn: Design and assessment of accuracy. Phys. *Chem. Chem. Phys. 2005*, *7(18),* 3297-3305. DOI: 10.1039/B508541A
3. F. Weigend. Accurate coulomb-fitting basis sets for H to Rn. *Phys. Chem. Chem. Phys*. 2006, ***8****(9),* 1057-1065. DOI: org/10.1039/B515623H
4. T. Lu, F. Chen. Multiwfn: A multifunctional wavefunction analyzer. *J. Comput. Chem*. 2012, ***33****(5)*, 580-592. DOI: org/10.1002/jcc.22885
5. T. Lu. A comprehensive electron wavefunction analysis toolbox for chemists, multiwfn. *J. Comput. Chem*. 2024, ***161****(8)*, 082503. DOI: org/10.1063/5.0216272
6. W. Humphrey, A. Dalke, K. Schulten. Vmd: Visual molecular dynamics. *J. Mol. Graph*. 1996, ***14****(1),* 33-38. DOI: 10.1016/0263-7855(96)00018-5
7. L. V. Lutkus, S. S. Rickenbach, T. M. McCormick. Singlet oxygen quantum yields determined by oxygen consumption. *Journal of Photochemistry and Photobiology A: Chemistry*. 2019, ***378****,* 131-135. DOI: 10.1016/j.jphotochem.2019.04.029
